# Genomes to natural products PRediction Informatics for Secondary Metabolomes (PRISM)

**DOI:** 10.1093/nar/gkv1012

**Published:** 2015-10-05

**Authors:** Michael A. Skinnider, Chris A. Dejong, Philip N. Rees, Chad W. Johnston, Haoxin Li, Andrew L. H. Webster, Morgan A. Wyatt, Nathan A. Magarvey

**Affiliations:** Departments of Biochemistry and Biomedical Sciences and Chemistry and Chemical Biology, Michael G. DeGroote Institute for Infectious Disease Research, McMaster University, Hamilton, ON, L8S 4K1, Canada

## Abstract

Microbial natural products are an invaluable source of evolved bioactive small molecules and pharmaceutical agents. Next-generation and metagenomic sequencing indicates untapped genomic potential, yet high rediscovery rates of known metabolites increasingly frustrate conventional natural product screening programs. New methods to connect biosynthetic gene clusters to novel chemical scaffolds are therefore critical to enable the targeted discovery of genetically encoded natural products. Here, we present PRISM, a computational resource for the identification of biosynthetic gene clusters, prediction of genetically encoded nonribosomal peptides and type I and II polyketides, and bio- and cheminformatic dereplication of known natural products. PRISM implements novel algorithms which render it uniquely capable of predicting type II polyketides, deoxygenated sugars, and starter units, making it a comprehensive genome-guided chemical structure prediction engine. A library of 57 tailoring reactions is leveraged for combinatorial scaffold library generation when multiple potential substrates are consistent with biosynthetic logic. We compare the accuracy of PRISM to existing genomic analysis platforms. PRISM is an open-source, user-friendly web application available at http://magarveylab.ca/prism/.

## INTRODUCTION

Natural products represent the basis for the majority of small molecule drugs currently in clinical use, due in part to their diverse and unique chemical scaffolds (1). Microbes often synthesize these complex small molecules via modular strategies that create combinatorial pools, and natural selection selects those optimized for biological activity (2). Despite great success in the past, bioactivity-guided screening of microbial extracts for natural product discovery is increasingly met with failure characterized by high rediscovery rates (3). These screening outcomes are at odds with genomic analysis, which suggests that as few as 10% of genetically encoded secondary metabolites are known (4). The exponential increase in microbial gene sequence information attendant on advances in next-generation sequencing technology has revealed a wealth of natural product biosynthetic gene clusters. However, natural product discovery has not kept pace with the availability of genomic information. A major impediment to genome-guided natural product discovery is the need for accurate methodologies to translate biosynthetic gene sequences into useful chemical information.

The majority of bioactive natural products are produced by large, multi-domain enzymes or enzyme complexes known as polyketide synthases (PKSs) and nonribosomal peptide synthetases (NRPSs) (5). Extensive characterization of the biosynthetic pathways of these enzymes has enabled the prediction of certain structural elements of the corresponding polyketide and nonribosomal peptide products from protein sequence data (6). Accordingly, several methods to enable the prediction of individual monomers (7–13) and chemical structures (14–16) have been presented. However, the design of existing methodologies places inherent limitations on the accuracy and scope of structure prediction. In particular, existing methods emphasize the detection of biosynthetic gene clusters over *de novo* chemical structure prediction from identified genetic information (16,17). Many natural products, for instance, contain starter units which mediate biological activity, including long- and short-chain fatty acids, aromatic and alicyclic acids and amino acid derivatives (18), but these are not accounted for by existing methods. Natural products also frequently contain highly specialized deoxysugar appendages which are required for biological activity (19), but no methods exist to automate their prediction. Moreover, no automated method is available to predict the chemical structures of type II polyketides, a large and clinically important family of natural products, from genetic information. Finally, with the exception of NP.searcher (15), which glucosylates free hydroxyls, existing structure prediction methods do not account for the large inventory of enzymatic tailoring reactions which generate enormous structural diversity from a small set of primary metabolites (2).

**Figure 1.**
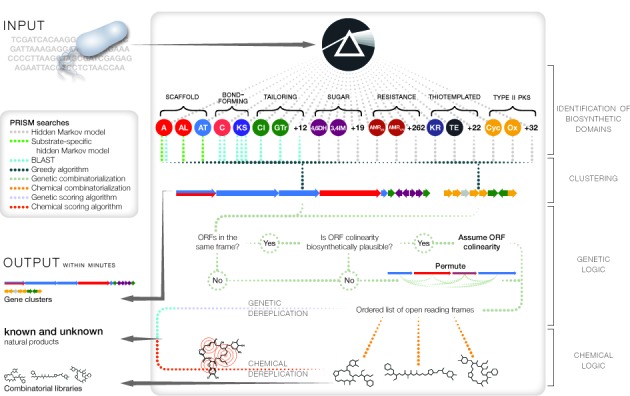
PRISM workflow for genomic prediction of secondary metabolomes. Open reading frames are analyzed with a library of hundreds of hidden Markov models and curated BLAST databases, and identified biosynthetic domains are grouped into clusters. *Trans*-acting adenylation and acyltransferase domains are accounted for as a list of biosynthetically plausible open reading frame permutations is generated. For each ordered list of open reading frames, a set of combinatorial plans is generated based on potential deoxysugar combinations, tailoring reactions and macrocyclization patterns. The resulting combinatorial scaffold library is cheminformatically dereplicated with reference to a database of 49 860 known natural products while the cluster is dereplicated using a multilocus sequence typing-style algorithm with reference to a database of 587 known biosynthetic gene clusters. Unknown natural products are identified by the process of elimination.

Here, we present PRISM (PRediction Informatics for Secondary Metabolomes), an open-source web application for the genomic prediction and bio- and cheminformatic dereplication of nonribosomal peptide and type I and II polyketide chemical structures (Figure 1). PRISM implements a library of 479 hidden Markov models to identify enzyme domains associated with natural product biosynthesis and resistance, and groups them into putative biosynthetic gene clusters. After accounting for the possibility of *trans*-acting acyltransferase or adenylation domains, biosynthetically plausible open reading frame permutations are generated. Monomers are identified with libraries of profile hidden Markov models for adenylation, acyltransferase and acyl-adenylating domains, and a library of 57 virtual reactions is leveraged in order to generate a combinatorial library of natural product scaffolds based on the identified genetic information. The design of the algorithm accounts for all combinations of enzyme substrates consistent with known biosynthetic logic in order to maximize the chance of obtaining a close match to known natural products. The set of predicted chemical structures is compared to a database of 49 860 known natural products via the Tanimoto coefficient in order to cheminformatically dereplicate known natural products. A separate dereplication algorithm uses identified biosynthetic domains to compare putative clusters to a database of 587 sequenced biosynthetic gene clusters. We describe the logic and implementation of PRISM, and compare the accuracy of its structure prediction and cluster dereplication algorithms to available genomic natural products analysis platforms.

## MATERIALS AND METHODS

PRISM is a Java 7 web application built for the Apache Tomcat 7 web server. PRISM implements BLAST (version 2.2.25+) (20) to assess sequence homology, HMMER (version 3.1) (21) for hidden Markov model searches, the Chemistry Development Kit (version 1.4.19) (22) for chemical abstractions, BioJava (version 3.0.7) (23) for sequence translation, RDKit (version 2014.03.1) for Tanimoto coefficient computation, and Apache Batik (version 1.7) for vector image generation.

### General methods

#### Hidden Markov model construction

Sequences were manually collected based on literature reviews, aligned using MUSCLE (version 3.8.31) (24), and trimmed using trimAl (version 1.2rev59) (25) to remove gaps. Hidden Markov models were generated from the resulting trimmed alignments using the hmmbuild program (version 3.1b1), from the HMMER software package (21). Bitscore cutoffs for each hidden Markov model were determined by manual analysis of the results of a search of the UniProtKB database (26), using the HMMER web server (27).

#### Phylogenetic analysis

Sequences were aligned using MUSCLE (version 3.8.31) (24), and manually refined and masked in Mesquite (version 2.75) at the amino acid level. RAxML (version 7.4.2) (28) was used to create phylogenetic trees, performing 100 bootstraps with a gamma distribution. Maximum likelihood analyses were based on the LG substitution model with empirical base frequencies. The appropriate genetic model was determined using the ProtTest package (version 3.2) (29). Trees were visualized using Figtree (version 1.4.0) and stylized in Adobe Illustrator CS6.

### Biosynthetic gene cluster identification

#### File input and options

PRISM accepts nucleotide sequence input in FASTA and GenBank format. The PRISM web server accepts files up to 50 MB. Only the 1000 largest contigs are considered in sequence files containing over 1000 contigs, and contigs under 500 nucleotides in length are discarded. By default, only a file input dialog and the option to enable or disable natural product dereplication are displayed. However, more advanced options can optionally be configured, including the maximum size of combinatorial scaffold libraries, the Tanimoto and homology cutoffs required to dereplicate known natural products, the clustering window, and the number of homologous clusters, similar natural products, substrates, and BLAST results PRISM displays for each cluster or biosynthetic domain.

#### Biosynthetic domain detection

**Figure 2.**
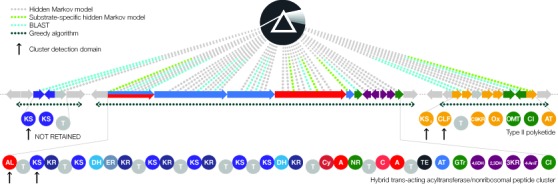
Identification of biosynthetic gene clusters in PRISM. Biosynthetic domains are grouped into clusters using a greedy algorithm. Putative thiotemplated clusters are discarded if they do not contain at least one substrate-activating (A, AL, AT) domain and one bond-forming (C, KS) domain. Putative type II polyketide clusters are discarded if they do not contain a ketosynthase α (KS_α_) and a chain length factor (CLF).

All potential open reading frames are translated and analyzed with a library of 479 hidden Markov models, including models corresponding to 24 thiotemplated domains, 68 adenylation domain substrates, 15 acyltransferase domain substrates, 26 acyl-adenylating domain substrates, 10 domains for the biosynthesis of unusual substrates, 14 tailoring domains, 3 beta lactam-specific domains, 34 type II polyketide domains, 21 deoxysugar biosynthesis domains and 264 resistance domains (Figure 2). Condensation, ketosynthase, chlorination, glycosyltransferase, priming acyltransferase, and chain length factor domains are subsequently analyzed with curated BLAST databases. Selected hidden Markov models were obtained from PFAM (30) (adenylation, condensation, nitroreductase and thiolation) and SMART (31) (acyltransferase, ketosynthase, ketoreductase, dehydratase, enolreductase, and thioesterase). N-, O- and C-methyltransferase hidden Markov models were constructed based on multiple sequence alignments reported by Ansari *et al*. (32). Hidden Markov models for five clades of product template domains were constructed based on phylogenetic trees reported by Li *et al*. (33), while models for fungal starter unit-acyl carrier protein transacylases specific to acetyl and hexanoyl chains were constructed based on a phylogenetic analysis reported by Crawford *et al*. (34). Four glycopeptide P450 hidden Markov models were obtained from CYPED (35). Libraries of profile hidden Markov models for adenylation and acyltransferase domains were obtained from Khayatt *et al*. (12), and supplemented with new models for 3-hydroxyanthalinic acid, β-hydroxyasparagine, β-hydroxyaspartate, capreomycidine, diaminopropionate, dehydroaminobutyrate, β-methylglutamate, histidine, β-hydroxyleucine, methionine, β-methylphenylalanine, β-hydroxyphenylalanine, 3-hydroxypipecolic acid, piperazic acid, and proline. Condensation and ketosynthase domain BLAST databases were obtained from NaPDoS (36). The NaPDoS ketosynthase database was expanded to include additional ketosynthase sequences from enediyne PKSs. 164 hidden Markov models for resistance determinant domains were obtained from Resfams (37). All other hidden Markov models and BLAST databases were developed for this study. A complete list of hidden Markov models and BLAST databases used in this study is presented in Supplementary Dataset 1. All hidden Markov models and BLAST databases used in PRISM are available at http://magarveylab.ca/Skinnider_etal/models/.

Identified adenylation, acyl-adenylating, and acyltransferase domains are analyzed with libraries of substrate-specific profile hidden Markov models in order to determine their putative substrates. However, in order to minimize the incidence of false positives to rare monomers, the presence of additional biosynthetic domains is required in order to identify methoxymalonate (acyl-CoA dehydrogenase and 3-hydroxyacyl-CoA dehydrogenase), capreomycidine (L-arginine hydroxylase and capreomycidine synthase), diaminopropionate (diaminopropionate synthase), 3-hydroxypipecolate (cyclodeaminase and hydroxylase), and 3-hydroxyanthranilate (tryptophan dioxygenase and aryl formamidase). BLAST databases are used to assign starter condensation, epimerization, heterocyclization, and decarboxylative ketosynthase functionalities to condensation and ketosynthase domains based on the highest-scoring database sequence.

#### Cluster identification

Once enzymatic domains have been identified, they are grouped into clusters by a greedy algorithm. Starting on any open reading frame associated with a bond-forming domain, this algorithm expands on either side of the domain by a window set by default to 20 000 base pairs. This process repeats iteratively until PRISM is no longer able to detect bond-forming domains within 20 000 base pairs of either end of the putative cluster. Putative thiotemplated clusters (i.e. nonribosomal peptide, type I polyketides, and hybrid nonribosomal peptide-polyketides) are retained if they contain at least one bond-forming (condensation or ketosynthase) domain and at least one substrate-activating (adenylation, acyl-adenylating, or acyltransferase) domain. Putative type II polyketide clusters are retained if they contain both a ketosynthase α and a chain length factor. PRISM is capable of identifying, but not predicting the products of, iterative type I polyketide clusters. Putative enediyne clusters are retained if they contain a C-terminal phosphopantetheinyl transferase domain and a 9- or 10-membered enediyne ketosynthase domain, while putative fungal iterative type I polyketide clusters are retained if they contain a product template domain and and a ketosynthase domain.

### Scaffold open reading frame permutations and non-canonical assembly lines

#### Open reading frame permutations

**Figure 3.**
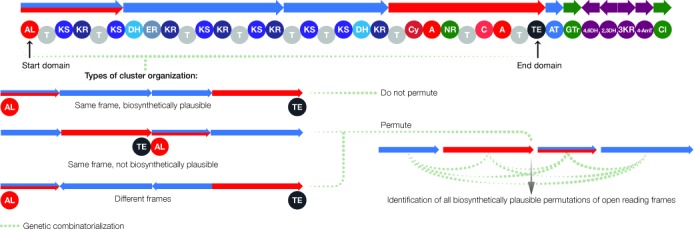
Scaffold open reading frame permutation in PRISM structure prediction. When scaffold open reading frames are in the same frame but biosynthesis in this frame is implausible, or scaffold open reading frames are not in the same frame, all biosynthetically plausible permutations of scaffold open reading frames are generated.

The principle of colinearity holds that, within biosynthetic gene clusters, groups of adjacent domains form operational modules responsible for the extension of the growing natural product scaffold (9). However, *trans-*acting adenylation and acyltransferase domains represent prominent examples of non-canonical natural product assembly lines. In order to account for these non-canonical biosynthetic systems, PRISM implements new algorithms to identify and predict *trans*-adenylation and *trans*-acyltransferase cluster products. Moreover, *de novo* chemical structure prediction software typically assumes a single permutation of scaffold open reading frames directing biosynthesis. PRISM implements additional logic to determine what subset of scaffold open reading frame permutations to consider in combinatorial scaffold library generation (Figure 3). Open reading frames with at least one module which end with a thioesterase domain are assumed to terminate biosynthesis. Likewise, open reading frames containing starter unit-adenylating ligases, starter condensation and decarboxylative ketosynthase domains are assumed to initiate biosynthesis. When all scaffold open reading frames within a cluster are in the same frame, only a single permutation is considered, but if one of these rules is violated, all biosynthetically plausible open reading frame permutations are generated. Likewise, when scaffold open reading frames are not all in the same frame, all biosynthetically plausible open reading frame permutations are considered. When the total number of permutations is greater than or equal to 7!, a pseudorandom sample of 500 biosynthetically plausible permutations is generated.

#### Module identification

PRISM identifies 10 types of modules, defined here as an enzymatic domain or set of domains responsible for the addition of a single monomer to the natural product scaffold. Adenylation modules are defined as the set of domains between a condensation domain and a thiolation or thioesterase domain, with an adenylation or acyl-adenylating domain in between. Adenylation modules lacking a substrate-activating domain are defined as potential sites of *trans*-adenylation didomain insertion. *Trans* or initiating adenylation modules are defined as the set of domains between an adenylation or acyl-adenylating domain and a thiolation domain, where the substrate-activating domain is the first domain identified on the parent open reading frame. Similarly, acyltransferase modules are defined as the set of domains between a ketosynthase domain and a thiolation or thioesterase domain, with an acyltransferase domain in between; acyltransferase modules lacking a substrate-activating domain are considered potential sites of *trans*-acyltransferase domain insertion. Potential *trans*-acting acyltransferase domains are identified when the number of domains on the parent open reading frame is equal to the number of acyltransferase domains. Starter condensation domains, which acylate the first amino acid (38), and their putative substrates are identified using BLAST databases compiled by Ziemert *et al*. (36) and expanded in this study. Acyl-adenylating ligases and prolyl-AMP ligases on their own open reading frames are also considered biosynthetically active modules.

#### Trans-acting acyltransferase domains

**Figure 4.**
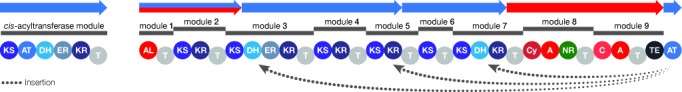
A recursive algorithm enables the identification of *trans*-acyltransferase modules split across two open reading frames in PRISM. Canonical *cis*-acyltransferase type I polyketide modules contain integrated acyltransferase domain for each module (left). In a hypothetical *trans*-acyltransferase polyketide cluster (right), modules lack integrated acyltransferase domains and may be split across open reading frames. A similar insertion process occurs when *trans*-acting adenylation domains are identified.

*Trans* -acyltransferase PKSs represent a large family of modular type I PKSs which lack integrated acyltransferase domains, and which evolved independently from the canonical *cis*-acyltransferase PKS architecture (39). Several challenges complicate the prediction of the structures of these complex natural products from genetic information. Potential sites of *trans*-acyltransferase domain insertion must be identified in order to generate a predicted scaffold. However, potential *trans*-acyltransferase insertion modules (defined here as the set of domains between ketosynthase and thiolation domains) are frequently split across multiple open reading frames (40–42). A recursive algorithm was therefore implemented within PRISM to construct artificial open reading frames when insertion modules spanning multiple open reading frames are identified; the artificial open reading frames are then used for scaffold generation (Figure 4). For *trans*-acyltransferase clusters in which scaffold open reading frames are not in the same frame, this process is repeated for each open reading frame permutation. In clusters where multiple *trans*-acting acyltransferase domains are detected, all are assumed to select malonyl-CoA.

#### Trans-acting adenylation domains

Another prominent exception to the principle of colinearity is the presence in several NRPSs (43–45) of adenylation-thiolation didomains which interact in *trans* with thiolation domains on different open reading frames. Potential *trans*-adenylation NRPSs are identified by the presence of an adenylation-thiolation didomain (here termed a *trans*-adenylation module) and a condensation-thiolation didomain (here termed a *trans*-adenylation insertion module). In order to account for both the possibilities of multiple insertion sites and of multiple *trans*-acting adenylation domains, all permutations of *trans*-adenylation modules are generated, and for each permutation, all combinations of insertion and *trans*-adenylation modules are generated. For each combination, a copy of the original insertion module-containing open reading frame is created, and the *trans*-adenylation module is inserted. All permutations of the resulting artificial open reading frames are subsequently generated and used to construct predicted scaffolds.

### Combinatorial scaffold library generation

#### Tailoring reactions

The large set of tailoring enzymes which modify linear natural product scaffolds is essential to both the biological activities and the structural diversity of natural products (46). PRISM therefore encodes 54 virtual reactions in order to maximize predictive accuracy. Several of these reactions are executed during the elaboration of the linear scaffold. The polyketide ‘reductive loop’ (including ketoreductase, dehydratase, and enoylreductase domains), for instance, modulates the oxidation states of ketide monomers, while C-, N- and O-methyltransferase domains methylate activated monomers. Heterocyclization condensation domains catalyze the cyclization of cysteine and serine residues to form thiazolines and oxazolines, respectively, which may subsequently be oxidized by nitroreductase domains to form thiazoles and oxazoles. However, the substrate of many tailoring reactions cannot be determined *a priori*: an uncharacterized glycosyltransferase, for instance, might react at any free hydroxyl group. Therefore, in addition to enumerating the biosynthetically plausible module permutations, a potential set of substrates for each tailoring reaction is identified for each module permutation, and all combinations of reaction substrates are generated.

Over 4500 halogenated natural products are known; within the polyketide and nonribosomal peptide families, the vast majority of halogenating enzymes incorporate chlorine (47). A phylogenetic analysis of natural product chlorinases revealed that these tailoring enzymes assort roughly according to their substrate (Supplementary Figure S1), enabling the development of a BLAST database of halogenases associated with the chlorination of tyrosine or phenylglycine, tryptophan, histidine, aromatic starter units, type II polyketides, threonine and brached-chain amino acids. A hidden Markov model for natural product chlorinases was developed, and identified chlorinating enzymes are analyzed with the BLAST database. BLAST results are sorted by score, and PRISM attempts to find substrates for each in turn until a set of potential substrates has been identified.

The biological activity of the glycopeptide antibiotics is associated with their unique conformation, which results from the oxidative cross-linking of aromatic amino acid residues by P450-dependent monooxygenases. Phylogenetic analysis by Hadatsch *et al*. (48) revealed that glycopeptide P450s implicated in the cyclization of the C-*O*-D, D-*O*-E, F-*O*-G, and AB rings occupy distinct clades. Identification of potential substrates for the glycopeptide P450s in PRISM is therefore position-specific. OxyA-type P450s, for example, are assumed to catalyze ether bridge formation between the *meta* carbons of tyrosine, β-hydroxytyrosine, or 4-hydroxyphenylglycine residues at position 2 and 4. Likewise, OxyC-type P450s are assumed to catalyze ether bridge formation between the *meta* carbon of a tyrosine, β-hydroxytyrosine, or 4-hydroxyphenylglycine residue at position 5 and the *ortho* carbon a 3,5-dihydroxyphenylglycine residue at position 7.

The β-lactam represents a privileged chemotype to which several biosynthetic routes have evolved convergently. We constructed new hidden Markov models to predict the structures of β-lactams generated by nonribosomal peptide synthetases, including models for the isopenicillin N synthase, the isopenicillin N acyltransferase, and the bifunctional deacetoxycephalosporin C synthase/hydroxylase. The isopenicillin N synthase and deacetoxycephalosporin C synthase/hydroxlase are assumed to react at predicted cysteine-valine diresidues, while the isopenicillin N acyltransferase is assumed to react at predicted 2-aminoadipic acid residues.

Several tailoring reactions are assumed to potentially react at any free hydroxyl group, including those catalyzed by glycosyltransferases, sulfotransferases, carbamoyltransferases and phosphotransferases. Other tailoring reactions are specific to certain predicted amino acid residues. Hidden Markov models were constructed for a tryptophan dioxygenase implicated in kynurenine formation in daptomycin and as an intermediate in quinoxaline biosynthesis, and for a proline dehydrogenase associated with pyrrole-containing natural products. A hidden Markov model was constructed for formyltransferases, which are assumed to react either at the N-terminus or the side chain nitrogen of ornithine and N-hydroxyornithine residues. Finally, a large set of type II polyketide-specific tailoring reactions and a combinatorial deoxysugar prediction algorithm were developed (see below). All tailoring reactions, and their associated hidden Markov models, are presented in Supplementary Dataset 1.

#### Macrocyclization

The conformational constraints imposed by macrocyclization, which occurs during chain release by thioesterase domains, are often essential to the biological activities of natural products (46). However, a recent phylogenetic analysis by Hari *et al*. (49) revealed that thioesterase domains do not cluster based on substrate specificity or function. Because it is apparently not possible to infer macrocyclization from the primary sequence of the thioesterase domain, PRISM considers all potential points of cyclization in combinatorial scaffold library generation, including serine and threonine residues, N-terminal amino acids, hydroxyl- or amine-containing starter units, β-hydroxylated amino acids and reduced ketide units, in addition to the linear carboxylic acid. The exception to this rule occurs when a C-terminal reductase domain, which catalyzes thioesterase-independent release, is located within the cluster, in which case PRISM generates only the linear aldehyde and cyclic imine.

#### Combinatorialization

**Figure 5.**
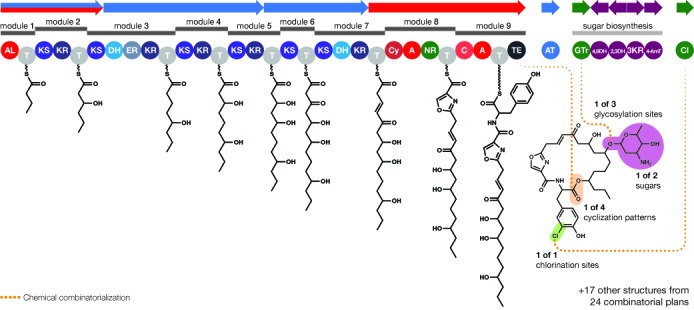
Combinatorial scaffold library generation for a hypothetical biosynthetic gene cluster in PRISM. Biosynthetic modules are identified and the linear scaffold is constructed based on the predicted substrate of the adenylation, acyl-adenylating or acyltransferase domain within each module. Potential deoxysugar combinations and sites of attachment, tailoring reaction substrates, and macrocyclization patterns are identified and combinatorialized. In this hypothetical cluster, two deoxysugars are predicted as potential substrates of the lone glycosyltransferase. The deoxysugar in each combinatorial plan can be added at any of three potential glycosylation sites. Three potential macrolactone cyclizations are identified in addition to the linear carboxylic acid, and a single chlorination site is predicted, producing a total of 24 combinatorial plans. Execution of each combinatorial plan produces a single predicted structure. Combinatorial plans which fail to execute (e.g. when macrocyclization and glycosylation take place at the same free hydroxyl) are discarded.

Scaffold generation is organized around the concept of a combinatorial plan, where each combinatorial plan represents the intersection of a single module permutation, macrocyclization pattern and a set of reaction plans; a reaction plan itself corresponds to the action of a single enzymatic tailoring reaction at a single potential substrate (Figure 5). Each open reading frame permutation is converted into a permutation of biosynthetic modules, and the basic linear scaffold is constructed by assembling the predicted residues associated with each biosynthetic module in the specified order. The set of reaction plans are executed on the substrates assigned to each reaction within the combinatorial plan, and the macrocyclization pattern is executed. A maximum of 1000 combinatorial plans is evaluated for each biosynthetic gene cluster.

Some elements of a given combinatorial plan may be mutually exclusive. Glycosylation and macrocyclization, for instance, cannot occur at the same hydroxyl group. Each scaffold library is therefore associated with a reaction count. Only newly generated scaffolds with a number of successfully executed reactions greater than or equal to the reaction count of the combinatorial scaffold library are retained. If the reaction count of a newly generated scaffold is greater than that of the combinatorial scaffold library, the current library is discarded. When the maximum size of the combinatorial library is less than the total size of combinatorial plans, a pseudorandom process generates a subset of all possible scaffolds by using an instance of the default Java random number generator with a seed of 0 to select combinatorial plans until either the maximum library size is reached, or all combinatorial plans have been executed.

### Informatic dereplication of known natural products

#### Genetic dereplication

**Figure 6.**
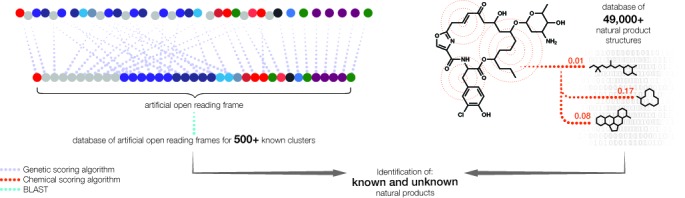
Genetic and chemical dereplication distinguishes known and unknown natural products in PRISM. Biosynthetic domains are placed in an arbitrary but consistent order and concatenated into a single artificial open reading frame, which is compared to artificial open reading frames generated for a database of 587 known clusters. Chemical graphs of predicted structures are decomposed into a one-dimensional bit set with the ECFP6 and FCFP6 algorithms and compared to a database of 49 680 known natural products.

One of the major goals of microbial genome analysis is to rapidly deduce the biosynthetic potential of an organism, and in particular whether biosynthetic gene clusters correspond to desired natural product classes or encode unknown metabolites. The rapid dereplication of known biosynthetic gene clusters is essential in order to direct research toward the discovery of new natural products. PRISM implements a multilocus sequence typing-inspired algorithm for biosynthetic gene cluster dereplication (50,51) (Figure 6). The algorithm is organized around the hypothesis that bond-forming sequence information is more conserved than non-bond-forming sequence information, and therefore leverages the ability of PRISM to identify biosynthetic domains. Bond-forming domains are sorted by type, with the result that all domains from the same family are grouped together. Bond-forming domains within the same family are subsequently sorted by their BLAST bitscore to a reference domain as a proxy for genetic distance. Domain protein sequences are concatenated into a single artificial open frame, which is aligned to a database of 587 nonribosomal peptide and polyketide gene clusters with BLAST. The total BLAST alignment score is divided by the number of residues aligned, and reported to the user. Homologous clusters in which the total coverage of the alignment does not represent at least 25% of the query artificial open reading frame are discarded. PRISM also reports a weighted average of the identity of biosynthetic open reading frames as determined by protein BLAST with preferential voting.

#### Cheminformatic dereplication

While homology to known clusters represents a rich source of information for dereplication, the number of natural products for which biosynthetic gene clusters have been experimentally verified and sequenced represents a small fraction of all known secondary metabolites. We therefore developed a graph database containing 49 860 natural products and associated information about producer organisms, biological activity, chemical fingerprints, literature references, physical properties and chemical archetypes (Rees *et al*., in preparation). This resource is used to cheminformatically dereplicate predicted natural product scaffolds. Chemical fingerprints, which decompose a chemical graph into a list of salient features, are widely used in assessing chemical similarity (52,53). 1024-bit fingerprints for all compounds in each cluster scaffold library are calculated using the RDKit implementation of the ECFP6 and FCFP6 algorithms (54). Each hypothetical structure in the scaffold library is subsequently compared to every natural product in the database by means of the widely used Tanimoto coefficient (55). Similarity coefficients between predicted structures and known compounds are sorted by ECFP6 coefficient, and the highest-scoring matches are presented to the user.

#### Output

PRISM generates rich graphical HTML pages in order to make its output accessible to users without specialized bioinformatics training. The results of a PRISM search are reported as a table of clusters identified within a user-submitted sequence, including the biosynthetic open reading frames of each cluster color-coded by putative function, the predicted product if known, and the biosynthetic family of the cluster. For input files consisting of a single contig, PRISM generates a map of the physical location of each cluster on a stylized bacterial chromosome. PRISM also generates more detailed output for each cluster on a separate HTML page, including the predicted biosynthetic assembly line, homologous clusters, similar molecules and their Tanimoto coefficients, predicted sugar combinations, the combinatorial structure library, and detailed analysis of each open reading frame. The set of combinatorial information evaluated in scaffold library generation, including the number of open reading frame permutations, cyclization patterns, sugar combinations, tailoring reaction plans, and the total number of combinatorial plans generated, is also presented in HTML format. Finally, PRISM output is also generated in JSON format in order to allow users to save PRISM results in a highly compressed format, and PRISM includes a servlet to generate HTML pages from saved JSON output under the ‘Open’ tab. Although SMILES output by PRISM can be rendered in most desktop chemical drawing applications, a simple web application capable of rendering SMILES in the browser using the ChemDoodle Web Components library (56) is available at http://magarveylab.ca/render-smiles/.

### Systematic analysis of extra-modular adenylation domains

Within a prototypical nonribosomal peptide synthetase, adenylation domains are incorporated in modules, which are in turn responsible for the addition of a single amino acid to the growing natural product scaffold. The adenylation domain, which confers substrate specificity (57,58), activates an amino acid as aminoacyl-AMP and transfers it to the phosphopantetheinyl group of the thiolation domain. The condensation domain then catalyzes amide bond formation. Exceptions to this modular architecture, however, abound within biosynthetic gene clusters and represent a major challenge to structure prediction. Examples of such exceptions include starter modules, which contain an adenylation domain but lack a condensation domain, and initiate biosynthesis (59,60). Modules which lack a catalytic condensation domain may also be activated in *trans* by a module on another open reading frame lacking an adenylation domain (45,61,62). Still other extra-modular adenylation domains are been implicated in nonproteinogenic amino acid biosynthesis (63–65) or starter unit (66,67) activation. Stand-alone adenylation domains (68) have been reported to both activate amino acids and catalyze amide bond formation. A large family of adenylate-forming domains activates fatty acids as acyl-CoA or acyl-AMP esters and transfers them to the natural product scaffold, often as a starter unit (69–71). A small number of aminoacylating modules that lack a thiolation domain (72,73) may be part of modules that are split across two open reading frames. Finally, some extra-modular adenylation domains (74–77) are biosynthetically inactive.

We sought to infer the biosynthetic function of extra-modular adenylation domains in order to maximize the predictive accuracy of PRISM, and therefore performed a systematic analysis of extra-modular adenylation domains. A data set of 199 adenylation domains not located within a prototypical condensation-adenylation-thiolation module was compiled by analyzing the PRISM biosynthetic gene cluster database. A literature review was then performed for each domain in order to assign its known or predicted function and chemical substrate (Supplementary Dataset 2). A phylogenetic tree was constructed from the primary sequences of the annotated adenylation domains. The complete adenylation tree, with all leaves labeled, is presented in Supplementary Figure S2, and a cladogram with bootstrap values is presented in Supplementary Figure S3.

The resulting phylogenetic tree of adenylation domains was observed to bifurcate, revealing two distinct clades of adenylation domains: conventional adenylation domains, which activate proteinogenic and nonproteinogenic α-amino acids, and a distinct group of acyl-adenylating enzymes responsible for activating non-α-amino acid substrates, including long- and short-chain fatty acids, aromatic starter units, and α-keto and α-hydroxy acids. Moreover, enzymes within this latter group were observed to assort based on their substrates. Within a larger clade of fatty acid-activating domains, for instance, identifiable groups of acyl-adenylating enzymes specific to long- or short-chain fatty acids were observed; likewise, within the aromatic starter unit-activating clade, there were identifiable groups of domains specific to 3-amino-5-hydroxybenzoic acid, 3-hydroxypicolinic acid, 2,3-dihydroxybenzoic acid, and 3-formamido-2-hydroxybenzoic acid. Conversely, within the clade of α-amino acid-activating adenylation domains, there was no clear pattern that would allow the assignment of *trans*-adenylation, initiating, or nonproteinogenic amino acid biosynthesis functionality on the basis of primary sequence alone. Instead, adenylation domains appeared to assort on the basis of their amino acid substrates. The clade of threonine adenylation domains highlighted in Supplementary Figure S2 exemplifies the manner in which extra-modular adenylation domains with distinct functionalities (including both *trans*-acting and initiating adenylation domains), and corresponding to diverse natural product chemotypes (including glycopeptides, lipopeptides, and monobactams), form a single clade.

On the basis of these results, we developed a new hidden Markov model for the distinct clade of non-α-amino acyl-adenylating enzymes, and integrated this family of enzymes into PRISM as a third class of substrate-activating domains. We additionally created a library of 26 profile hidden Markov models specific to acyl-adenylating enzymes substrates (see Supplementary Dataset 1) in order to predict the monomers or starter units activated by this family of enzymes. Salicylate and phenylacetate hidden Markov models developed by Khayatt *et al*. (12) were discarded. Two new hidden Markov models were created for the small clades of adenylation domains implicated in the biosynthesis of nonproteinogenic amino acid β-hydroxytyrosine within the glycopeptide family, and the tryptophan-derived starter units quinoxaline and 3-hydroxyquinoxaldic acid within the quinomycin family. Finally, a new adenylation domain hidden Markov model was constructed for the highlighted clade of prolyl-AMP ligases from pyrrole-containing natural products, as these were observed to be poorly predicted. We next developed new rules to assign function to adenylating domains within PRISM. While acyl-adenylating domains on their own open reading frame are assumed to be active, adenylation domains on their own open reading frame are not. However, adenylation-thiolation didomains are assumed to be biosynthetically active. When a biosynthetic gene cluster contains at least one C-T didomain, adenylation-thiolation didomains on their own open reading frames are assumed to be act in *trans*. Finally, C-A didomains which end an open reading frame are also assumed to be active. When a cluster contains more than one acyl-adenylating enzyme, only open reading frame permutations beginning with an acyl-adenylating enzyme are considered, and remaining acyl-adenylating enzymes are considered tailoring reactions.

In order to evaluate the impact of the addition of 15 new adenylation domain profile hidden Markov models and a parallel process for identifying acyl-adenylating ligases and their substrates, we benchmarked the expanded ensemble of hidden Markov models. The annotated data set of 498 adenylation domain sequences developed by Khayatt et al. (12) was obtained. Because several of the original profile hidden Markov models were associated with more than one substrate (e.g. isovaline/2-aminobutyric acid), adenylation domains with these substrates were removed from our analysis. Additionally, sequences which preferentially hit the newly developed acyl-adenylating ligase hidden Markov model, including phenylacetate and salicylate-activating adenylation domains, were discarded to produce a subset of 460 adenylation domain sequences. The expanded ensemble of profile hidden Markov models correctly identified the substrates of 445 of the 460 adenylation domains, or 96.7%, based on the highest-scoring profile hidden Markov model (Supplementary Dataset 3).

### Sugar prediction

Many natural products are elaborated with deoxysugar moieties which are essential to their biological activity (19). The biosynthesis of a diverse set of deoxysugar appendages proceeds from the combination of a limited set of enzyme activities. We therefore developed a library of hidden Markov models for deoxysugar biosynthesis genes, and a combinatorial strategy to enumerate potential glycosylation patterns based on genetic information.

#### Deoxysugar domain identification

We constructed a library of 19 hidden Markov models for TDP-sugar biosynthesis based on the glycogenomic code developed by Kersten *et al*. (78), and additionally developed new hidden Markov models for UDP-sugar dehydrogenases and decarboxylases implicated in biosynthesis of pentose deoxysugars in natural products such as calicheamicin and maduropeptin (79).

#### Combinatorial deoxysugar prediction

Predicting natural product glycosylation patterns from genetic information is inherently challenging for several reasons: natural products often contain multiple sugars, individual enzymes may be shared between multiple sugar biosynthesis pathways, individual pathways may not be physically segregated within the gene cluster, and natural products may be glycosylated with both deoxygenated and hexose sugars (80,81). We therefore developed a combinatorial strategy to evaluate potential glycosylation patterns based on consideration of whether the identified set of deoxysugar genes is maximally necessary and sufficient for any given combination of deoxysugars. The glycogenetic code described by Kersten *et al*. (78), which codifies the enzyme activities required for each deoxysugar biosynthesis pathway, was revised and expanded, and implemented for 63 deoxysugars. Hexose glycosyltransferases and their substrates are identified by BLAST analysis, and all remaining glycosyltransferases are considered to be deoxysugar-specific. The number of deoxysugar glycosyltransferases is assumed to correspond to the number of deoxysugars in the natural product, and all combinations with repetitions of that size (when *n* < 4) or a pseudorandom set of 2500 combinations (when *n* ≥ 4) are evaluated. All combinations which minimize both the number of genes present in the cluster but absent from the deoxysugar biosynthesis pathways, and the number of genes present in the deoxysugar biosynthesis pathways but absent from the cluster, are retained (up to a maximum of 100), and identified hexose sugars are added to each combination. All potential substrates for each glycosyltransferase domain, including other sugars in order to account for the possibility of oligosaccharide chains, are combinatorialized.

#### Hexose sugar domain identification

Several natural products contain both hexose and deoxysugars, but because hexose sugar biosynthesis is a function of primary metabolism, the associated genes are scattered throughout the genome. An alternative strategy was therefore required to predict the presence of hexose sugars on natural product scaffolds. A phylogenetic analysis of natural product glycosyltransferase domains (82) revealed three distinct clades of hexose-specific glycosyltransferases, as well as a single mixed clade corresponding to position-specific glycopeptide glycosyltransferases (83). On the basis of these results, an annotated BLAST database of natural product glycosyltransferases and their substrates was constructed, and is used within PRISM to identify hexose glycosyltransferases and their substrates (glucose, mannose, gulose, or N-acetylglucosamine) but not deoxysugars, which are predicted on the basis of the observed set of deoxysugar biosynthesis genes.

### Type II polyketide prediction

Type II polyketides represent a large and structurally diverse group of pharmaceutically important metabolites (84). In contrast to large, multi-modular type I PKSs, type II PKSs are complexes of monofunctional proteins which act iteratively to synthesize polyketide scaffolds. The minimal type II PKS consists of three proteins: a ketosynthase-chain length factor heterodimer catalyzes iterative condensations of malonyl-CoA extender units with an acyl starter unit, and an acyl carrier protein anchors the growing chain. The resulting reactive poly-β-ketothioester intermediate may be regiospecifically reduced by one or more ketoreductases before being directed toward a particular cyclization pattern by cyclases. Finally, the aromatic scaffold is modified by tailoring enzymes including glycosyltransferases, methyltransferases, and oxygenases in order to produce the final polyketide product.

Recently, interest has grown in the application of bioinformatics to type II polyketide discovery. Kim and Yi (85) compiled a database of 42 known, complete type II polyketide biosynthetic gene clusters and identified 40 putative type II polyketide clusters within publicly available actinobacterial genomes. Ogasawara *et al*. (86) developed Dynamite, a Python software package which uses a BLAST database to identify type I and type II PKS and NRPS gene clusters within the NCBI database, and conducted a global phylogenetic analysis of type II polyketide ketosynthases. However, at present, no software exists to predict the chemical structures of type II polyketides from genetic information. We therefore constructed a library of hidden Markov models for type II polyketide biosynthesis enzymes, and codified the biosynthetic logic which has been elucidated for type II polyketide biosynthetic gene clusters, in order to construct a generalizable *de novo* structure prediction algorithm for type II polyketides within PRISM.

#### Identification of the minimal PKS and prediction of polyketide chain length

Both the ketosynthase α (KSα) and chain length factor (CLF or KSβ) occupy distinct clades within ketosynthase phylogeny (87), and several studies (86,88,89) have reported that the length of the unreduced ketide chain produced by the minimal PKS can be reliably inferred from the primary sequence of the chain length factor. We therefore constructed new hidden Markov models for type II PKS KSα and CLF domains, and compiled a BLAST database of CLF domains annotated with the number of ketide chain extension cycles associated with the PKS. Annotated sequences were collected based on a global phylogenetic analysis of type II polyketide ketosynthases performed with the software platform Dynamite (86), and manually supplemented with domains from known PKSs observed to be absent from this data set. Within PRISM, the extension length of a type II PKS is inferred based on the single most homologous CLF, and used to generate a linear poly-β-ketide chain with a corresponding number of iterative decarboxylative condensations of malonyl-CoA with the identified starter unit.

#### Starter unit

Typically, biosynthesis of the polyketide chain by the minimal PKS is primed by an acetyl unit derived from the decarboxylation of malonyl-CoA, but type II polyketides such as doxorubicin, fredericamycin, and enterocin include alternative starter units. In the biosynthetic pathways of these compounds, the starter unit may be incorporated either by the condensation of the first malonyl-CoA extender unit with the starter unit by a ketosynthase III or via activation by an acyl-CoA ligase; in both cases, a priming acyltransferase transfers the resulting diketide or activated starter unit to the minimal PKS. A phylogenetic analysis performed by Novakova *et al*. (90) revealed that these priming acyltransferases assort with malonyl-CoA:ACP transacylases. We therefore developed hidden Markov models for priming acyltransferase and KSIII domains, and compiled a BLAST database of priming acyltransferases associated with seven starter units (propionate, butyrate, isobutyrate, 2-methylbutyrate, hexadienoate, benzoate, and malonamate). In order to reduce the incidence of false positives to non-alkyl substituents, we additionally constructed hidden Markov models for key enzymes implicated in the biosynthesis of malonamate, within the tetracycline family, and benzoate, within the enterocin and wailupemycin family. The presence of a phenylalanine ammonia-lyase or an amidotransferase is required in order to identify benzoate or malonamate, respectively, as the starter unit. In type II polyketide clusters that contain a priming acyltransferase, acyl-adenylating ligases are assumed to be inactive.

#### Cyclization

Fritzsche *et al*. (91) conducted a phylogenetic analysis of type II polyketide cyclases and revealed a strong correlation between primary sequence and cyclization pattern, including both regioselectivity and ring topology. We therefore constructed a library of twelve hidden Markov models for type II polyketide cyclases based on their results. Three clades were observed to be heterogeneous with respect to the cyclization pattern catalyzed by member cyclases. Clade V was split into two clades to discriminate between tetracenomycin fourth ring cyclases and pentangular polyphenol fourth and fifth ring cyclases. Clade VIII was split into two clades in order to identify benzoisochromanequinone first and second ring cyclases, and the remaining domains were merged with clade IX. For the final polyphyletic clade VIb, which includes both anthracyclines and tetracycline/aureolic acid fourth ring cyclases, the cyclization pattern is inferred within PRISM based on the CLF.

#### Ketoreductases

Both Zhang *et al*. (92) and Lackner *et al*. (93) have reported strong correlations between the regioselectivity and primary sequences of type II polyketide ketoreductases. We therefore constructed hidden Markov models for C9, C11, C15, C17, and C19 ketoreductases (all carbon numbering based on the linear poly-β-ketothioester). Because of the regioselectivity of these tailoring modifications, only a single site is considered within the combinatorial library generation engine.

#### C-glycosyltransferases

Type II polyketides including benzoisochromanequinones such as granaticin, anthracyclines such as nogalamycin and angucyclines such as urdamycin contain deoxysugar appendages attached to the aromatic scaffold via carbon-carbon bonds. Ichinose *et al*. (94) conducted a phylogenetic analysis of glycosyltransferases associated with type II polyketide biosynthetic gene clusters, and observed that C-glycosyltransferases occupied a distinct clade on the resulting phylogenetic tree. On the basis of these results, we constructed a hidden Markov model for C-glycosyltransferases and integrated this distinct class of glycosyltransferases into our previously reported system for combinatorial deoxysugar prediction (82). All aromatic α carbons with two bonds are considered possible sites of C-glycosylation.

#### Methyltransferases

Phylogenetic analysis by Ishida *et al*. (95) revealed that, despite the low degree of amino acid conservation between type II polyketide methyltransferases, there is an excellent correlation between methyltransferase sequence and the regioselectivity of the associated methyl transfer. Strikingly, methyltransferases with different functions clustered according to the site of methylation. For instance, the tetracycline C2 N,N-dimethyltransferase OxyT, from the oxytetracycline cluster, assorted with aureolic acid C2 O-methyltransferases MtmMI and CmmMI, from the mithramycin and chromomycin clusters, respectively. In contrast, two bis-C-methyltransferases from type II polyketide systems, RemG and BenF (from the resistomycin and benastatin clusters, respectively), did not assort together, apparently due to their distinct regioselectivities. We therefore constructed hidden Markov models for C6, C8 and C10 C-methyltransferases, carboxy-O-methyltransferases, C2 methyltransferases, and two distinct clades of C11 O-methyltransferases. As with type II polyketide ketoreductases, the strong correlation between primary sequence and regioselectivity allowed for the consideration of a single site of methyl transfer within the combinatorial library generation engine. The reaction associated with the C2 methyltransferase is assumed to be either N,N-dimethylation or O-methylation depending on whether nitrogen or oxygen is present at C2.

#### Oxygenases

Oxidative reactions are a major component of polyketide structural diversity. Oxygenases implicated in type II polyketide biosynthesis include anthrone-type oxygenases, cytochrome P450s, and flavin-dependent mono- and dioxygenases. Anthrone-type oxygenases catalyze quinone formation using molecular oxygen without any prosthetic groups, ions, or cofactors (96), and are therefore readily identifiable by their primary sequence. Flavin-dependent oxygenases catalyze a wide range of oxidative reactions, including hydroxylations, epoxidations, and Baeyer–Villiger and Favorskii rearrangements. However, phylogenetic analysis by Palmu *et al*. (97) of angucycline flavin-dependent monooxygenases revealed that only enzymes implicated in quinone formation occupy a distinct clade. Phylogenetic analysis by Zhang *et al*. (92) additionally revealed a poor correlation between the regioselectivity of Baeyer–Villiger monooxygenases and their primary sequences. Moreover, the rearrangement which results from the formation of the cyclic ester is effectively impossible to predict from the primary sequence of the associated enzyme. We therefore developed hidden Markov models for anthrone-type oxygenases, angucycline quinone-forming oxygenases, aureolic acid Baeyer–Villiger monooxygenases and the enterocin favorskiiase. However, predicting the function of other type II polyketide oxygenases from primary sequence data remains a major challenge.

#### Other tailoring reactions

Finally, we constructed a hidden Markov model for the C2 aminotransferase found within the tetracycline family of type II polyketides.

### Identification of resistance determinant domains

Antibiotic resistance is a major global problem which has highlighted the need for new antimicrobial agents with new mechanisms of action. Recently, we presented a method to prioritize biosynthetic gene clusters for natural product discovery based on their mechanism of action (Johnston *et al*., submitted). The collection of known resistance determinants, or resistome, within an organism's genome or associated with a particular biosynthetic gene cluster can be identified within PRISM, and identified resistance determinants used to make inferences about the biological target or mechanism of action of a genetically encoded metabolite. The Resfams database of resistance determinants (37) was downloaded and split into discrete hidden Markov model files. Several hidden Markov models were removed as they were observed not to be specific to antibiotic resistance (i.e. false positive hits were observed within genes implicated in natural product biosynthesis). Bitscore cutoffs were also modified for several models. We supplemented the resulting set of 164 Resfams hidden Markov models with 100 new hidden Markov models (see Supplementary Dataset 1). Resistance genes within the cluster window are automatically detected during the clustering process, but will not cause the greedy algorithm to expand the cluster.

## RESULTS

We compared the accuracy of the PRISM structure prediction engine to existing genetic natural product structure prediction software (see Supplemental Methods). Data sets of biosynthetic gene clusters corresponding to seven clinically relevant classes of natural products (type II polyketides, macrolides, trans-acyltransferase polyketides, cyclic/branched peptides, lipopeptides, glycopeptides, and β-lactams) were compiled, and predicted structures generated by PRISM, NP.searcher and antiSMASH 3.0 were compared to the structures of the corresponding natural products by means of the widely used Tanimoto coefficient (52,55,98–101), with chemical fingerprints generated using the ECFP6 algorithm (54). Because both PRISM and NP.searcher output combinatorial libraries of predicted structures, only the median Tanimoto coefficient was considered, while only consensus SMILES output by antiSMASH 3.0 were considered.

**Figure 7.**
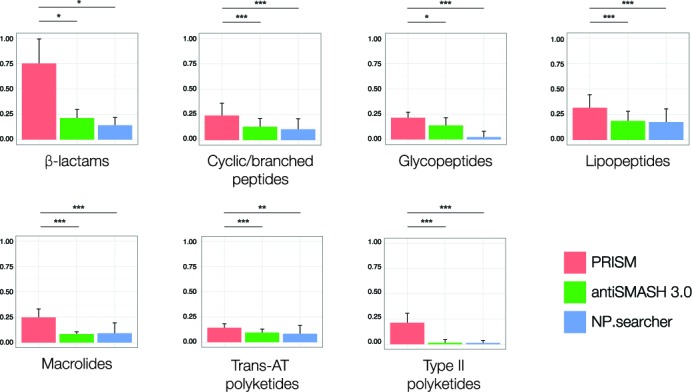
Comparison of PRISM, NP.searcher and antiSMASH 3.0 structure predictions across seven biosynthetic classes of natural products, as quantified by the Tanimoto coefficient. Sample sizes are as follows: beta-lactams, *n* = 3; cyclic/branched peptides, *n* = 25; glycopeptides, *n* = 12; lipopeptides, *n* = 31; macrolides, *n* = 28; trans-acyltransferase polyketides, *n* = 22; type II polyketides, *n* = 46. Biosynthetic gene clusters, in FASTA format, and all predicted structures, in SMILES format, are available at http://magarveylab.ca/Skinnider_etal/accuracy/. *P* < 0.05, **P < 0.01, ***P < 0.001 (two-tailed Student's *t*-test).

Our results (Figure 7) demonstrate conclusively that PRISM is superior to existing methods for the genomic prediction of microbial secondary metabolomes across a wide range of biosynthetic classes. The improved predictive accuracy of PRISM with respect to β-lactams and glycopeptides (*P* < 0.05) was not surprising, as neither NP.searcher nor antiSMASH 3.0 are designed to identify the unique oxygenases implicated in the formation of the characteristic scaffolds of these families of natural products. However, we were surprised to observe a significant (*P* < 0.001) improvement in PRISM structure predictions for cyclic/branched peptides, as existing methods rely principally on the accurate prediction of amino acid and ketide monomers. The differential predictive accuracy of PRISM and existing methods is likely multifactorial. Khayatt *et al*. (12) reported that their ensemble of profile hidden Markov models enabled significantly more accurate substrate prediction than methods predicated on the identification of conserved active site residues as a specificity-conferring code (57), to the extent that their hidden Markov models outperformed NRPSsp (11) on its own training data set. PRISM supplements this library with 15 new substrate profile hidden Markov models and prerequisite domains for biosynthetic pathway-based rare substrate prediction, whereas both NP.searcher and antiSMASH 3.0 rely on the identification of conserved active site residues to predict substrate specificity. The ability of PRISM to additionally identify α-keto acid-activating domains and aromatic starter units likely contributed to more accurate prediction of depsipeptides (such as valinomycin and cereulide) and aromatic starter unit-containing compounds (such as the quinomycin-type antibiotics and cyanobacterial phenylacetate-containing metabolites).

PRISM generated significantly more accurate predicted structures than existing methods for lipopeptides (*P* < 0.001) and macrolides (*P* < 0.001), two classes of natural products which contain relatively few tailoring reactions. For lipopeptides, these results appear to validate the methods used within PRISM to identify starter units. While macrolides are less frequently associated with unusual starter units, they are by definition macrocyclized and are frequently glycosylated by one or more specialized deoxysugars. Our results suggest that the macrocyclization, deoxysugar prediction and starter unit identification functionalities within the PRISM structure prediction engine facilitated accurate prediction within the lipopeptide and macrolide classes of natural products.

It is not surprising that PRISM would be considerably more accurate than antiSMASH 3.0 or NP.searcher with respect to predicting the structures of type II polyketides (*P* < 0.001), as neither program is designed to predict this family of natural products. We were, however, surprised to observe that PRISM and antiSMASH 3.0 structure predictions for *trans-*acyltransferase polyketides were only slightly more accurate than those generated by NP.searcher, which is not designed to predict chemical structures from biosynthetic gene clusters of this family, and consequently generates small structures derived solely from the substrates of the acyltransferase and adenylation domains in each cluster. This may be due to the unique set of tailoring reactions reported for *trans*-acyltransferase polyketides, and consequently the inability of PRISM to predict unique structural features such as the leinamycin 1-oxo-1,2-dithiolan-3-one or the difficidin phosphate group which contribute significantly to local atom environments. Additionally, even with the implementation of a recursive algorithm to identify modules split across multiple open reading frames, predicting the oxidation state of ketide units in *trans*-acyltransferase polyketides remains challenging as ketoreductase, dehydratase or enoylreductase activities may be provided in *trans*, as in the biosynthetic gene clusters for difficidin, pederin, macrolactin, myxovirescin, leinamycin, and thailandamide (102). Clearly, accurately predicting the structures of *trans*-acyltransferase polyketides from genetic information remains challenging.

In order to explore the combinatorial information evaluated in scaffold library generation, and its effect on predictive accuracy, we calculated the number of open reading frame permutations, sugar combinations, cyclization patterns, tailoring reaction plans, and total combinatorial plans evaluated for each cluster within the test data set (Supplementary Dataset 4). Only two of 163 clusters within this data set (1.2%) exceeded the limit of 500 open reading frame permutations. For the vast majority (116 of 163, or 71.1%), only a single permutation was evaluated, while for 145 (90.0%), 10 or fewer permutations were evaluated. However, while 38 of the 50 glycosylated clusters (76.0%) in the test data set generated 10 or fewer sugar combinations, six of the 50 (12.0%) generated 100 or more. All clusters with 100 or more sugar combinations were type II polyketides with redundant or inactive deoxysugar glycosyltransferases, causing the algorithm to attempt to match the observed set of deoxysugar biosynthesis genes to an incorrect number of sugars. For instance, in the saquayamycin cluster, six glycosyltransferases catalyze the attachment of nine deoxysugar moieties to the aglycone (103), while the SnogZ glycosyltransferase of the nogalamycin gene cluster is redundant (104). It is possible that future work may establish a rationale for a sugar prediction engine that does not rely on an initial prediction of the number of deoxysugars within a natural product, which would improve the performance of PRISM in these cases.

While all 163 clusters generated 10 or fewer potential cyclization patterns, only 120 (73.6%) generated 10 or fewer tailoring reaction plans, while 18 (11.0%) generated 500 or more. These included the two clusters exceeding the limit of 500 open reading frame permutations, since reaction plans are calculated for each permutation, and the six compounds with 100 or more sugars, since multiple attachment sites are possible for each sugar in each sugar combination, in addition to eight clusters (4.9% of the data set) where the sheer number of combinations of tailoring reaction domains and their substrates was the only factor contributing to the number of tailoring reaction plans evaluated. These data suggest that, although tailoring reactions are essential to the structural diversity and biological activities of natural products, in a small minority of cases it is difficult to accurately predict their substrates *a priori*.

For several clusters, a large set of tailoring reaction plans was evaluated, but the resulting scaffold library was relatively small. For instance, 2,058 tailoring reaction plans were evaluated for the A40926 cluster, but the resulting combinatorial library contained only 75 scaffolds. This phenomenon can be attributed to the design of the PRISM combinatorialization engine, which detects substrates for each tailoring enzyme independently. Thus the hydroxylated amino acids which are oxidatively cross-linked to form the glycopeptide scaffold are also considered possible sites of glycosylation. These data suggest that improvements to the design of the combinatorial scaffold generation engine may result in a small performance improvement. However, despite the large volume of combinatorial information evaluated, only 23 of the 163 clusters in the test data set (14.1%) produced combinatorial libraries containing 50 or more scaffolds, the default maximum library size.

We investigated the Tanimoto coefficients of predicted structures within the test data set, and plotted the distribution of Tanimoto coefficients for all 23 libraries containing 50 or more scaffolds (Supplementary Dataset 5). Although the Tanimoto coefficient is the *de facto* standard for quantifying structural similarity, it may underestimate the accuracy of PRISM predictions. For instance, within the teicoplanin cluster, all amino acid residues, sugars and the C10 acyl chain were correctly predicted, and all tailoring enzymes (three glycosyltransferases, four P450s and a halogenase) correctly identified. The top-scoring predicted structure differs from the true structure in two respects. A single chlorination event was associated with the iteratively chlorinating ORF10, due to the challenge inherent in identifying iteratively acting or nonfunctional enzymes. The C10 acyl chain was incorrectly placed at the N-terminus as a starter residue, as combinatorialization does not account for the relatively rare sugar acylation which occurs in teicoplanin biosynthesis. However, despite these comparatively minor errors in structure prediction, the median Tanimoto coefficient was 0.278, while the top Tanimoto coefficient was 0.293. The teicoplanin case study illustrates the limitations of two-dimensional chemical fingerprints, and the Tanimoto coefficient more generally, in quantifying structural similarity, particularly in the context of the unique and complex structures of natural products. Clearly, the Tanimoto coefficient is not an optimal metric of chemical similarity within the context of natural products, because this metric does not consider the biosynthetic origins of real or predicted structures.

**Figure 8.**
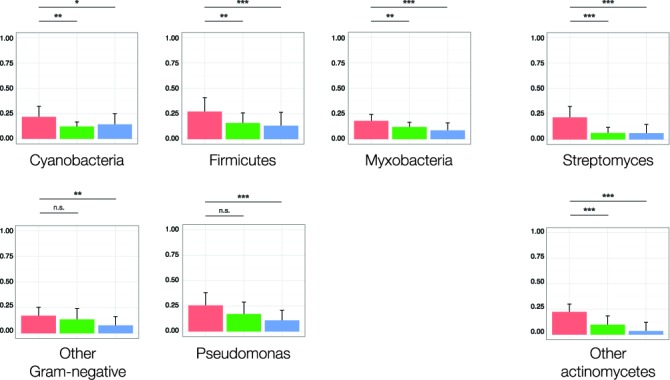
Comparison of PRISM, NP.searcher and antiSMASH 3.0 structure predictions across eight microbial natural product producer phylotypes, as quantified by the Tanimoto coefficient. Sample sizes are as follows: cyanobacteria, *n* = 18; firmicutes, *n* = 25; myxobacteria, *n* = 19; pseudomonads, *n* = 17; other Gram-negative bacteria, including Xenorhabdus, Burkholderia, Vibrio and Serratia, *n* = 16; streptomycetes, *n* = 89; other actinomycetes, *n* = 17. Biosynthetic gene clusters, in FASTA format, and all predicted structures, in SMILES format, are available at http://magarveylab.ca/Skinnider_etal/phylo/. *P < 0.05, **P < 0.01, ***P < 0.001, n.s. not significant (two-tailed Student's *t*-test).

Natural products discovery efforts have traditionally privileged a small subset of talented secondary metabolite producers, such as fungi and terrestrial actinomycetes (105). Within this latter group, the genus *Streptomyces* alone accounts for over 80% of known secondary metabolites (106). However, in recent years, genome sequencing has revealed many nonribosomal peptide and polyketide clusters in other organisms (107), and consequently increased attention has been paid in recent years to nontraditional sources such as cyanobacteria and mycobacteria (2). In order to assess the ability of PRISM to predict the structures of secondary metabolites across diverse classes of organisms, we conducted a comparative analysis of predictive accuracy across seven microbial phylotypes, including Cyanobacteria, Firmicutes, Myxobacteria, Pseudomonads, and other Gram-negative bacteria such as Burkholderia and Xenorhabdus (Figure 8). Our results indicate a large increase in predictive accuracy for streptomycetes and other actinomycetes. However, PRISM also improved structure prediction relative to NP.searcher across all five underprivileged producer phylotypes, and across three relative to antiSMASH 3.0.

We finally sought to compare the performance of the multilocus sequence typing-style algorithm implemented within PRISM for biosynthetic gene cluster dereplication to the KnownClusterBlast module included in antiSMASH 3.0 (16). A data set of 33 nonribosomal peptide and polyketide biosynthetic gene clusters encoding known compounds in non-canonical producers was obtained from the ClusterMine360 database (108) (Supplementary Dataset 6), and was run through both antiSMASH 3.0 and PRISM (see also Supplementary Methods). PRISM correctly dereplicated 31 of the 33 clusters, while antiSMASH 3.0 correctly identified 26, suggesting an improvement in homology-based dereplication. In particular, PRISM correctly dereplicated all six fungal iterative type I polyketides missed by antiSMASH 3.0, suggesting that its MLST-style approach may be more appropriate for these small biosynthetic gene clusters.

## DISCUSSION

In the context of high rates of natural product rediscovery and the challenges associated with traditional natural product isolation programs, attempts have been made to harness a growing resource of genetic sequence data toward natural product discovery. In order to connect genomes to natural products, there is a need for accurate predictive algorithms capable of genomically profiling the biosynthetic capacity of microbes to produce variants of noted natural product families, as well as guiding chemists toward the isolation of completely new natural product chemotypes based on genetic data. However, the inherent variability of biological systems makes the accurate genetic structure prediction of complex natural products a challenging endeavour.

PRISM includes a wide range of functionalities in order to connect genes to molecules with unprecedented accuracy. PRISM implements a library of profile hidden Markov models to facilitate more accurate monomer prediction than existing methods, which rely on the identification of a substrate specificity-conferring code. PRISM additionally integrates a third family of substrate-activating domains, a large clade of acyl-adenylating enzymes revealed by phylogenetic analysis, including domains responsible for activating fatty acids, aromatic and alicyclic starter units, and α-keto and α-hydroxy acids. Specialized deoxysugar appendages, which are often directly responsible for the biological activities of natural products, are predicted using a combinatorial strategy. A diverse library of 54 tailoring reactions, particularly those associated with industrially relevant chemotypes, is implemented in order to predict the chemistries catalyzed by chlorinases, C- and O-glycosyltranferases, flavin-dependent and P450 oxygenases, oxidoreductases, methyltransferases, carbamoyltransferases, formyltransferases, phosphotransferases, and many other enzymes. PRISM additionally expands the subset of natural product chemical space that can be accessed by genomic predictions to type II polyketides, a large and clinically relevant family of compounds. Analysis of our database of known biosynthetic gene clusters revealed that approximately 84% of clusters contained at least one of the tailoring reactions encoded by PRISM (Supplementary Dataset 7). This diverse set of novel operations therefore significantly increases the breadth of chemical material predicted by PRISM relative to existing methods, and consequently the accuracy of PRISM structure predictions. Although PRISM implements a comprehensive library of hidden Markov models, BLAST databases, and virtual reactions to address the prediction of nonribosomal peptides and type I and II polyketides, it is also limited to these biosynthetic classes, which can be reliably predicted using rules established only after decades of rigorous study. Emergent rules for the prediction of alkaloids, terpenoids, ribosomally synthesized and post-translationally modified natural products (RiPPs), and other natural products may enable accurate and automated structure predictions in the future.

The principle of colinearity holds that, within biosynthetic gene clusters, adjacent domains can be grouped into modules responsible for the extension of the growing natural product scaffold by a single monomer unit (9). Typically, structure prediction software additionally assumes a single permutation for scaffold open reading frames, albeit with select exceptions: antiSMASH uses PKS docking domain sequence residue matching to predict the biosynthetic order of type I modular PKSs (109), while Pep2Path uses a Bayesian algorithm to infer module order within nonribosomal peptides from an annotated mass spectrum (110). However, existing methods generally do not account for the possibility of *trans*-acting adenylation or acyltransferase domains within the context of *de novo* structure prediction. PRISM codifies biosynthetic logic in order to account for several prominent exceptions to the principle of colinearity, including *trans*-acting adenylation and acyltransferase domains, and considers all biosynthetically plausible permutations of scaffold open reading frames. A comparison of all features in PRISM, antiSMASH 3.0 and NP.searcher is provided in Supplementary Table S1.

PRISM expands on the concept of combinatorial structure prediction introduced by Li *et al*. (15) in order to account for the uncertainty inherent in biological systems and generate maximally accurate structure predictions. Recent work by ourselves (82) and Zhang *et al*. (111) has demonstrated the utility of manually enumerating multiple hypothetical structures based on inherently low-fidelity predictions in the context of mass spectral analysis of microbial extracts. By automating this process, PRISM may enable researchers to rapidly connect genomes to metabolomes. Moreover, dereplication against the almost 50 000 known natural products leverages the PRISM combinatorial scaffold library generation engine in order to compare genetically identified material to a much broader area of chemical space than that represented by sequenced, annotated, and publicly available biosynthetic gene clusters. As a rapid and efficient platform suitable for high-throughput genomic analysis, and a user-friendly and graphically intuitive web application accessible to non-specialists, PRISM represents a valuable resource for the prediction of the genetically encoded secondary metabolomes of microbial organisms.

## Supplementary Material

SUPPLEMENTARY DATA
