# Supplementary material for: Genomes to natural products PRediction Informatics for Secondary Metabolomes (PRISM)
Source: Nucleic Acids Res. 2015 Oct 5;43(20):9645–62. doi: 10.1093/nar/gkv1012 (PMC4787774; doi:10.1093/nar/gkv1012)
Supplement: SUPPLEMENTARY DATA [file supp_43_20_9645__index.html]

Genomes to natural products PRediction Informatics for Secondary Metabolomes (PRISM) — Genomes to natural products PRediction Informatics for Secondary Metabolomes (PRISM) — SUPPLEMENTARY DATA 

# Genomes to natural products PRediction Informatics for Secondary Metabolomes (PRISM)

## SUPPLEMENTARY DATA

- SUPPLEMENTARY DATA
- SUPPLEMENTARY DATA
- SUPPLEMENTARY DATA
- SUPPLEMENTARY DATA
- SUPPLEMENTARY DATA
- SUPPLEMENTARY DATA
- SUPPLEMENTARY DATA
- SUPPLEMENTARY DATA
- SUPPLEMENTARY DATA
- SUPPLEMENTARY DATA
- SUPPLEMENTARY DATA
- SUPPLEMENTARY DATA
- SUPPLEMENTARY DATA
