# Supplementary material for: Genomes to natural products PRediction Informatics for Secondary Metabolomes (PRISM)
Source: Nucleic Acids Res. 2015 Oct 5;43(20):9645–62. doi: 10.1093/nar/gkv1012 (PMC4787774; doi:10.1093/nar/gkv1012)
Supplement: SUPPLEMENTARY DATA [file supp_gkv1012_nar-01872-z-2015-File010.docx]

Supplementary Figure 1. Phylogenetic analysis of nonribosomal peptide and polyketide chlorinases. A phylogenetic tree of 30 chlorinases reveals these tailoring enzymes assort roughly based on their substrates. The residue within the natural product scaffold chlorinated by each enzyme is denoted by branch colour. Bootstrap values are indicated for each branch.

Supplementary Figure 2. Phylogenetic analysis of extra-modular adenylation domains. A phylogenetic tree of 199 extra-modular adenylation domains reveals that while primary sequence phylogeny does not distinguish between standalone, inactive, or *trans*-acting adenylation domains, acyl- and aryl-adenylating ligases form a distinct clade from other adenylation domains and assort based on substrate. Tip labels indicate the identify of each domain, its substrate, and the associated natural product.

Supplementary Figure 3. Cladogram of extra-modular adenylation domain phylogenetic tree in Supplementary Figure 2 including bootstrap values.
