# Supplementary material for: Genomes to natural products PRediction Informatics for Secondary Metabolomes (PRISM)
Source: Nucleic Acids Res. 2015 Oct 5;43(20):9645–62. doi: 10.1093/nar/gkv1012 (PMC4787774; doi:10.1093/nar/gkv1012)
Supplement: SUPPLEMENTARY DATA [file supp_gkv1012_nar-01872-z-2015-File011.docx]

**SUPPLEMENTARY METHODS**

*Compilation of biosynthetic gene cluster datasets.* Libraries of complete and experimentally verified biosynthetic gene clusters for nonribosomal peptide β-lactams (n = 3), cyclic/branched peptides (n = 25), glycopeptides (n = 12), lipopeptides (n = 31), macrolides (n = 28), trans-acyltransferase polyketides (n = 22), and type II polyketides (n = 46) were manually compiled, and are available online at http://magarveylab.ca/Skinnider_etal/accuracy/. For phylogenetic predictive accuracy analysis, these datasets were reorganized and expanded in order to create new datasets for cyanobacteria (n = 18), firmicutes (n = 25), fungi (n = 12), myxobacteria (n = 19), pseudomonads (n = 17), and other Gram-negative bacteria, including Xenorhabdus, Burkholderia, Vibrio, and Serratia (n = 16). The sizes of the streptomyces and other actinomycetes datasets were 89 and 17, respectively. Phylogenetic datasets are available online at http://magarveylab.ca/Skinnider_etal/phylo/.

*Generation of predicted structures.* antiSMASH (version 3.0.2) ADDIN EN.CITE <EndNote><Cite><Author>Weber, T.; Blin, K.; Duddela, S.; Krug, D.; Kim, H. U.; Bruccoleri, R.; Lee, S. Y.; Fischbach, M. A.; Muller, R.; Wohlleben, W.; Breitling, R.; Takano, E.; Medema, M. H.</Author><Year>2015</Year><RecNum>180</RecNum><DisplayText>(1)</DisplayText><record><database name="My EndNote Library.enl" path="/Users/michaelskinnider/Documents/My EndNote Library.enl">My EndNote Library.enl</database><source-app name="EndNote" version="17.0">EndNote</source-app><rec-number>180</rec-number><foreign-keys><key app="EN" db-id="paazarvpaa559nes92r502wwvf52wxzszxxx">180</key></foreign-keys><ref-type name="Journal Article">17</ref-type><contributors><authors><author><style face="normal" font="default" size="100%">Weber, T.</style></author><author><style face="normal" font="default" size="100%">Blin, K.</style></author><author><style face="normal" font="default" size="100%">Duddela, S.</style></author><author><style face="normal" font="default" size="100%">Krug, D.</style></author><author><style face="normal" font="default" size="100%">Kim, H. U.</style></author><author><style face="normal" font="default" size="100%">Bruccoleri, R.</style></author><author><style face="normal" font="default" size="100%">Lee, S. Y.</style></author><author><style face="normal" font="default" size="100%">Fischbach, M. A.</style></author><author><style face="normal" font="default" size="100%">Muller, R.</style></author><author><style face="normal" font="default" size="100%">Wohlleben, W.</style></author><author><style face="normal" font="default" size="100%">Breitling, R.</style></author><author><style face="normal" font="default" size="100%">Takano, E.</style></author><author><style face="normal" font="default" size="100%">Medema, M. H.</style></author></authors></contributors><auth-address><style face="normal" font="default" size="100%">Novo Nordisk Foundation Center for Biosustainability, Technical University of Denmark, Horsholm, Denmark marnix.medema@wur.nl.Novo Nordisk Foundation Center for Biosustainability, Technical University of Denmark, Horsholm, Denmark.Department of Microbial Natural Products, Helmholtz Institute for Pharmaceutical Research Saarland, Helmholtz Centre for Infection Research, Saarland University, Saarbrucken, Germany.Department of Microbial Natural Products, Helmholtz Institute for Pharmaceutical Research Saarland, Helmholtz Centre for Infection Research, Saarland University, Saarbrucken, Germany German Centre for Infection Research (DZIF), Location Hannover-Braunschweig, Germany.Novo Nordisk Foundation Center for Biosustainability, Technical University of Denmark, Horsholm, Denmark Department of Chemical and Biomolecular Engineering (BK21 Plus Program) / BioInformatics Research Center, Korea Advanced Institute of Science and Technology, Daejeon, Republic of Korea.Congenomics, LLC, Glastonbury, CT, USA.Department of Bioengineering and Therapeutic Sciences/California Institute for Quantitative Biosciences, University of California, San Francisco, USA.Interfaculty Institute for Microbiology and Infection Medicine, Eberhard Karls University of Tubingen, Tubingen, Germany German Center for Infection Research (DZIF), Location Tubingen, Germany.Manchester Centre for Synthetic Biology of Fine and Speciality Chemicals (SYNBIOCHEM), Manchester Institute of Biotechnology, Faculty of Life Sciences, The University of Manchester, Manchester, UK.Microbial Genomics and Bioinformatics Research Group, Max Planck Institute for Marine Microbiology, Bremen, Germany Bioinformatics Group, Wageningen University, Wageningen, The Netherlands marnix.medema@wur.nl.</style></auth-address><titles><title><style face="normal" font="default" size="100%">antiSMASH 3.0-a comprehensive resource for the genome mining of biosynthetic gene clusters</style></title><secondary-title><style face="normal" font="default" size="100%">Nucleic Acids Res</style></secondary-title><alt-title><style face="normal" font="default" size="100%">Nucleic acids research</style></alt-title></titles><periodical><full-title><style face="normal" font="default" size="100%">Nucleic Acids Res</style></full-title><abbr-1><style face="normal" font="default" size="100%">Nucleic acids research</style></abbr-1></periodical><alt-periodical><full-title><style face="normal" font="default" size="100%">Nucleic Acids Res</style></full-title><abbr-1><style face="normal" font="default" size="100%">Nucleic acids research</style></abbr-1></alt-periodical><dates><year><style face="normal" font="default" size="100%">2015</style></year><pub-dates><date><style face="normal" font="default" size="100%">May 6</style></date></pub-dates></dates><isbn><style face="normal" font="default" size="100%">1362-4962 (Electronic)0305-1048 (Linking)</style></isbn><accession-num><style face="normal" font="default" size="100%">25948579</style></accession-num><abstract><style face="normal" font="default" size="100%">Microbial secondary metabolism constitutes a rich source of antibiotics, chemotherapeutics, insecticides and other high-value chemicals. Genome mining of gene clusters that encode the biosynthetic pathways for these metabolites has become a key methodology for novel compound discovery. In 2011, we introduced antiSMASH, a web server and stand-alone tool for the automatic genomic identification and analysis of biosynthetic gene clusters, available at http://antismash.secondarymetabolites.org. Here, we present version 3.0 of antiSMASH, which has undergone major improvements. A full integration of the recently published ClusterFinder algorithm now allows using this probabilistic algorithm to detect putative gene clusters of unknown types. Also, a new dereplication variant of the ClusterBlast module now identifies similarities of identified clusters to any of 1172 clusters with known end products. At the enzyme level, active sites of key biosynthetic enzymes are now pinpointed through a curated pattern-matching procedure and Enzyme Commission numbers are assigned to functionally classify all enzyme-coding genes. Additionally, chemical structure prediction has been improved by incorporating polyketide reduction states. Finally, in order for users to be able to organize and analyze multiple antiSMASH outputs in a private setting, a new XML output module allows offline editing of antiSMASH annotations within the Geneious software.</style></abstract><notes><style face="normal" font="default" size="100%">Weber, TilmannBlin, KaiDuddela, SrikanthKrug, DanielKim, Hyun UkBruccoleri, RobertLee, Sang YupFischbach, Michael AMuller, RolfWohlleben, WolfgangBreitling, RainerTakano, ErikoMedema, Marnix HENG2015/05/08 06:00Nucleic Acids Res. 2015 May 6. pii: gkv437.</style></notes><urls><related-urls><url><style face="normal" font="default" size="100%">http://www.ncbi.nlm.nih.gov/pubmed/25948579</style></url></related-urls></urls><electronic-resource-num><style face="normal" font="default" size="100%">10.1093/nar/gkv437</style></electronic-resource-num></record></Cite></EndNote>(1) source code was obtained from https://bitbucket.org/antismash/antismash/ and installed on a Linux Ubuntu 12.04 server running Python 2.7.3. argparse version 1.1, Straight Plugin version 1.4.0, cssselect version 0.9.1, pyquery version 1.2.9, numpy version 1.6.1, biopython version 1.65 ADDIN EN.CITE <EndNote><Cite><Author>Cock, P. J.; Antao, T.; Chang, J. T.; Chapman, B. A.; Cox, C. J.; Dalke, A.; Friedberg, I.; Hamelryck, T.; Kauff, F.; Wilczynski, B.; de Hoon, M. J.</Author><Year>2009</Year><RecNum>9</RecNum><DisplayText>(2)</DisplayText><record><database name="en00b035d3" path="/Users/michaelskinnider/Library/Caches/TemporaryItems/en00b035d3">en00b035d3</database><source-app name="EndNote" version="17.0">EndNote</source-app><rec-number>9</rec-number><foreign-keys><key app="EN" db-id="d5tvrwzw9wstsseszr6x2xfxvrx2wa5d5swx">9</key></foreign-keys><ref-type name="Journal Article">17</ref-type><contributors><authors><author><style face="normal" font="default" size="100%">Cock, P. J.</style></author><author><style face="normal" font="default" size="100%">Antao, T.</style></author><author><style face="normal" font="default" size="100%">Chang, J. T.</style></author><author><style face="normal" font="default" size="100%">Chapman, B. A.</style></author><author><style face="normal" font="default" size="100%">Cox, C. J.</style></author><author><style face="normal" font="default" size="100%">Dalke, A.</style></author><author><style face="normal" font="default" size="100%">Friedberg, I.</style></author><author><style face="normal" font="default" size="100%">Hamelryck, T.</style></author><author><style face="normal" font="default" size="100%">Kauff, F.</style></author><author><style face="normal" font="default" size="100%">Wilczynski, B.</style></author><author><style face="normal" font="default" size="100%">de Hoon, M. J.</style></author></authors></contributors><auth-address><style face="normal" font="default" size="100%">Plant Pathology, SCRI, Invergowrie, Dundee, UK. peter.cock@scri.ac.uk</style></auth-address><titles><title><style face="normal" font="default" size="100%">Biopython: freely available Python tools for computational molecular biology and bioinformatics</style></title><secondary-title><style face="normal" font="default" size="100%">Bioinformatics</style></secondary-title><alt-title><style face="normal" font="default" size="100%">Bioinformatics</style></alt-title></titles><periodical><full-title><style face="normal" font="default" size="100%">Bioinformatics</style></full-title><abbr-1><style face="normal" font="default" size="100%">Bioinformatics</style></abbr-1></periodical><alt-periodical><full-title><style face="normal" font="default" size="100%">Bioinformatics</style></full-title><abbr-1><style face="normal" font="default" size="100%">Bioinformatics</style></abbr-1></alt-periodical><pages><style face="normal" font="default" size="100%">1422-3</style></pages><volume><style face="normal" font="default" size="100%">25</style></volume><number><style face="normal" font="default" size="100%">11</style></number><keywords><keyword><style face="normal" font="default" size="100%">Computational Biology/*methods</style></keyword><keyword><style face="normal" font="default" size="100%">Databases, Factual</style></keyword><keyword><style face="normal" font="default" size="100%">Internet</style></keyword><keyword><style face="normal" font="default" size="100%">Programming Languages</style></keyword><keyword><style face="normal" font="default" size="100%">*Software</style></keyword></keywords><dates><year><style face="normal" font="default" size="100%">2009</style></year><pub-dates><date><style face="normal" font="default" size="100%">Jun 1</style></date></pub-dates></dates><isbn><style face="normal" font="default" size="100%">1367-4811 (Electronic)1367-4803 (Linking)</style></isbn><accession-num><style face="normal" font="default" size="100%">19304878</style></accession-num><abstract><style face="normal" font="default" size="100%">SUMMARY: The Biopython project is a mature open source international collaboration of volunteer developers, providing Python libraries for a wide range of bioinformatics problems. Biopython includes modules for reading and writing different sequence file formats and multiple sequence alignments, dealing with 3D macro molecular structures, interacting with common tools such as BLAST, ClustalW and EMBOSS, accessing key online databases, as well as providing numerical methods for statistical learning. AVAILABILITY: Biopython is freely available, with documentation and source code at (www.biopython.org) under the Biopython license.</style></abstract><notes><style face="normal" font="default" size="100%">Cock, Peter J AAntao, TiagoChang, Jeffrey TChapman, Brad ACox, Cymon JDalke, AndrewFriedberg, IddoHamelryck, ThomasKauff, FrankWilczynski, Bartekde Hoon, Michiel J LengR00 LM009837/LM/NLM NIH HHS/Research Support, Non-U.S. Gov'tEnglandOxford, England2009/03/24 09:00Bioinformatics. 2009 Jun 1;25(11):1422-3. doi: 10.1093/bioinformatics/btp163. Epub 2009 Mar 20.</style></notes><urls><related-urls><url><style face="normal" font="default" size="100%">http://www.ncbi.nlm.nih.gov/pubmed/19304878</style></url></related-urls></urls><custom2><style face="normal" font="default" size="100%">2682512</style></custom2><electronic-resource-num><style face="normal" font="default" size="100%">10.1093/bioinformatics/btp163</style></electronic-resource-num></record></Cite></EndNote>(2), helperlibs version 0.1.5, pysvg version 0.2.1, pyExcelerator version 0.6.4.1, ez-setup version 0.9, COBRA for Python version 0.2.1, GLPK version 0.3, BLAST+ version 2.2.30 ADDIN EN.CITE <EndNote><Cite><Author>Camacho, C.; Coulouris, G.; Avagyan, V.; Ma, N.; Papadopoulos, J.; Bealer, K.; Madden, T. L.</Author><Year>2009</Year><RecNum>189</RecNum><DisplayText>(3)</DisplayText><record><database name="My EndNote Library.enl" path="/Users/michaelskinnider/Documents/My EndNote Library.enl">My EndNote Library.enl</database><source-app name="EndNote" version="17.0">EndNote</source-app><rec-number>189</rec-number><foreign-keys><key app="EN" db-id="paazarvpaa559nes92r502wwvf52wxzszxxx">189</key></foreign-keys><ref-type name="Journal Article">17</ref-type><contributors><authors><author><style face="normal" font="default" size="100%">Camacho, C.</style></author><author><style face="normal" font="default" size="100%">Coulouris, G.</style></author><author><style face="normal" font="default" size="100%">Avagyan, V.</style></author><author><style face="normal" font="default" size="100%">Ma, N.</style></author><author><style face="normal" font="default" size="100%">Papadopoulos, J.</style></author><author><style face="normal" font="default" size="100%">Bealer, K.</style></author><author><style face="normal" font="default" size="100%">Madden, T. L.</style></author></authors></contributors><auth-address><style face="normal" font="default" size="100%">National Center for Biotechnology Information, National Library of Medicine, National Institutes of Health, Building 38A, 8600 Rockville Pike, Bethesda, MD 20894, USA. camacho@ncbi.nlm.nih.gov</style></auth-address><titles><title><style face="normal" font="default" size="100%">BLAST+: architecture and applications</style></title><secondary-title><style face="normal" font="default" size="100%">BMC Bioinformatics</style></secondary-title><alt-title><style face="normal" font="default" size="100%">BMC bioinformatics</style></alt-title></titles><periodical><full-title><style face="normal" font="default" size="100%">BMC Bioinformatics</style></full-title><abbr-1><style face="normal" font="default" size="100%">BMC bioinformatics</style></abbr-1></periodical><alt-periodical><full-title><style face="normal" font="default" size="100%">BMC Bioinformatics</style></full-title><abbr-1><style face="normal" font="default" size="100%">BMC bioinformatics</style></abbr-1></alt-periodical><pages><style face="normal" font="default" size="100%">421</style></pages><volume><style face="normal" font="default" size="100%">10</style></volume><keywords><keyword><style face="normal" font="default" size="100%">Computational Biology/*methods</style></keyword><keyword><style face="normal" font="default" size="100%">Databases, Genetic</style></keyword><keyword><style face="normal" font="default" size="100%">Sequence Alignment</style></keyword><keyword><style face="normal" font="default" size="100%">*Software</style></keyword></keywords><dates><year><style face="normal" font="default" size="100%">2009</style></year></dates><isbn><style face="normal" font="default" size="100%">1471-2105 (Electronic)1471-2105 (Linking)</style></isbn><accession-num><style face="normal" font="default" size="100%">20003500</style></accession-num><abstract><style face="normal" font="default" size="100%">BACKGROUND: Sequence similarity searching is a very important bioinformatics task. While Basic Local Alignment Search Tool (BLAST) outperforms exact methods through its use of heuristics, the speed of the current BLAST software is suboptimal for very long queries or database sequences. There are also some shortcomings in the user-interface of the current command-line applications. RESULTS: We describe features and improvements of rewritten BLAST software and introduce new command-line applications. Long query sequences are broken into chunks for processing, in some cases leading to dramatically shorter run times. For long database sequences, it is possible to retrieve only the relevant parts of the sequence, reducing CPU time and memory usage for searches of short queries against databases of contigs or chromosomes. The program can now retrieve masking information for database sequences from the BLAST databases. A new modular software library can now access subject sequence data from arbitrary data sources. We introduce several new features, including strategy files that allow a user to save and reuse their favorite set of options. The strategy files can be uploaded to and downloaded from the NCBI BLAST web site. CONCLUSION: The new BLAST command-line applications, compared to the current BLAST tools, demonstrate substantial speed improvements for long queries as well as chromosome length database sequences. We have also improved the user interface of the command-line applications.</style></abstract><notes><style face="normal" font="default" size="100%">Camacho, ChristiamCoulouris, GeorgeAvagyan, VahramMa, NingPapadopoulos, JasonBealer, KevinMadden, Thomas LengResearch Support, N.I.H., ExtramuralEngland2009/12/17 06:00BMC Bioinformatics. 2009 Dec 15;10:421. doi: 10.1186/1471-2105-10-421.</style></notes><urls><related-urls><url><style face="normal" font="default" size="100%">http://www.ncbi.nlm.nih.gov/pubmed/20003500</style></url></related-urls></urls><custom2><style face="normal" font="default" size="100%">2803857</style></custom2><electronic-resource-num><style face="normal" font="default" size="100%">10.1186/1471-2105-10-421</style></electronic-resource-num></record></Cite></EndNote>(3), and DIAMOND version 0.7.9.58 ADDIN EN.CITE <EndNote><Cite><Author>Buchfink, B.; Xie, C.; Huson, D. H.</Author><Year>2015</Year><RecNum>190</RecNum><DisplayText>(4)</DisplayText><record><database name="My EndNote Library.enl" path="/Users/michaelskinnider/Documents/My EndNote Library.enl">My EndNote Library.enl</database><source-app name="EndNote" version="17.0">EndNote</source-app><rec-number>190</rec-number><foreign-keys><key app="EN" db-id="paazarvpaa559nes92r502wwvf52wxzszxxx">190</key></foreign-keys><ref-type name="Journal Article">17</ref-type><contributors><authors><author><style face="normal" font="default" size="100%">Buchfink, B.</style></author><author><style face="normal" font="default" size="100%">Xie, C.</style></author><author><style face="normal" font="default" size="100%">Huson, D. H.</style></author></authors></contributors><auth-address><style face="normal" font="default" size="100%">Department of Computer Science and Center for Bioinformatics, University of Tubingen, Tubingen, Germany.1] Singapore Centre on Environmental Life Sciences Engineering, School of Biological Sciences, Nanyang Technological University, Singapore. [2] Life Sciences Institute, National University of Singapore, Singapore.1] Department of Computer Science and Center for Bioinformatics, University of Tubingen, Tubingen, Germany. [2] Singapore Centre on Environmental Life Sciences Engineering, School of Biological Sciences, Nanyang Technological University, Singapore.</style></auth-address><titles><title><style face="normal" font="default" size="100%">Fast and sensitive protein alignment using DIAMOND</style></title><secondary-title><style face="normal" font="default" size="100%">Nat Methods</style></secondary-title><alt-title><style face="normal" font="default" size="100%">Nature methods</style></alt-title></titles><periodical><full-title><style face="normal" font="default" size="100%">Nat Methods</style></full-title><abbr-1><style face="normal" font="default" size="100%">Nature methods</style></abbr-1></periodical><alt-periodical><full-title><style face="normal" font="default" size="100%">Nat Methods</style></full-title><abbr-1><style face="normal" font="default" size="100%">Nature methods</style></abbr-1></alt-periodical><pages><style face="normal" font="default" size="100%">59-60</style></pages><volume><style face="normal" font="default" size="100%">12</style></volume><number><style face="normal" font="default" size="100%">1</style></number><keywords><keyword><style face="normal" font="default" size="100%">Algorithms</style></keyword><keyword><style face="normal" font="default" size="100%">Base Sequence</style></keyword><keyword><style face="normal" font="default" size="100%">Humans</style></keyword><keyword><style face="normal" font="default" size="100%">Metagenomics/*methods</style></keyword><keyword><style face="normal" font="default" size="100%">Microbiota/genetics</style></keyword><keyword><style face="normal" font="default" size="100%">Sensitivity and Specificity</style></keyword><keyword><style face="normal" font="default" size="100%">Sequence Alignment/*methods</style></keyword><keyword><style face="normal" font="default" size="100%">Sequence Analysis, DNA</style></keyword><keyword><style face="normal" font="default" size="100%">*Software</style></keyword></keywords><dates><year><style face="normal" font="default" size="100%">2015</style></year><pub-dates><date><style face="normal" font="default" size="100%">Jan</style></date></pub-dates></dates><isbn><style face="normal" font="default" size="100%">1548-7105 (Electronic)1548-7091 (Linking)</style></isbn><accession-num><style face="normal" font="default" size="100%">25402007</style></accession-num><abstract><style face="normal" font="default" size="100%">The alignment of sequencing reads against a protein reference database is a major computational bottleneck in metagenomics and data-intensive evolutionary projects. Although recent tools offer improved performance over the gold standard BLASTX, they exhibit only a modest speedup or low sensitivity. We introduce DIAMOND, an open-source algorithm based on double indexing that is 20,000 times faster than BLASTX on short reads and has a similar degree of sensitivity.</style></abstract><notes><style face="normal" font="default" size="100%">Buchfink, BenjaminXie, ChaoHuson, Daniel HengComparative StudyResearch Support, Non-U.S. Gov't2014/11/18 06:00Nat Methods. 2015 Jan;12(1):59-60. doi: 10.1038/nmeth.3176. Epub 2014 Nov 17.</style></notes><urls><related-urls><url><style face="normal" font="default" size="100%">http://www.ncbi.nlm.nih.gov/pubmed/25402007</style></url></related-urls></urls><electronic-resource-num><style face="normal" font="default" size="100%">10.1038/nmeth.3176</style></electronic-resource-num></record></Cite></EndNote>(4) were installed to satisfy dependencies. NP.searcher (version 1.01) ADDIN EN.CITE <EndNote><Cite><Author>Li, M. H.; Ung, P. M.; Zajkowski, J.; Garneau-Tsodikova, S.; Sherman, D. H.</Author><Year>2009</Year><RecNum>29</RecNum><DisplayText>(5)</DisplayText><record><database name="My EndNote Library.enl" path="/Users/michaelskinnider/Documents/My EndNote Library.enl">My EndNote Library.enl</database><source-app name="EndNote" version="17.0">EndNote</source-app><rec-number>29</rec-number><foreign-keys><key app="EN" db-id="paazarvpaa559nes92r502wwvf52wxzszxxx">29</key></foreign-keys><ref-type name="Journal Article">17</ref-type><contributors><authors><author><style face="normal" font="default" size="100%">Li, M. H.</style></author><author><style face="normal" font="default" size="100%">Ung, P. M.</style></author><author><style face="normal" font="default" size="100%">Zajkowski, J.</style></author><author><style face="normal" font="default" size="100%">Garneau-Tsodikova, S.</style></author><author><style face="normal" font="default" size="100%">Sherman, D. H.</style></author></authors></contributors><auth-address><style face="normal" font="default" size="100%">Life Sciences Institute, University of Michigan, Ann Arbor, MI, USA. mikeleez@umich.edu</style></auth-address><titles><title><style face="normal" font="default" size="100%">Automated genome mining for natural products</style></title><secondary-title><style face="normal" font="default" size="100%">BMC Bioinformatics</style></secondary-title><alt-title><style face="normal" font="default" size="100%">BMC bioinformatics</style></alt-title></titles><periodical><full-title><style face="normal" font="default" size="100%">BMC Bioinformatics</style></full-title><abbr-1><style face="normal" font="default" size="100%">BMC bioinformatics</style></abbr-1></periodical><alt-periodical><full-title><style face="normal" font="default" size="100%">BMC Bioinformatics</style></full-title><abbr-1><style face="normal" font="default" size="100%">BMC bioinformatics</style></abbr-1></alt-periodical><pages><style face="normal" font="default" size="100%">185</style></pages><volume><style face="normal" font="default" size="100%">10</style></volume><keywords><keyword><style face="normal" font="default" size="100%">Biological Products/*chemistry</style></keyword><keyword><style face="normal" font="default" size="100%">Computational Biology/*methods</style></keyword><keyword><style face="normal" font="default" size="100%">*Genome</style></keyword><keyword><style face="normal" font="default" size="100%">Internet</style></keyword><keyword><style face="normal" font="default" size="100%">Macrolides/chemistry</style></keyword></keywords><dates><year><style face="normal" font="default" size="100%">2009</style></year></dates><isbn><style face="normal" font="default" size="100%">1471-2105 (Electronic)1471-2105 (Linking)</style></isbn><accession-num><style face="normal" font="default" size="100%">19531248</style></accession-num><abstract><style face="normal" font="default" size="100%">BACKGROUND: Discovery of new medicinal agents from natural sources has largely been an adventitious process based on screening of plant and microbial extracts combined with bioassay-guided identification and natural product structure elucidation. Increasingly rapid and more cost-effective genome sequencing technologies coupled with advanced computational power have converged to transform this trend toward a more rational and predictive pursuit. RESULTS: We have developed a rapid method of scanning genome sequences for multiple polyketide, nonribosomal peptide, and mixed combination natural products with output in a text format that can be readily converted to two and three dimensional structures using conventional software. Our open-source and web-based program can assemble various small molecules composed of twenty standard amino acids and twenty two other chain-elongation intermediates used in nonribosomal peptide systems, and four acyl-CoA extender units incorporated into polyketides by reading a hidden Markov model of DNA. This process evaluates and selects the substrate specificities along the assembly line of nonribosomal synthetases and modular polyketide synthases. CONCLUSION: Using this approach we have predicted the structures of natural products from a diverse range of bacteria based on a limited number of signature sequences. In accelerating direct DNA to metabolomic analysis, this method bridges the interface between chemists and biologists and enables rapid scanning for compounds with potential therapeutic value.</style></abstract><notes><style face="normal" font="default" size="100%">Li, Michael H TUng, Peter M UZajkowski, JamesGarneau-Tsodikova, SylvieSherman, David HengGM076477/GM/NIGMS NIH HHS/Research Support, N.I.H., ExtramuralResearch Support, Non-U.S. Gov'tEngland2009/06/18 09:00BMC Bioinformatics. 2009 Jun 16;10:185. doi: 10.1186/1471-2105-10-185.</style></notes><urls><related-urls><url><style face="normal" font="default" size="100%">http://www.ncbi.nlm.nih.gov/pubmed/19531248</style></url><url><style face="normal" font="default" size="100%">http://www.ncbi.nlm.nih.gov/pmc/articles/PMC2712472/pdf/1471-2105-10-185.pdf</style></url></related-urls><pdf-urls><url>internal-pdf://1101513929/Li-2009-Automated genome min.pdf</url></pdf-urls></urls><custom2><style face="normal" font="default" size="100%">2712472</style></custom2><electronic-resource-num><style face="normal" font="default" size="100%">10.1186/1471-2105-10-185</style></electronic-resource-num></record></Cite></EndNote>(5) source code was obtained from http://dna.sherman.lsi.umich.edu/, and run with blastall version 2.2.22 on Mac OS X 10.10.1. NP.searcher was run with default mass window parameters (1–5,000 Da), with the cyclization, glycosylation, and dimerization options enabled. antiSMASH was run with the all-orfs option in order to detect all possible open reading frames and Pfam version 28.0 ADDIN EN.CITE <EndNote><Cite><Author>Finn, R. D.; Bateman, A.; Clements, J.; Coggill, P.; Eberhardt, R. Y.; Eddy, S. R.; Heger, A.; Hetherington, K.; Holm, L.; Mistry, J.; Sonnhammer, E. L.; Tate, J.; Punta, M.</Author><Year>2014</Year><RecNum>7</RecNum><DisplayText>(6)</DisplayText><record><database name="My EndNote Library.enl" path="/Users/michaelskinnider/Documents/My EndNote Library.enl">My EndNote Library.enl</database><source-app name="EndNote" version="17.0">EndNote</source-app><rec-number>7</rec-number><foreign-keys><key app="EN" db-id="paazarvpaa559nes92r502wwvf52wxzszxxx">7</key></foreign-keys><ref-type name="Journal Article">17</ref-type><contributors><authors><author><style face="normal" font="default" size="100%">Finn, R. D.</style></author><author><style face="normal" font="default" size="100%">Bateman, A.</style></author><author><style face="normal" font="default" size="100%">Clements, J.</style></author><author><style face="normal" font="default" size="100%">Coggill, P.</style></author><author><style face="normal" font="default" size="100%">Eberhardt, R. Y.</style></author><author><style face="normal" font="default" size="100%">Eddy, S. R.</style></author><author><style face="normal" font="default" size="100%">Heger, A.</style></author><author><style face="normal" font="default" size="100%">Hetherington, K.</style></author><author><style face="normal" font="default" size="100%">Holm, L.</style></author><author><style face="normal" font="default" size="100%">Mistry, J.</style></author><author><style face="normal" font="default" size="100%">Sonnhammer, E. L.</style></author><author><style face="normal" font="default" size="100%">Tate, J.</style></author><author><style face="normal" font="default" size="100%">Punta, M.</style></author></authors></contributors><auth-address><style face="normal" font="default" size="100%">HHMI Janelia Farm Research Campus, 19700 Helix Drive, Ashburn, VA 20147 USA, European Molecular Biology Laboratory, European Bioinformatics Institute (EMBL-EBI), Wellcome Trust Genome Campus, Hinxton, Cambridge CB10 1SD, UK, Wellcome Trust Sanger Institute, Wellcome Trust Genome Campus, Hinxton, Cambridge CB10 1SA, UK, MRC Functional Genomics Unit, Department of Physiology, Anatomy and Genetics, University of Oxford, Oxford, OX1 3QX, UK, Institute of Biotechnology and Department of Biological and Environmental Sciences, University of Helsinki, PO Box 56 (Viikinkaari 5), 00014 Helsinki, Finland and Stockholm Bioinformatics Center, Swedish eScience Research Center, Department of Biochemistry and Biophysics, Science for Life Laboratory, Stockholm University, PO Box 1031, SE-17121 Solna, Sweden.</style></auth-address><titles><title><style face="normal" font="default" size="100%">Pfam: the protein families database</style></title><secondary-title><style face="normal" font="default" size="100%">Nucleic Acids Res</style></secondary-title><alt-title><style face="normal" font="default" size="100%">Nucleic acids research</style></alt-title></titles><periodical><full-title><style face="normal" font="default" size="100%">Nucleic Acids Res</style></full-title><abbr-1><style face="normal" font="default" size="100%">Nucleic acids research</style></abbr-1></periodical><alt-periodical><full-title><style face="normal" font="default" size="100%">Nucleic Acids Res</style></full-title><abbr-1><style face="normal" font="default" size="100%">Nucleic acids research</style></abbr-1></alt-periodical><pages><style face="normal" font="default" size="100%">D222-30</style></pages><volume><style face="normal" font="default" size="100%">42</style></volume><number><style face="normal" font="default" size="100%">Database issue</style></number><keywords><keyword><style face="normal" font="default" size="100%">*Databases, Protein</style></keyword><keyword><style face="normal" font="default" size="100%">Internet</style></keyword><keyword><style face="normal" font="default" size="100%">Intrinsically Disordered Proteins/chemistry</style></keyword><keyword><style face="normal" font="default" size="100%">Protein Conformation</style></keyword><keyword><style face="normal" font="default" size="100%">Proteins/chemistry/classification/genetics</style></keyword><keyword><style face="normal" font="default" size="100%">Proteome/chemistry</style></keyword><keyword><style face="normal" font="default" size="100%">*Sequence Alignment</style></keyword><keyword><style face="normal" font="default" size="100%">Sequence Analysis, DNA</style></keyword><keyword><style face="normal" font="default" size="100%">*Sequence Analysis, Protein</style></keyword></keywords><dates><year><style face="normal" font="default" size="100%">2014</style></year><pub-dates><date><style face="normal" font="default" size="100%">Jan</style></date></pub-dates></dates><isbn><style face="normal" font="default" size="100%">1362-4962 (Electronic)0305-1048 (Linking)</style></isbn><accession-num><style face="normal" font="default" size="100%">24288371</style></accession-num><abstract><style face="normal" font="default" size="100%">Pfam, available via servers in the UK (http://pfam.sanger.ac.uk/) and the USA (http://pfam.janelia.org/), is a widely used database of protein families, containing 14 831 manually curated entries in the current release, version 27.0. Since the last update article 2 years ago, we have generated 1182 new families and maintained sequence coverage of the UniProt Knowledgebase (UniProtKB) at nearly 80%, despite a 50% increase in the size of the underlying sequence database. Since our 2012 article describing Pfam, we have also undertaken a comprehensive review of the features that are provided by Pfam over and above the basic family data. For each feature, we determined the relevance, computational burden, usage statistics and the functionality of the feature in a website context. As a consequence of this review, we have removed some features, enhanced others and developed new ones to meet the changing demands of computational biology. Here, we describe the changes to Pfam content. Notably, we now provide family alignments based on four different representative proteome sequence data sets and a new interactive DNA search interface. We also discuss the mapping between Pfam and known 3D structures.</style></abstract><notes><style face="normal" font="default" size="100%">Finn, Robert DBateman, AlexClements, JodyCoggill, PenelopeEberhardt, Ruth YEddy, Sean RHeger, AndreasHetherington, KirstieHolm, LiisaMistry, JainaSonnhammer, Erik L LTate, JohnPunta, MarcoengHoward Hughes Medical Institute/Research Support, Non-U.S. Gov'tEngland2013/11/30 06:00Nucleic Acids Res. 2014 Jan;42(Database issue):D222-30. doi: 10.1093/nar/gkt1223. Epub 2013 Nov 27.</style></notes><urls><related-urls><url><style face="normal" font="default" size="100%">http://www.ncbi.nlm.nih.gov/pubmed/24288371</style></url><url><style face="normal" font="default" size="100%">http://www.ncbi.nlm.nih.gov/pmc/articles/PMC3965110/pdf/gkt1223.pdf</style></url></related-urls><pdf-urls><url>internal-pdf://2962586513/Finn-2014-Pfam_ the protein fa.pdf</url></pdf-urls></urls><custom2><style face="normal" font="default" size="100%">3965110</style></custom2><electronic-resource-num><style face="normal" font="default" size="100%">10.1093/nar/gkt1223</style></electronic-resource-num></record></Cite></EndNote>(6). PRISM was run with a cluster window of 20,000 base pairs. Because both PRISM and NP.searcher generate combinatorial libraries of predicted structures of variable sizes, a maximum size of 50 was imposed on combinatorial library size. The maximum scaffold library size was set to 50 within PRISM while only the first 50 hypothetical structures output by NP.searcher were considered. For each biosynthetic gene cluster, both the average Tanimoto coefficient and the single highest Tanimoto coefficient were calculated for PRISM and NP.searcher; for antiSMASH, which generates a single predicted structure, these coefficients were equivalent. When a software package failed to detect a biosynthetic gene cluster or generate a predicted structure, a score of 0 was assigned. Structures with invalid SMILES were discarded.

*Comparison to real structures.* Chemical fingerprints were generated for real natural product structures and all predicted structures with the Chemistry Development Kit ADDIN EN.CITE <EndNote><Cite><Author>Steinbeck, C.; Han, Y.; Kuhn, S.; Horlacher, O.; Luttmann, E.; Willighagen, E.</Author><Year>2003</Year><RecNum>1</RecNum><DisplayText>(7)</DisplayText><record><database name="My EndNote Library.enl" path="/Users/michaelskinnider/Documents/My EndNote Library.enl">My EndNote Library.enl</database><source-app name="EndNote" version="17.0">EndNote</source-app><rec-number>1</rec-number><foreign-keys><key app="EN" db-id="paazarvpaa559nes92r502wwvf52wxzszxxx">1</key></foreign-keys><ref-type name="Journal Article">17</ref-type><contributors><authors><author><style face="normal" font="default" size="100%">Steinbeck, C.</style></author><author><style face="normal" font="default" size="100%">Han, Y.</style></author><author><style face="normal" font="default" size="100%">Kuhn, S.</style></author><author><style face="normal" font="default" size="100%">Horlacher, O.</style></author><author><style face="normal" font="default" size="100%">Luttmann, E.</style></author><author><style face="normal" font="default" size="100%">Willighagen, E.</style></author></authors></contributors><auth-address><style face="normal" font="default" size="100%">Max-Planck-Institute of Chemical Ecology, Jena, Germany. c.steinbeck@uni-koeln.de</style></auth-address><titles><title><style face="normal" font="default" size="100%">The Chemistry Development Kit (CDK): an open-source Java library for Chemo- and Bioinformatics</style></title><secondary-title><style face="normal" font="default" size="100%">J Chem Inf Comput Sci</style></secondary-title><alt-title><style face="normal" font="default" size="100%">Journal of chemical information and computer sciences</style></alt-title></titles><periodical><full-title><style face="normal" font="default" size="100%">J Chem Inf Comput Sci</style></full-title><abbr-1><style face="normal" font="default" size="100%">Journal of chemical information and computer sciences</style></abbr-1></periodical><alt-periodical><full-title><style face="normal" font="default" size="100%">J Chem Inf Comput Sci</style></full-title><abbr-1><style face="normal" font="default" size="100%">Journal of chemical information and computer sciences</style></abbr-1></alt-periodical><pages><style face="normal" font="default" size="100%">493-500</style></pages><volume><style face="normal" font="default" size="100%">43</style></volume><number><style face="normal" font="default" size="100%">2</style></number><dates><year><style face="normal" font="default" size="100%">2003</style></year><pub-dates><date><style face="normal" font="default" size="100%">Mar-Apr</style></date></pub-dates></dates><isbn><style face="normal" font="default" size="100%">0095-2338 (Print)0095-2338 (Linking)</style></isbn><accession-num><style face="normal" font="default" size="100%">12653513</style></accession-num><abstract><style face="normal" font="default" size="100%">The Chemistry Development Kit (CDK) is a freely available open-source Java library for Structural Chemo- and Bioinformatics. Its architecture and capabilities as well as the development as an open-source project by a team of international collaborators from academic and industrial institutions is described. The CDK provides methods for many common tasks in molecular informatics, including 2D and 3D rendering of chemical structures, I/O routines, SMILES parsing and generation, ring searches, isomorphism checking, structure diagram generation, etc. Application scenarios as well as access information for interested users and potential contributors are given.</style></abstract><notes><style face="normal" font="default" size="100%">Steinbeck, ChristophHan, YongquanKuhn, StefanHorlacher, OliverLuttmann, EdgarWillighagen, Egoneng2003/03/26 05:00J Chem Inf Comput Sci. 2003 Mar-Apr;43(2):493-500.</style></notes><urls><related-urls><url><style face="normal" font="default" size="100%">http://www.ncbi.nlm.nih.gov/pubmed/12653513</style></url><url><style face="normal" font="default" size="100%">http://pubs.acs.org/doi/pdfplus/10.1021/ci025584y</style></url></related-urls><pdf-urls><url>internal-pdf://2002445656/Steinbeck-2003-The Chemistry Develo.pdf</url><url>internal-pdf://2639285226/Steinbeck-2003-The Chemistry Develo1.pdf</url></pdf-urls></urls><electronic-resource-num><style face="normal" font="default" size="100%">10.1021/ci025584y</style></electronic-resource-num></record></Cite></EndNote>(7) (version 1.5.6) implementation of the ECFP6 fingerprinting algorithm ADDIN EN.CITE <EndNote><Cite><Author>Rogers, D.; Hahn, M.</Author><Year>2010</Year><RecNum>47</RecNum><DisplayText>(8)</DisplayText><record><source-app name="EndNote" version="17.0">EndNote</source-app><rec-number>47</rec-number><foreign-keys><key app="EN" db-id="0adf902aasawfves2d8pdr5yd5epdf9rxxzt">47</key><key app="ENWeb" db-id="">0</key></foreign-keys><ref-type name="Journal Article">17</ref-type><contributors><authors><author><style face="normal" font="default" size="100%">Rogers, D.</style></author><author><style face="normal" font="default" size="100%">Hahn, M.</style></author></authors></contributors><auth-address><style face="normal" font="default" size="100%">Rogers, D3429 N Mt View Dr, San Diego, Ca 92116 USA3429 N Mt View Dr, San Diego, Ca 92116 USAAccelrys Inc, San Diego, CA 92121 USA</style></auth-address><titles><title><style face="normal" font="default" size="100%">Extended-Connectivity Fingerprints</style></title><secondary-title><style face="normal" font="default" size="100%">Journal of Chemical Information and Modeling</style></secondary-title><alt-title><style face="normal" font="default" size="100%">J Chem Inf Model</style></alt-title></titles><pages><style face="normal" font="default" size="100%">742-754</style></pages><volume><style face="normal" font="default" size="100%">50</style></volume><number><style face="normal" font="default" size="100%">5</style></number><dates><year><style face="normal" font="default" size="100%">2010</style></year><pub-dates><date><style face="normal" font="default" size="100%">May</style></date></pub-dates></dates><isbn><style face="normal" font="default" size="100%">1549-9596</style></isbn><accession-num><style face="normal" font="default" size="100%">WOS:000277911600004</style></accession-num><abstract><style face="normal" font="default" size="100%">Extended-connectivity fingerprints (ECEPs) are a novel class of topological fingerprints for molecular characterization. Historically, topological fingerprints were developed for substructure and similarity searching. ECEPs were developed specifically for structure activity modeling. ECEPs are circular fingerprints with a number of useful qualities: they can be very rapidly calculated; they are not predefined and can represent an essentially infinite number of different molecular features (including stereochemical information); their features represent the presence of particular substructures, allowing easier interpretation of analysis results; and the ECFP algorithm can he tailored to generate different types of circular fingerprints, optimized for different uses. While the use of ECEPs has been widely adopted and validated, a description of their implementation has not previously been presented in the literature.</style></abstract><notes><style face="normal" font="default" size="100%">599KSTimes Cited:252Cited References Count:107</style></notes><urls><related-urls><url><style face="normal" font="default" size="100%">Go to ISI://WOS:000277911600004</style></url></related-urls></urls><electronic-resource-num><style face="normal" font="default" size="100%">Doi 10.1021/Ci100050t</style></electronic-resource-num><language><style face="normal" font="default" size="100%">English</style></language></record></Cite></EndNote>(8). Sites of variability or uncertainty, denoted in SMILES output by antiSMASH as [Rn], where n is an integer, and in SMILES output by NP.searcher as [X], were replaced with asterisks in order to parse predicted structures with the Chemistry Development Kit. Fingerprints were converted to bit sets with a length of 1,024 bits and Tanimoto coefficients were computed with the Chemistry Development Kit.

# REFERENCES

1. Weber,T., Blin,K., Duddela,S., Krug,D., Kim,H.U., Bruccoleri,R., Lee,S.Y., Fischbach,M.A., Muller,R., Wohlleben,W. et al. (2015) antiSMASH 3.0—a comprehensive resource for the genome mining of biosynthetic gene clusters. *Nucleic Acids Res.*, **43**, W237-W243.

2. Cock,P.J., Antao,T., Chang,J.T., Chapman,B.A., Cox,C.J., Dalke,A., Friedberg,I., Hamelryck,T., Kauff,F., Wilczynski,B. et al. (2009) Biopython: freely available Python tools for computational molecular biology and bioinformatics. *Bioinformatics*, **25**, 1422-1423.

3. Camacho,C., Coulouris,G., Avagyan,V., Ma,N., Papadopoulos,J., Bealer,K. and Madden,T.L. (2009) BLAST+: architecture and applications. *BMC Bioinformatics*, **10**, 421.

4. Buchfink,B., Xie,C. and Huson,D.H. (2015) Fast and sensitive protein alignment using DIAMOND. *Nat. Methods*, **12**, 59-60.

5. Li,M.H., Ung,P.M., Zajkowski,J., Garneau-Tsodikova,S. and Sherman,D.H. (2009) Automated genome mining for natural products. *BMC Bioinformatics*, **10**, 185.

6. Finn,R.D., Bateman,A., Clements,J., Coggill,P., Eberhardt,R.Y., Eddy,S.R., Heger,A., Hetherington,K., Holm,L., Mistry,J. et al. (2014) Pfam: the protein families database. *Nucleic Acids Res.*, **42**, D222-230.

7. Steinbeck,C., Han,Y., Kuhn,S., Horlacher,O., Luttmann,E. and Willighagen,E. (2003) The Chemistry Development Kit (CDK): an open-source Java library for Chemo- and Bioinformatics. *J. Chem. Inf. Comput. Sci.*, **43**, 493-500.

8. Rogers,D. and Hahn,M. (2010) Extended-Connectivity Fingerprints. *J. Chem. Inf. Model.*, **50**, 742-754.
