# Supplementary material for: Genomes to natural products PRediction Informatics for Secondary Metabolomes (PRISM)
Source: Nucleic Acids Res. 2015 Oct 5;43(20):9645–62. doi: 10.1093/nar/gkv1012 (PMC4787774; doi:10.1093/nar/gkv1012)
Supplement: SUPPLEMENTARY DATA [file supp_gkv1012_nar-01872-z-2015-File015.pdf]

Cluster	Compound order	Open reading frame permutations	Sugars	Cyclizations	Tailoring reaction plans	Combinatorial plans	Total number of scaffolds generated	True structure	Median Tanimoto coefficient	Median structures	Top Tanimoto coefficient	Top structures
cephalosporin	beta Lactam	1	0	1	1	1	1	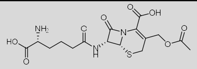	0.734	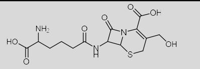	0.734	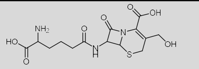
cephamycin C	beta Lactam	1	0	1	1	1	1	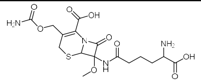	0.52	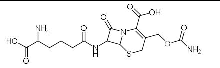	0.52	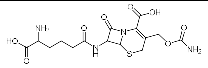
penicillin	beta Lactam	1	0	1	1	1	1	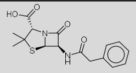	1	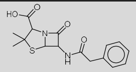	1	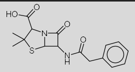
anabaenopeptin	Cyclic branched	1	0	2	1	2	2	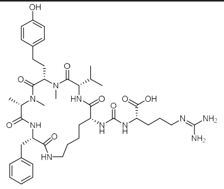	0.261	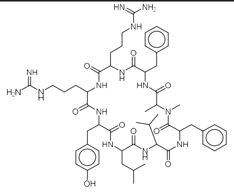	0.261	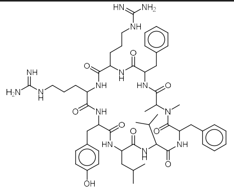
bacitracin	Cyclic branched	1	0	2	1	2	2	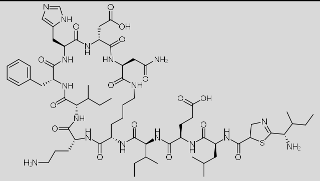	0.332	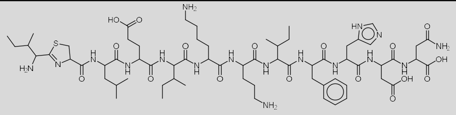	0.332	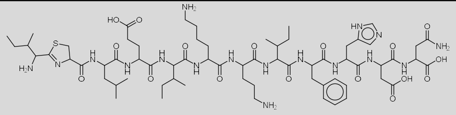
bassianolide	Cyclic branched	1	0	1	1	1	1	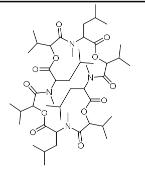	0.143	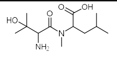	0.143	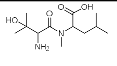
beauvericin	Cyclic branched	1	0	1	1	1	1	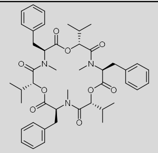	0.079	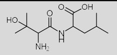	0.079	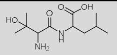
capreomycin	Cyclic branched	1	0	4	1	4	4	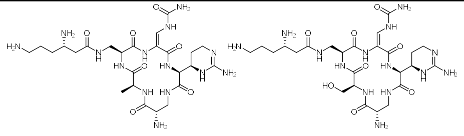	0.329	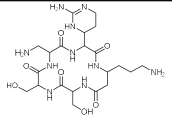	0.331	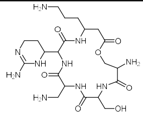
cereulide	Cyclic branched	1	0	2	1	2	2	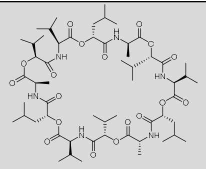	0.462	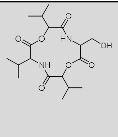	0.462	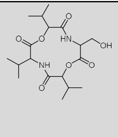
cryptophycin	Cyclic branched	1	0	4	1	4	2	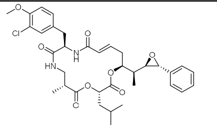	0.34	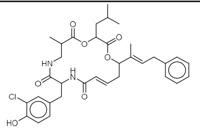	0.34	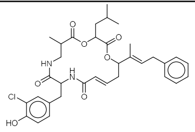
cyclosporine	Cyclic branched	1	0	2	1	2	2	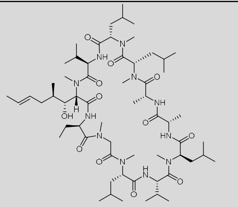	0.814	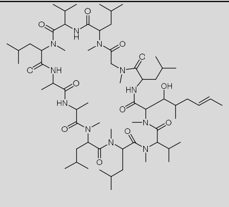	0.814	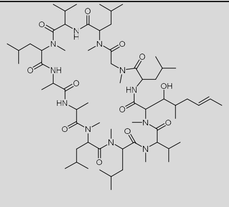

echinomycin	Cyclic branched	1	0	3	1	3	2	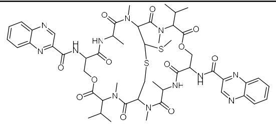	0.258	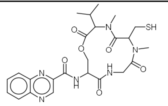	0.258	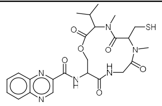
enniatin	Cyclic branched	1	0	1	1	1	1	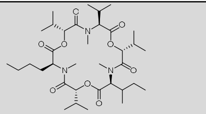	0.071	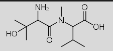	0.071	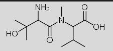
gramicidin	Cyclic branched	1	0	2	1	2	2	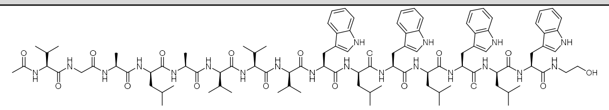	0.396	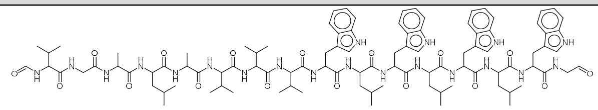	0.396	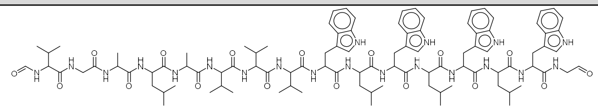
griselimycin	Cyclic branched	1	0	4	1	4	4	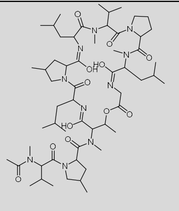	0.257	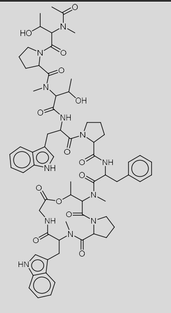	0.284	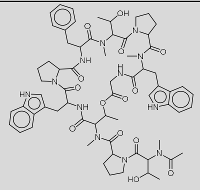
hormaomycin	Cyclic branched	1	0	2	1	2	2	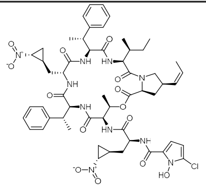	0.242	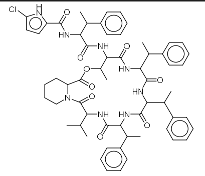	0.242	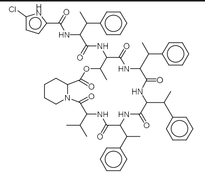
koranimine	Cyclic branched	1	0	2	1	2	2	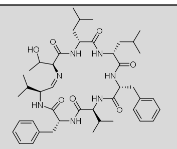	0.438	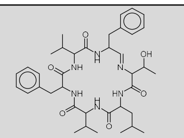	0.438	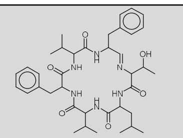
microcystin	Cyclic branched	24	0	4	24	114	66	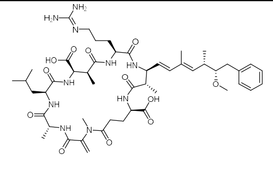	0.207	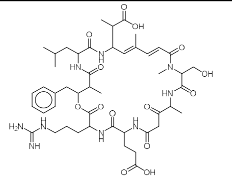	0.257	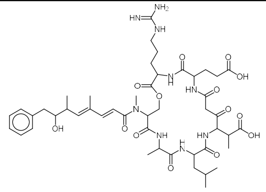
nostocyclopeptide	Cyclic branched	1	0	2	1	2	2	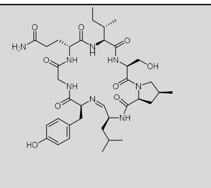	0.644	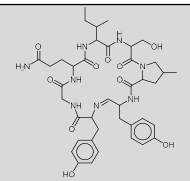	0.644	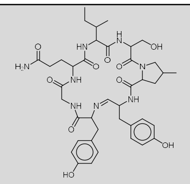
nostophycin	Cyclic branched	1	0	5	1	5	3	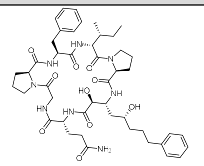	0.256	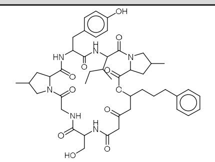	0.271	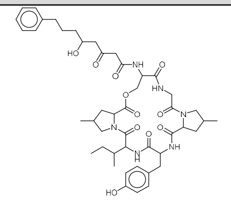
SW-163	Cyclic branched	1	0	3	1	3	2	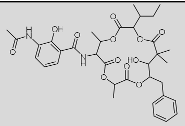	0.11	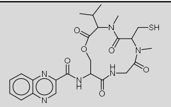	0.11	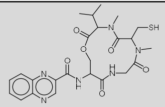

teixobactin	Cyclic branched	1	0	5	1	5	5	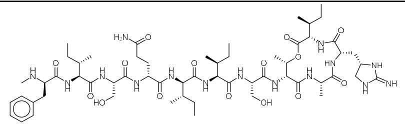	0.34	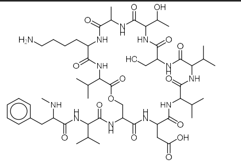	0.542	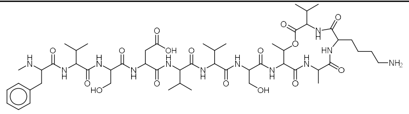
telomycin	Cyclic branched	1	0	6	1	6	5	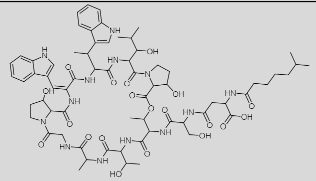	0.255	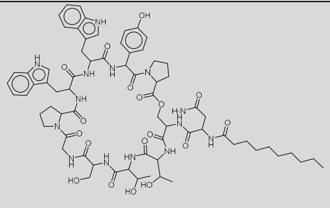	0.317	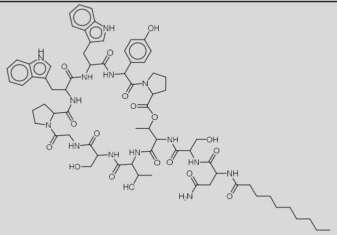
thiocoraline	Cyclic branched	1	0	4	1	4	3	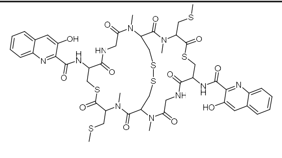	0.169	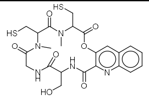	0.219	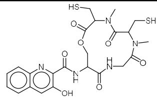
triostin A	Cyclic branched	1	0	3	1	3	2	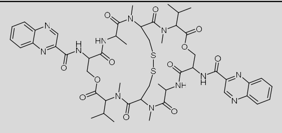	0.285	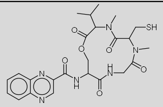	0.285	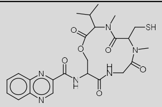
tyrocydine	Cyclic branched	1	0	2	1	2	2	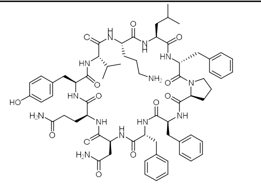	0.586	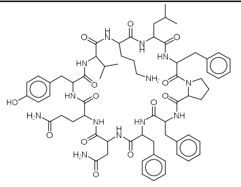	0.586	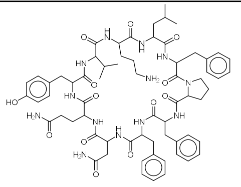
valinomycin	Cyclic branched	1	0	2	1	2	2	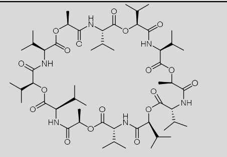	1	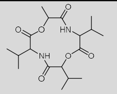	1	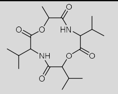
viomycin	Cyclic branched	1	0	4	2	8	6	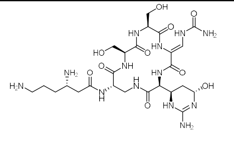	0.221	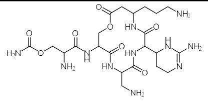	0.264	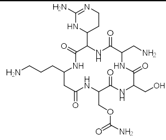
A-74528	Glycopeptides	1	0	1	1	1	1	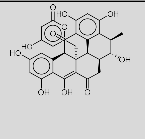	0.112	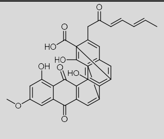	0.112	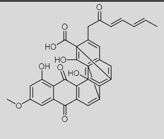
A40926	Glycopeptides	6	1	1	2058	1,000+	246	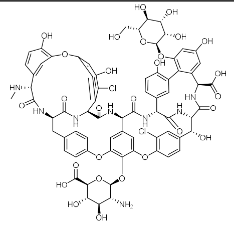	0.188	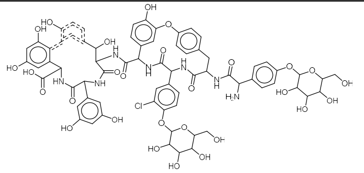	0.205	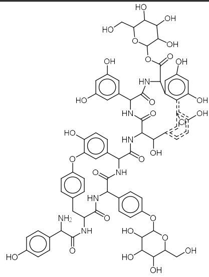
balhimycin	Glycopeptides	2	9	1	4116	1,000+	74	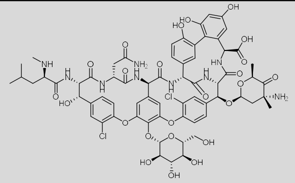	0.238	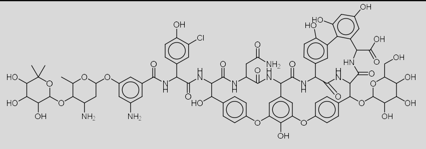	0.259	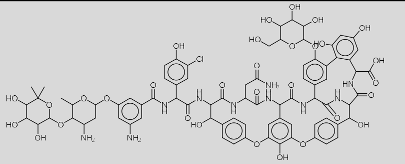

bleomycin	Glycopeptides	500+	0	5	1706	1,000+	379	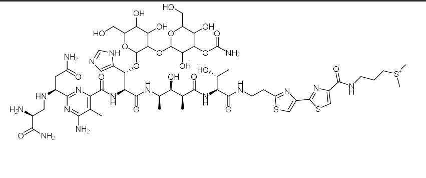	0.182	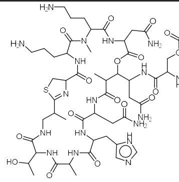	0.24	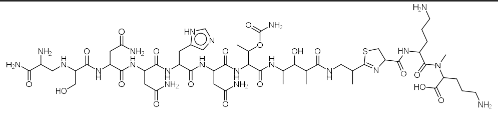
complestatin	Glycopeptides	1	0	2	6	12	12	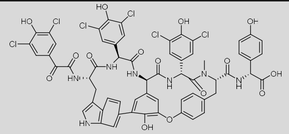	0.15	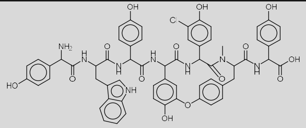	0.167	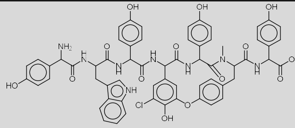
mannopeptimycin	Glycopeptides	1	1	3	1	3	3	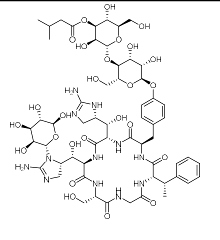	0.181	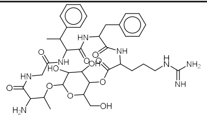	0.238	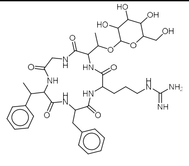
ramoplanin	Glycopeptides	6	0	4	54	252	132	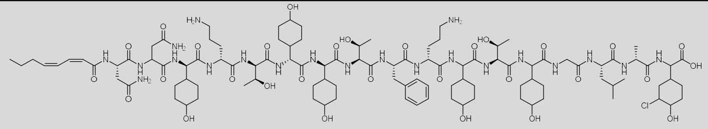	0.348	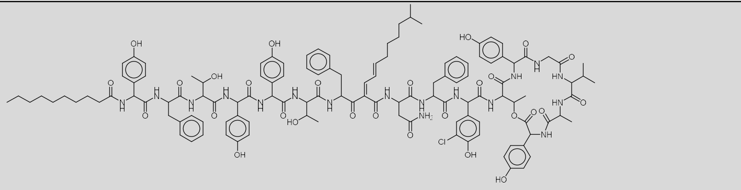	0.516	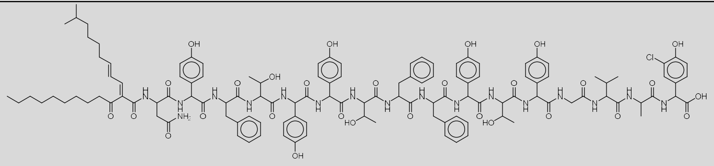
ristocetin	Glycopeptides	1	4	1	117649	1,000+	175	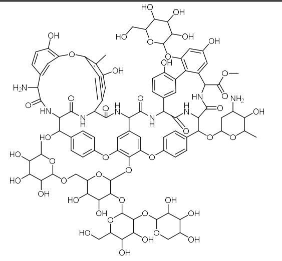	0.23	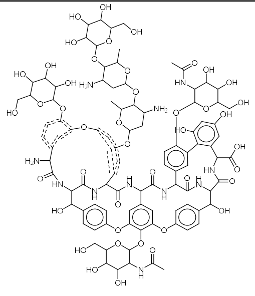	0.259	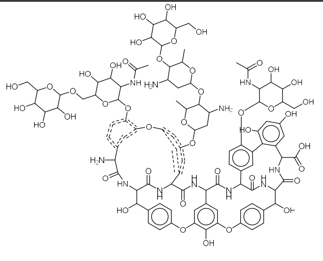
tallysomycin	Glycopeptides	500+	0	5	1612	1,000+	413	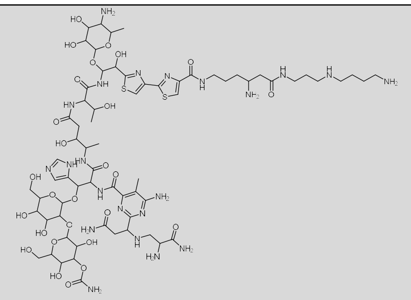	0.175	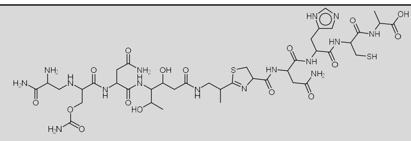	0.228	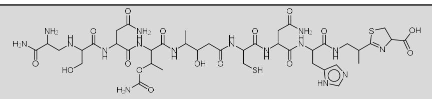
teicoplanin	Glycopeptides	6	1	1	14406	1,000+	156	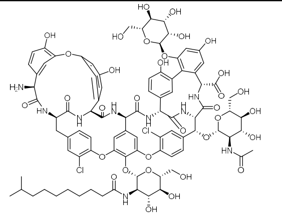	0.278	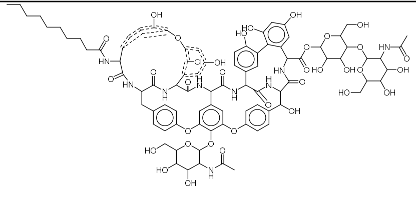	0.293	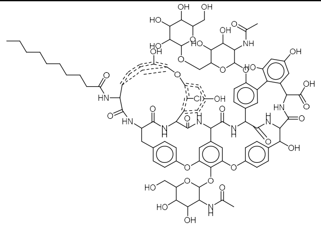
vancomycin	Glycopeptides	1	1	1	216	216	144	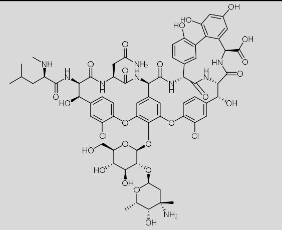	0.299	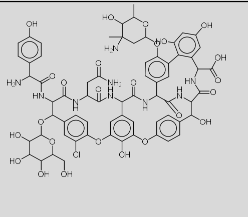	0.326	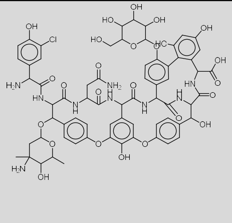

zorbamycin	Glycopeptides	120	0	4	240	960	240	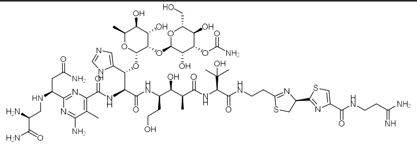	0.172	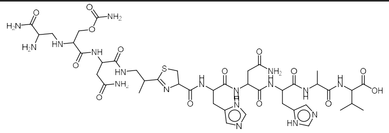	0.202	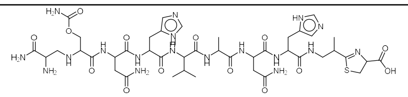
A47934	Lipopeptides	1	0	1	343	343	42	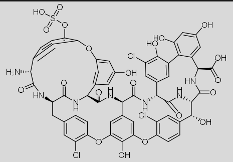	0.181	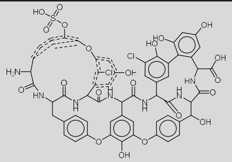	0.2	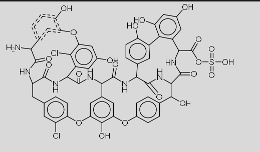
arthrofactin	Lipopeptides	1	0	5	1	5	5	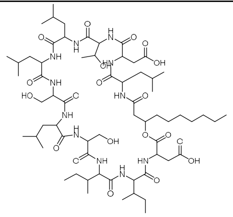	0.4	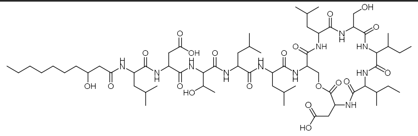	1	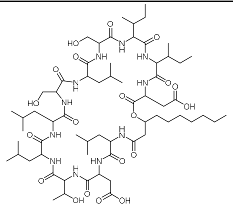
arylomycins	Lipopeptides	1	0	2	1	2	2	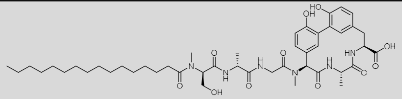	0.312	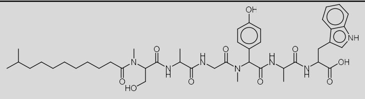	0.312	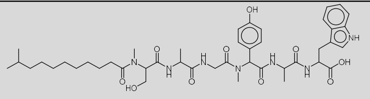
bacillomycin	Lipopeptides	5	0	2	1	2	1	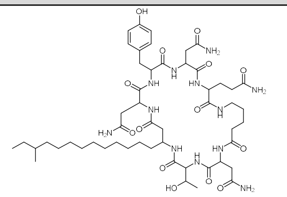	0.144	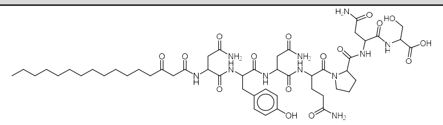	0.144	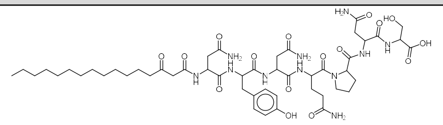
calcium dependent antibiotic	Lipopeptides	1	0	3	1	3	3	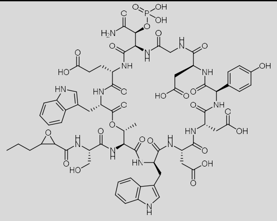	0.293	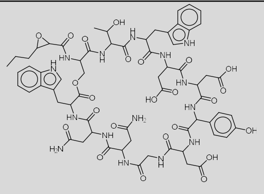	0.416	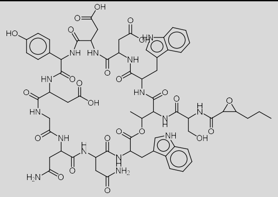
cichofactin	Lipopeptides	1	0	4	1	4	4	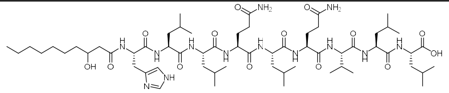	0.344	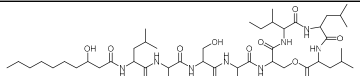	0.504	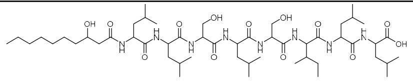
cystomanamide	Lipopeptides	1	0	2	1	2	1	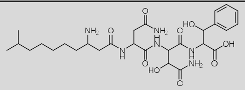	0.197	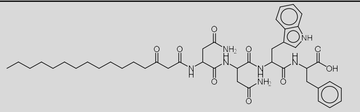	0.197	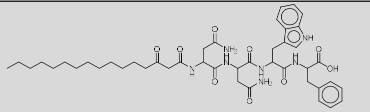
daptomycin	Lipopeptides	1	0	3	2	6	4	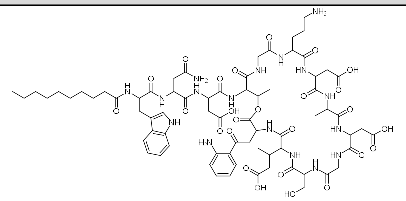	0.636	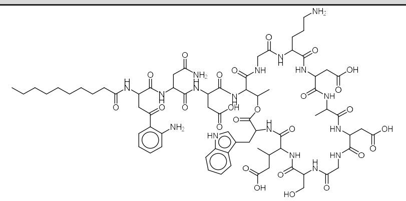	0.645	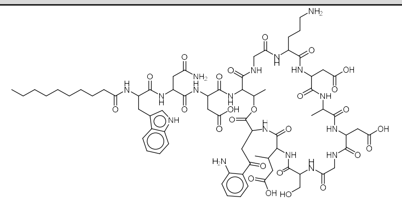
echinocandin	Lipopeptides	1	0	2	1	2	2	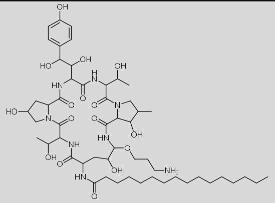	0.098	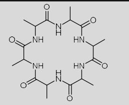	0.098	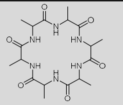

eponemycin	Lipopeptides	1	0	3	1	3	3	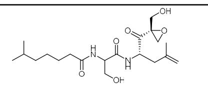	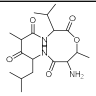	0.096	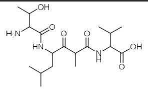	0.172
fengycin	Lipopeptides	1	0	3	1	3	3	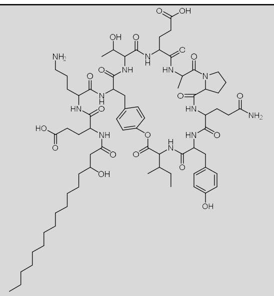	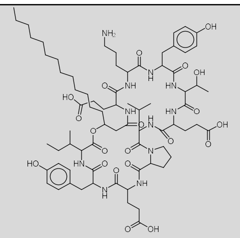	0.375	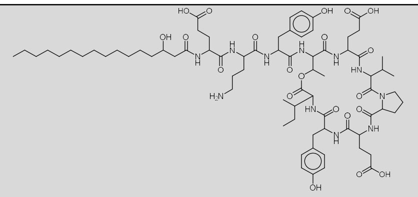	0.519
friulimicin	Lipopeptides	6	0	2	6	12	6	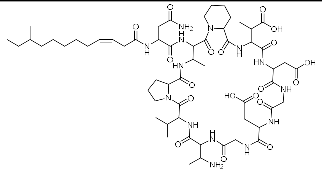	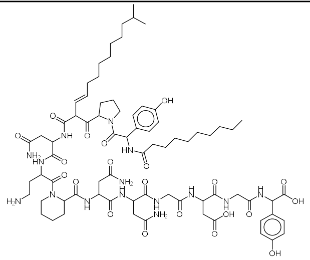	0.238	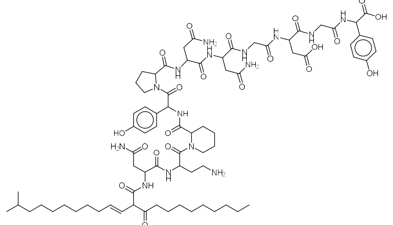	0.252
fusaricidin	Lipopeptides	1	0	3	1	3	3	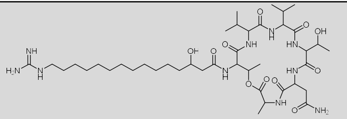	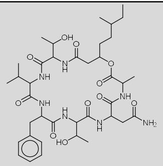	0.42	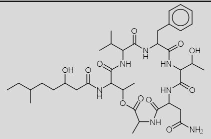	0.638
laspartomycin	Lipopeptides	6	0	2	6	15	9	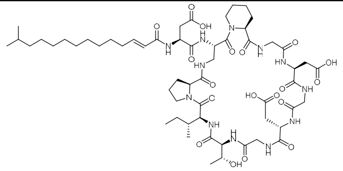	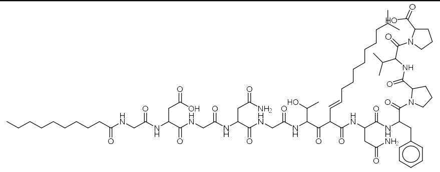	0.25	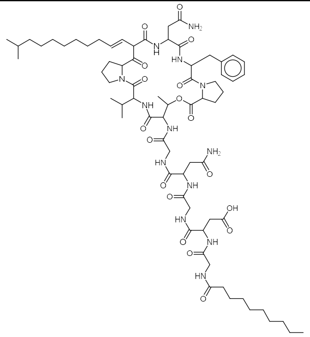	0.326
lichenysin	Lipopeptides	1	0	2	1	2	2	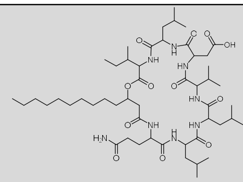	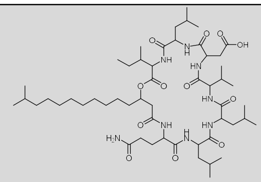	0.89	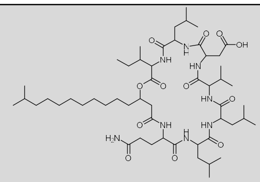	0.89
massetolide	Lipopeptides	1	0	5	1	5	5	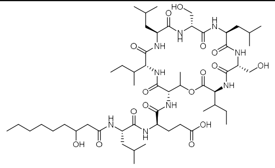	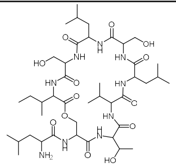	0.377	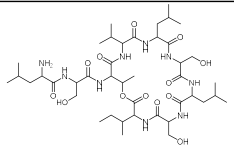	0.559
micropeptin	Lipopeptides	1	0	3	1	3	3	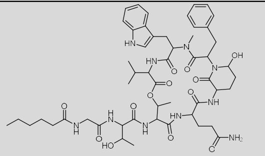	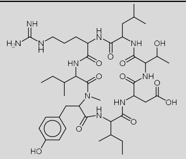	0.224	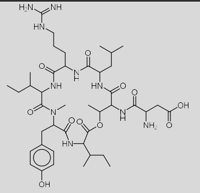	0.294

nostopeptolide	Lipopeptides	1	0	3	1	3	3	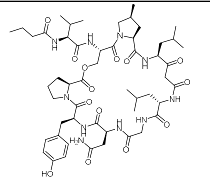	0.483	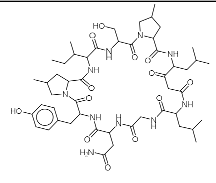	0.551	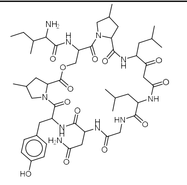
orfamide	Lipopeptides	1	0	5	1	5	5	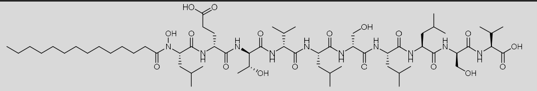	0.252	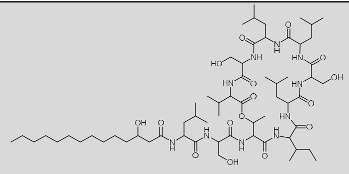	0.504	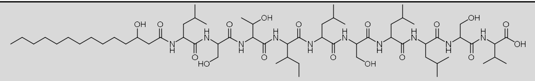
pelgipeptin	Lipopeptides	1	0	1	1	1	1	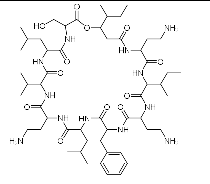	0.118	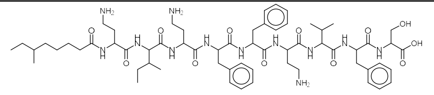	0.118	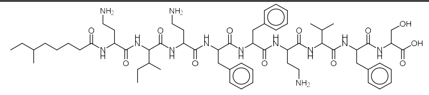
plipastatin	Lipopeptides	1	0	3	1	3	3	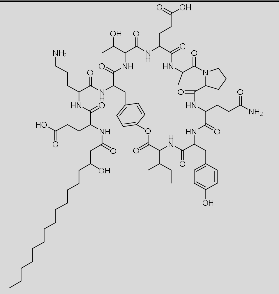	0.375	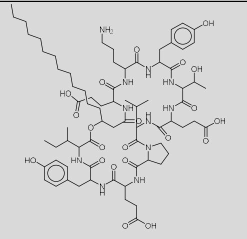	0.519	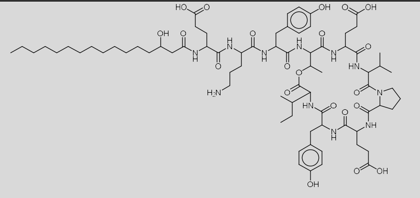
polymyxin	Lipopeptides	2	0	3	2	6	6	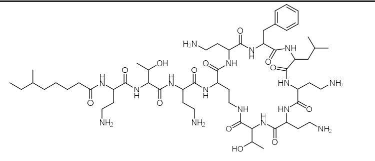	0.436	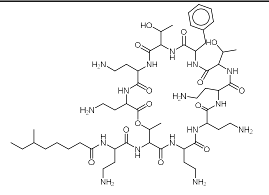	0.512	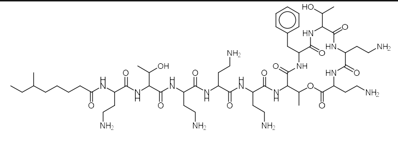
putisolvin	Lipopeptides	1	0	5	1	5	5	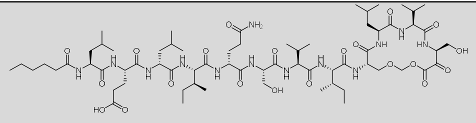	0.519	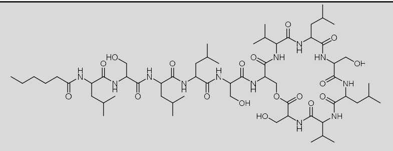	0.597	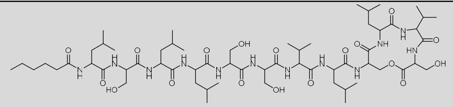
serobactin	Lipopeptides	1	0	4	1	4	3	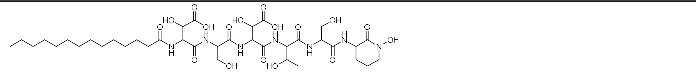	0.203	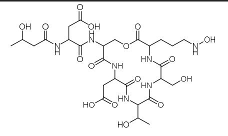	0.274	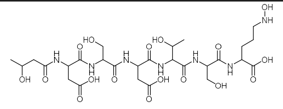
skyllamycin	Lipopeptides	1	0	5	1	5	5	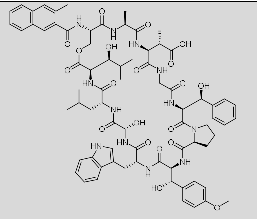	0.169	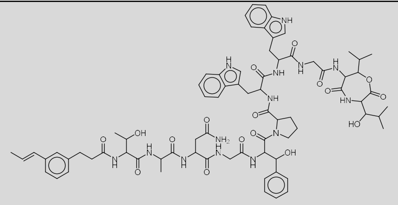	0.279	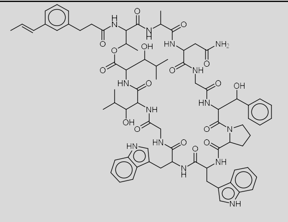
surfactin	Lipopeptides	1	0	2	1	2	2	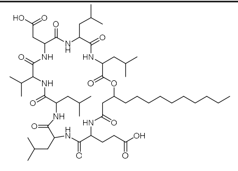	0.872	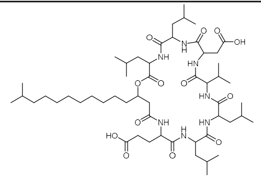	0.872	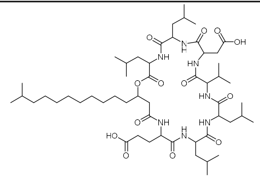

syringomycin	Lipopeptides	1	0	5	1	5	5	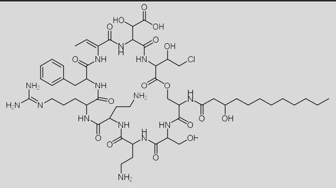	0.397	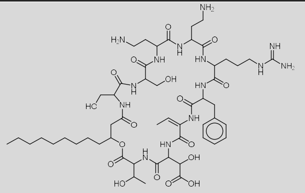	0.609	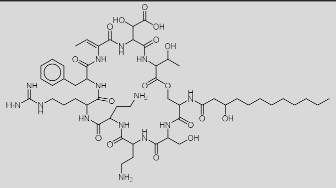
thalassospiramide	Lipopeptides	1	0	1	1	1	1	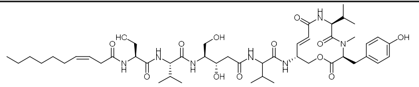	0.262	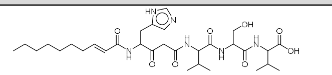	0.262	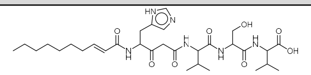
tolaasin	Lipopeptides	1	0	8	1	8	8	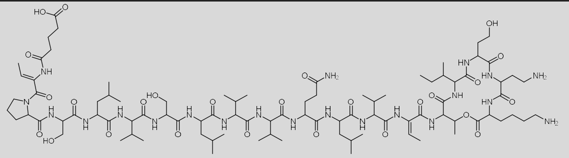	0.412	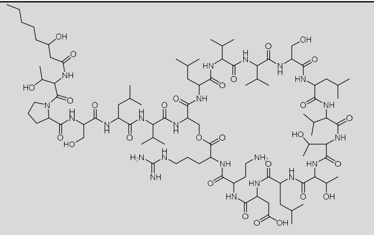	0.509	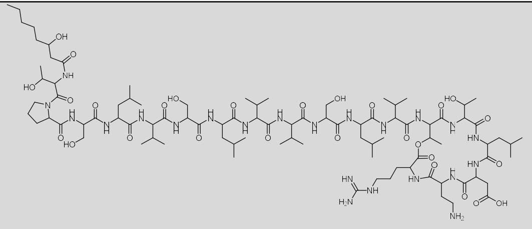
tridecaptin	Lipopeptides	1	0	4	1	4	4	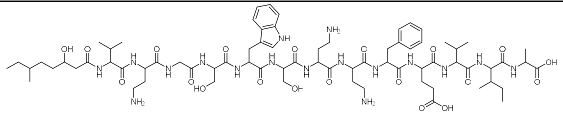	0.391	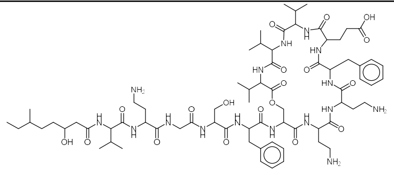	0.556	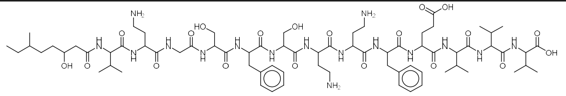
amphotericin	Macrolides	120	3	7	768	1,000+	337	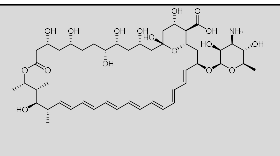	0.195	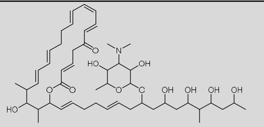	0.267	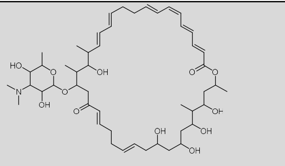
apoptolidin	Macrolides	1	18	6	125	1,000+	60	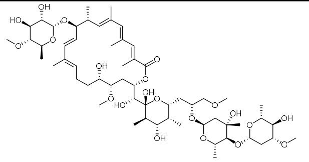	0.308	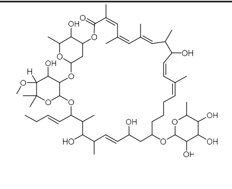	0.387	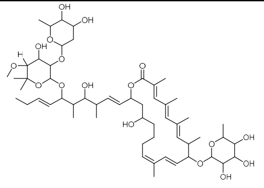
ascomycin	Macrolides	6	0	3	6	18	12	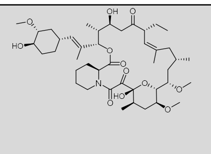	0.221	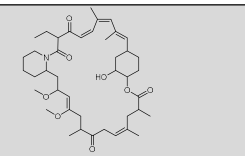	0.307	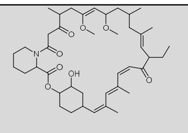
bafilomycin	Macrolides	24	0	8	24	192	42	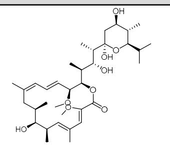	0.263	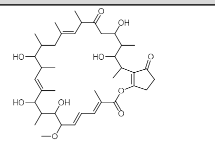	0.463	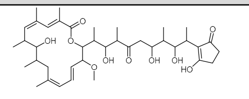
BE-14106	Macrolides	120	0	3	120	360	184	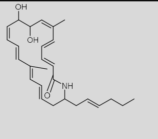	0.38	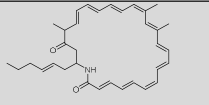	0.634	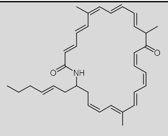
borrelidin	Macrolides	1	0	3	1	3	3	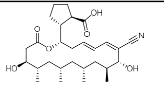	0.198	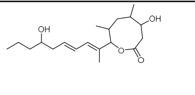	0.337	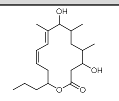
chalcomycin	Macrolides	1	7	3	4	84	4	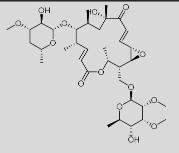	0.229	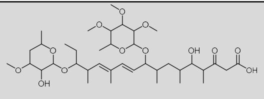	0.244	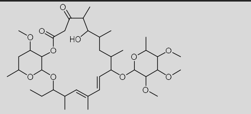

concanamycin A	Macrolides	1	2	7	36	504	180	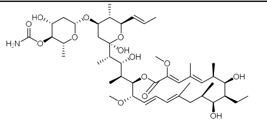	0.436 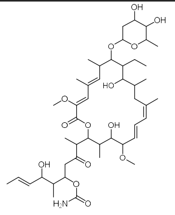	0.559 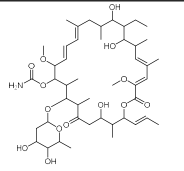
daunorubicin	Macrolides	1	1	1	10	10	2	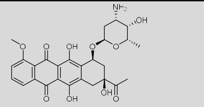	0.357 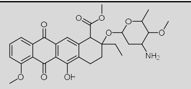	0.357 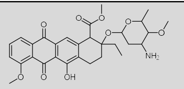
epothilone	Macrolides	1	0	4	1	4	4	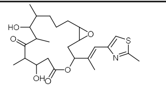	0.258 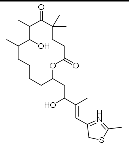	0.322 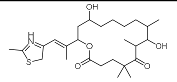
erythromycin	Macrolides	1	5	4	9	180	36	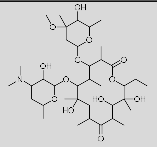	0.259 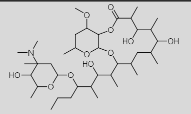	0.327 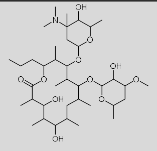
FK506	Macrolides	24	0	3	24	72	48	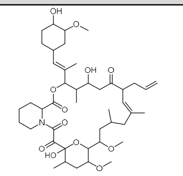	0.2 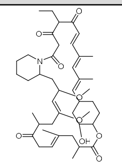	0.265 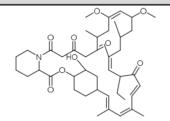
FR-008	Macrolides	24	3	10	186	1,000+	267	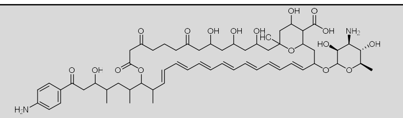	0.181 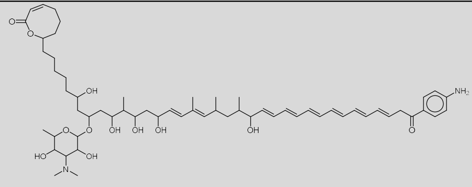	0.228 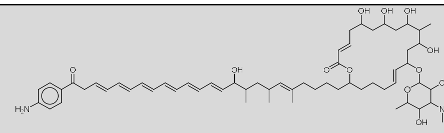
megalomicin	Macrolides	1	44	4	27	1,000+	24	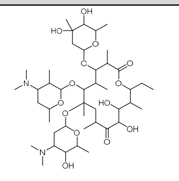	0.225 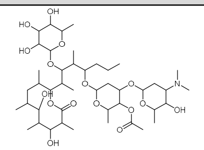	0.254 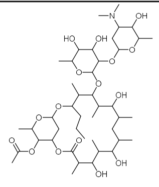
meridamycin	Macrolides	6	0	9	6	54	54	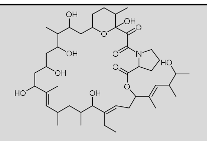	0.286 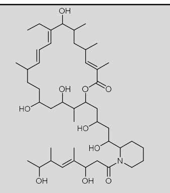	0.653 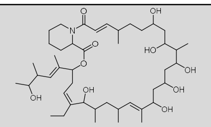
methymycin	Macrolides	1	0	3	1	3	3	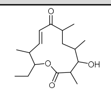	0.233 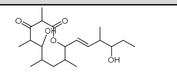	0.4 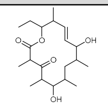
ML-449	Macrolides	120	0	3	120	360	184	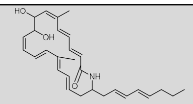	0.319 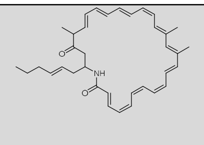	0.529 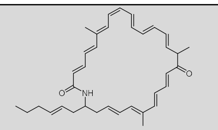
natamycin	Macrolides	24	3	6	132	1,000+	335	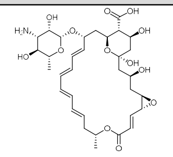	0.218 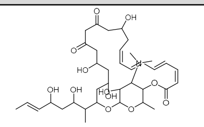	0.362 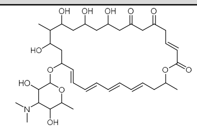

niddamycin	Macrolides	1	0	3	1	3	3	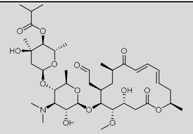	0.183	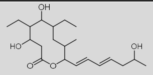	0.254 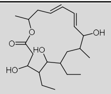
nystatin	Macrolides	120	3	8	888	1,000+	335	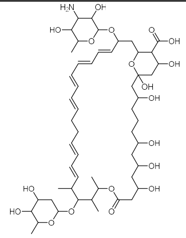	0.243	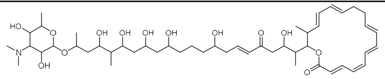	0.348 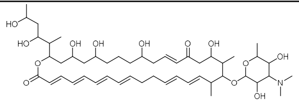
oligomycin	Macrolides	120	0	8	120	960	960	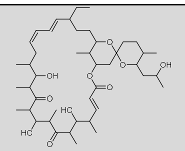	0.224	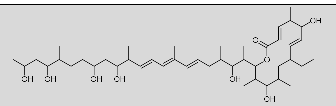	0.399 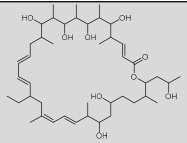
pikromycin	Macrolides	1	1	3	2	6	6	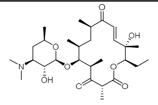	0.32	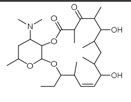	0.404 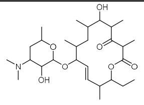
pimaricin	Macrolides	24	3	6	132	1,000+	335	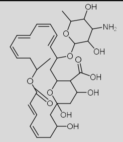	0.232	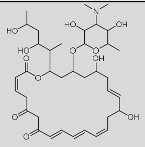	0.372 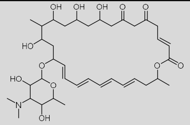
rapamycin	Macrolides	6	0	3	6	18	12	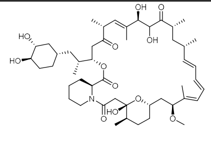	0.221	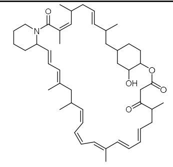	0.286 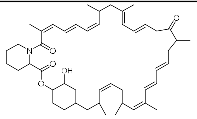
rimocidin	Macrolides	1	3	1	1	3	1	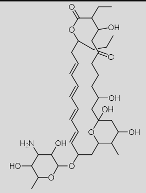	0.058	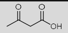	0.058 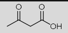
tetramycin	Macrolides	24	3	6	108	1,000+	188	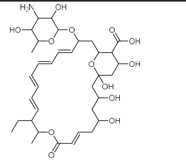	0.189	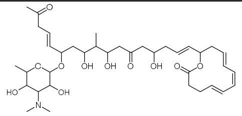	0.278 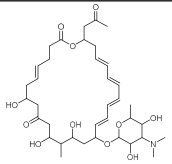
thuggacin	Macrolides	1	0	6	1	6	6	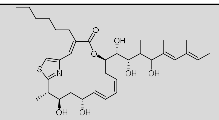	0.226	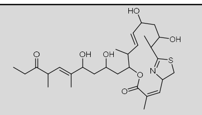	0.244 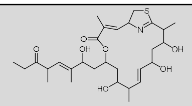
tylactone	Macrolides	1	0	3	1	3	3	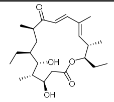	0.337	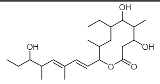	0.658 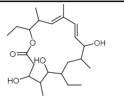
vicenistatin	Macrolides	6	1	2	6	12	4	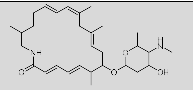	0.266	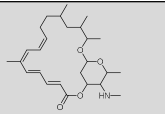	0.294 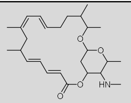

bacillaene	Trans AT	8	0	5	1	5	5	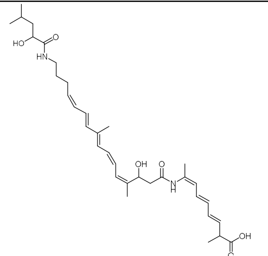	0.132	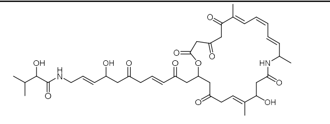	0.199	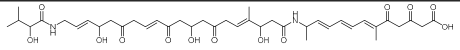
bryostatin	Trans AT	7	0	5	1	5	5	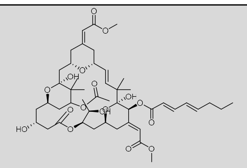	0.161	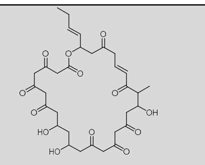	0.198	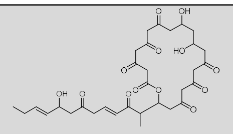
chivosazol	Trans AT	6	0	6	1	6	6	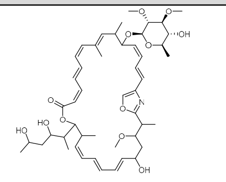	0.141	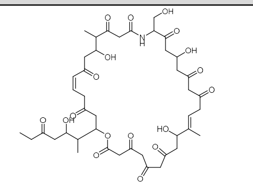	0.148	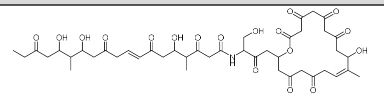
difficidin	Trans AT	11	0	5	1	5	5	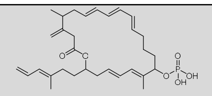	0.152	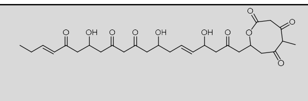	0.169	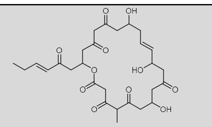
disorazole	Trans AT	4	0	3	1	3	3	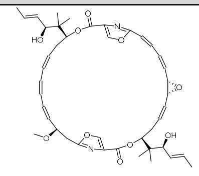	0.131	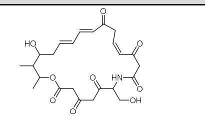	0.154	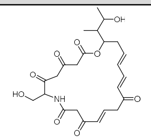
elansolid	Trans AT	11	0	6	1	6	5	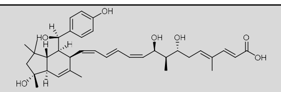	0.171	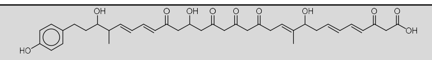	0.205	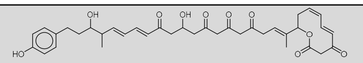
etnangien	Trans AT	10	0	8	1	8	8	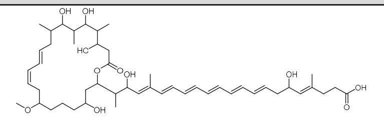	0.236	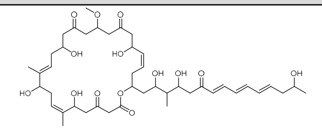	0.271	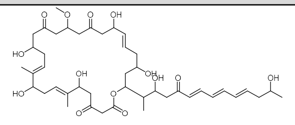
iturin	Trans AT	5	0	2	1	2	1	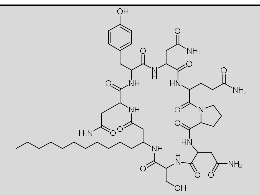	0.178	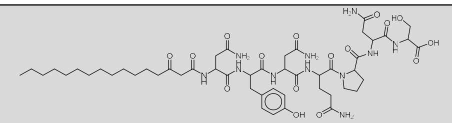	0.178	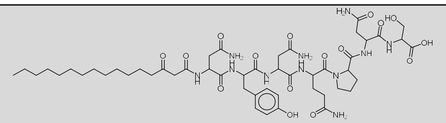
kirromycin	Trans AT	10	0	5	1	5	5	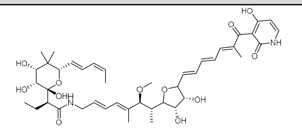	0.155	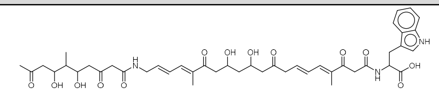	0.193	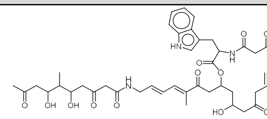
lankacidin	Trans AT	5	0	3	1	3	3	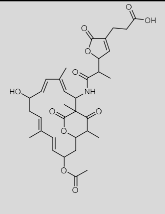	0.078	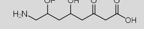	0.098	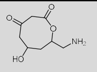

leinamycin	Trans AT	3	0	3	1	3	3	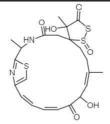	0.113 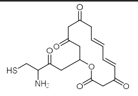	0.151	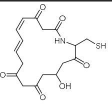
macrolactin	Trans AT	6	0	4	1	4	4	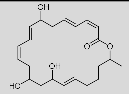	0.136 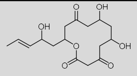	0.143	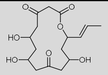
mupirocin	Trans AT	6	0	3	1	3	3	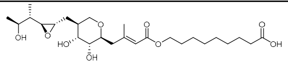	0.12 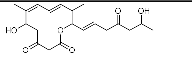	0.124	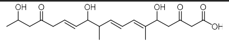
myxovirescin	Trans AT	6	0	3	1	3	3	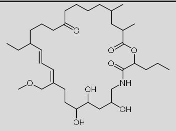	0.094 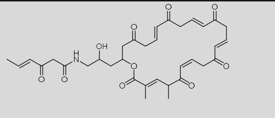	0.121	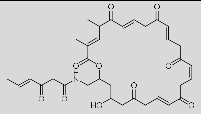
oxazolomycin	Trans AT	8	0	3	1	3	3	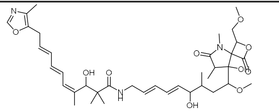	0.157 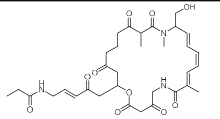	0.173	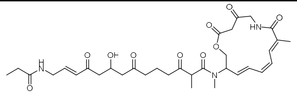
pederin	Trans AT	3	0	5	1	5	5	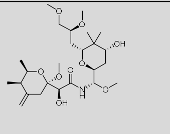	0.128 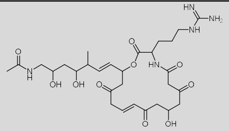	0.151	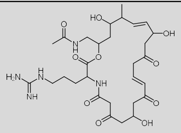
psymberin	Trans AT	2	0	6	1	6	6	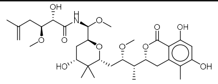	0.171 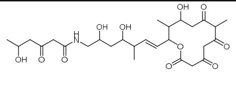	0.193	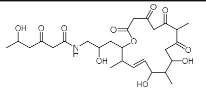
rhizoxin	Trans AT	10	0	5	1	5	5	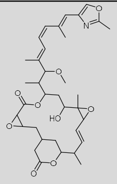	0.165 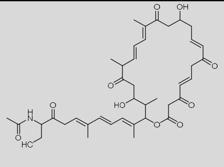	0.199	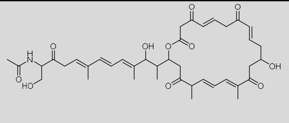
thailandamide	Trans AT	10	0	6	1	6	1	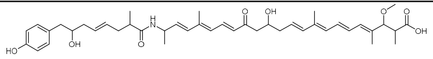	0.175 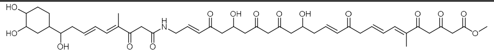	0.175	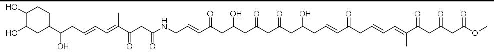
virginiamycin	Trans AT	34	0	4	6	28	28	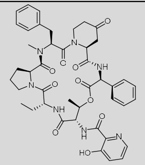	0.153 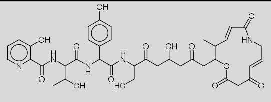	0.191	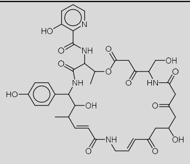
A54145	Type II	1	0	5	1	5	4	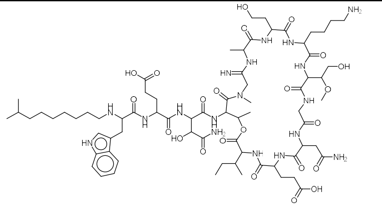	0.478 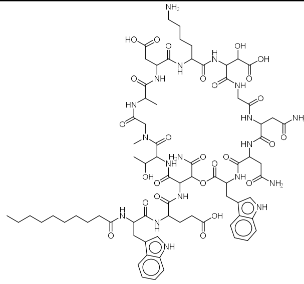	0.571	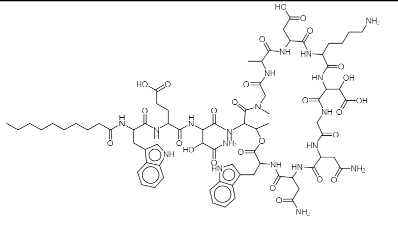
aclacinomycin A	Type II	1	4	1	100	400	9	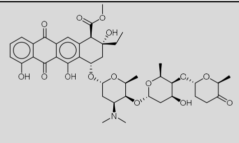	0.223 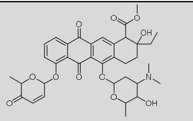	0.258	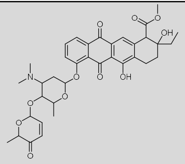

actinorhodin	Type II	1	42	1	2000	1,000+	4	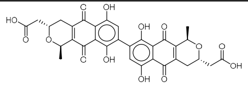	0.179	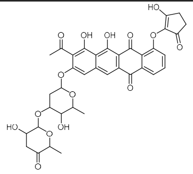	0.182	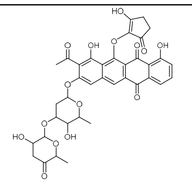
alnumycin	Type II	1	0	1	1	1	1	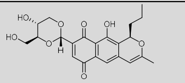	0.114	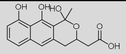	0.114	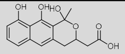
aranciamycin	Type II	1	1	1	10	10	4	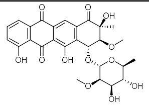	0.487	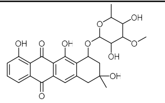	0.491	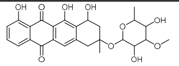
BE-7585A	Type II	1	1	1	10	10	3	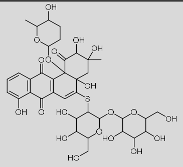	0.22	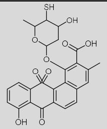	0.23	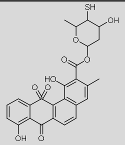
benastatin	Type II	1	0	1	1	1	1	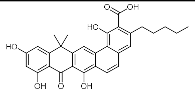	0.37	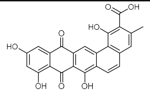	0.37	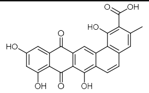
chlortetracycline	Type II	1	0	1	8	8	4	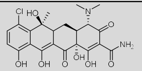	0.207	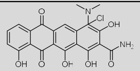	0.227	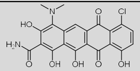
chromomycin	Type II	1	100+	1	10000	1,000+	4	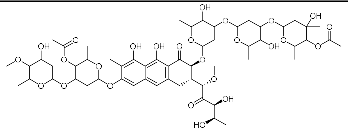	0.201	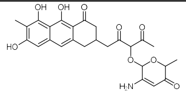	0.254	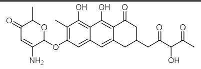
chrysomycin	Type II	1	2	1	10	20	4	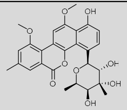	0.295	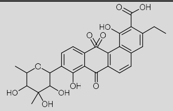	0.308	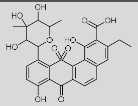
cosmomycin	Type II	1	2	1	100	200	1	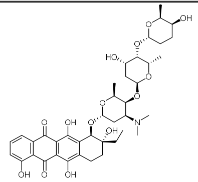	0.1	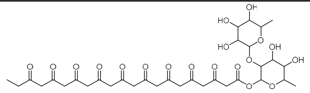	0.1	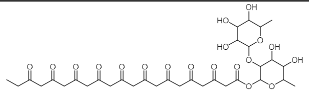
dactylocycline	Type II	1	1	1	72	72	20	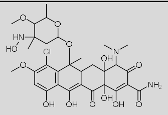	0.215	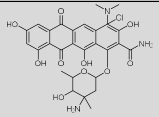	0.245	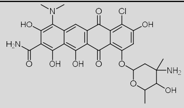
elloramycin	Type II	1	1	1	10	10	5	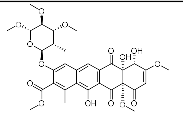	0.257	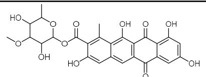	0.304	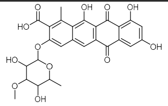
enterocin	Type II	1	0	1	1	1	1	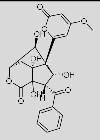	0.223	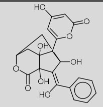	0.223	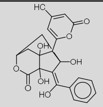
erdacin	Type II	1	0	1	1	1	1	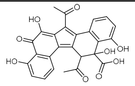	0.208	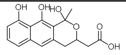	0.208	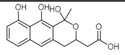

FD-594	Type II	1	7	1	169	1,000+	16	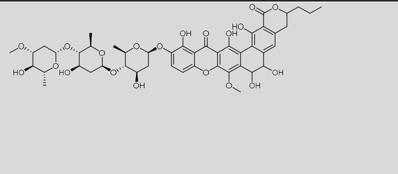	0.22	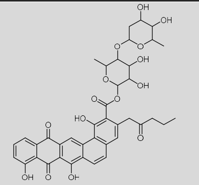	0.258	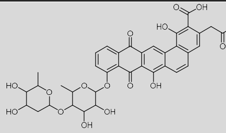
fredericamycin	Type II	1	0	1	1	1	1	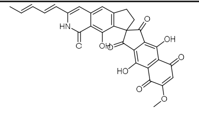	0.167	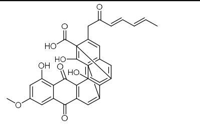	0.167	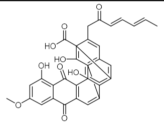
frenolicin	Type II	1	0	1	1	1	1	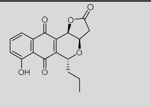	0.333	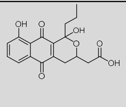	0.333	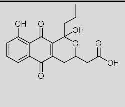
gilvocarcin	Type II	1	1	1	10	10	3	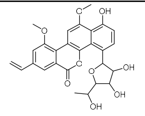	0.205	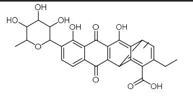	0.212	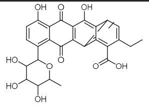
granaticin	Type II	1	4	1	8	32	3	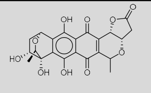	0.176	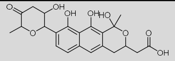	0.177	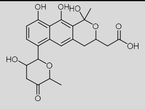
griseorhodin	Type II	1	0	1	1	1	1	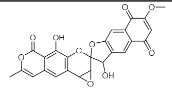	0.11	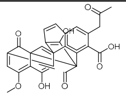	0.11	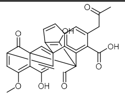
Hedamycin	Type II	1	23	1	100	1,000+	8	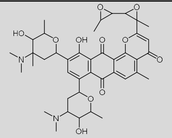	0.177	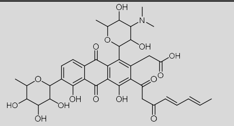	0.2	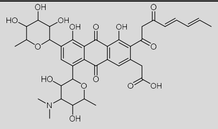
jadomycin	Type II	1	3	1	10	30	3	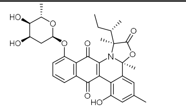	0.295	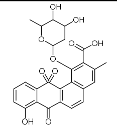	0.36	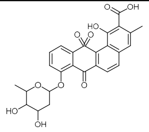
kinamycin	Type II	1	0	1	1	1	1	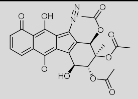	0.092	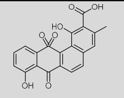	0.092	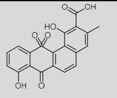
landomycin	Type II	1	100+	1	10000	1,000+	5	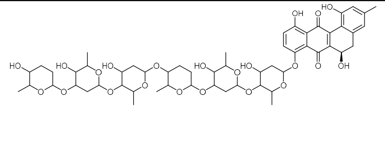	0.155	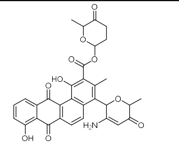	0.203	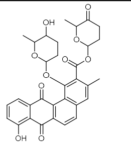
lysolipin	Type II	1	3	1	156	468	20	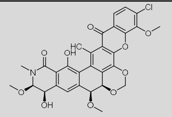	0.16	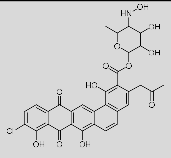	0.179	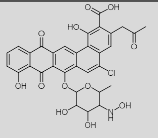
medermycin	Type II	1	1	1	8	8	3	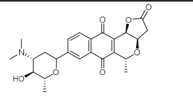	0.264	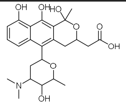	0.279	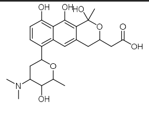
mithramycin	Type II	1	100+	1	200000	1,000+	3	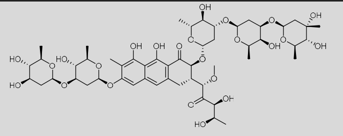	0.148	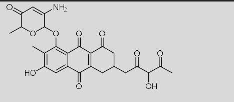	0.158	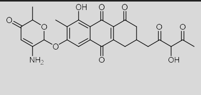

naphthocyclinone	Type II	1	0	1	1	1	1	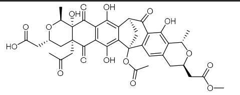	0.222	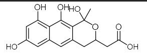	0.222	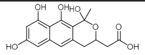
nogalamycin	Type II	1	100+	1	1000	1,000+	3	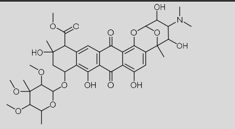	0.373	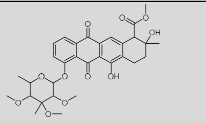	0.373	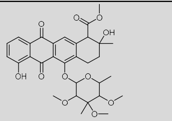
polyketomycin	Type II	1	12	1	2000	1,000+	16	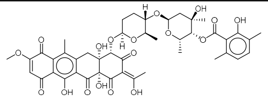	0.156	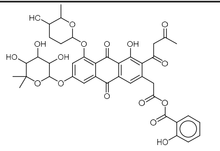	0.165	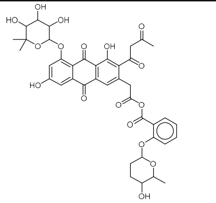
pradimicin	Type II	1	3	1	144	432	10	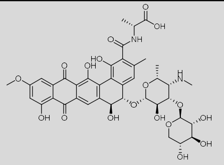	0.279	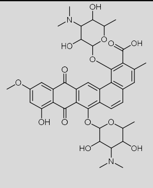	0.287	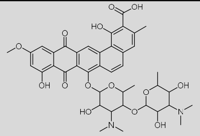
R1128	Type II	1	0	1	1	1	1	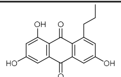	0.149	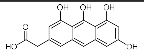	0.149	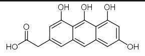
ravidomycin	Type II	1	2	1	10	20	3	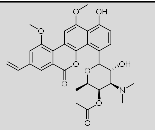	0.275	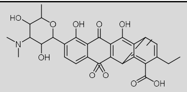	0.295	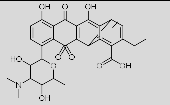
resistomycin	Type II	1	0	1	1	1	1	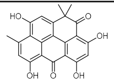	0.055	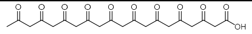	0.055	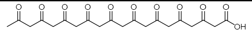
rubrinomycin	Type II	1	0	1	1	1	1	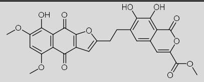	0.075	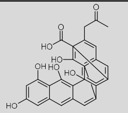	0.075	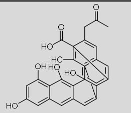
saquayamycin	Type II	1	100+	1	1E+06	1,000+	2	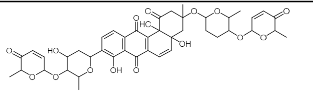	0.218	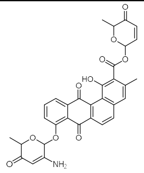	0.218	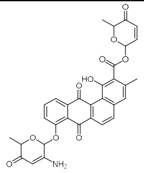
Sch 47554	Type II	1	91	1	1000	1,000+	2	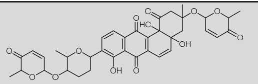	0.236	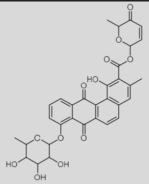	0.236	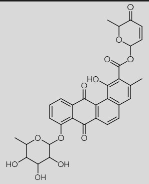
SF2575	Type II	1	2	1	9	18	4	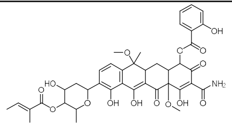	0.198	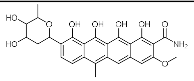	0.207	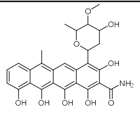
simocyclinone	Type II	1	2	1	1800	1,000+	9	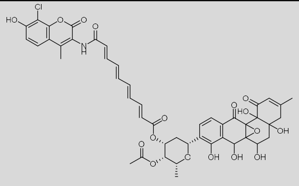	0.171	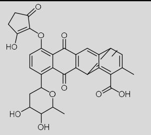	0.214	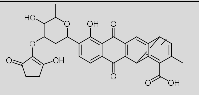

steffimycin	Type II	1	1	1	10	10	4	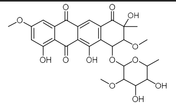	0.323	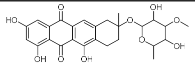	0.385	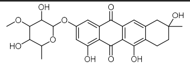
tetracycline	Type II	1	0	1	1	1	1	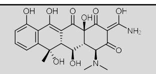	0.214	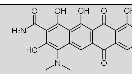	0.214	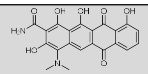
urdamycin	Type II	1	100+	1	10000	1,000+	5	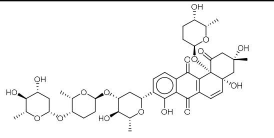	0.141	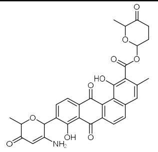	0.18	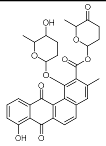
xantholipin	Type II	1	3	1	156	468	20	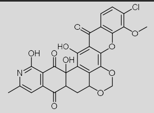	0.17	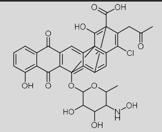	0.194	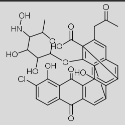
