# Supplementary material for: Genomes to natural products PRediction Informatics for Secondary Metabolomes (PRISM)
Source: Nucleic Acids Res. 2015 Oct 5;43(20):9645–62. doi: 10.1093/nar/gkv1012 (PMC4787774; doi:10.1093/nar/gkv1012)
Supplement: SUPPLEMENTARY DATA [file supp_gkv1012_nar-01872-z-2015-File016.pdf]

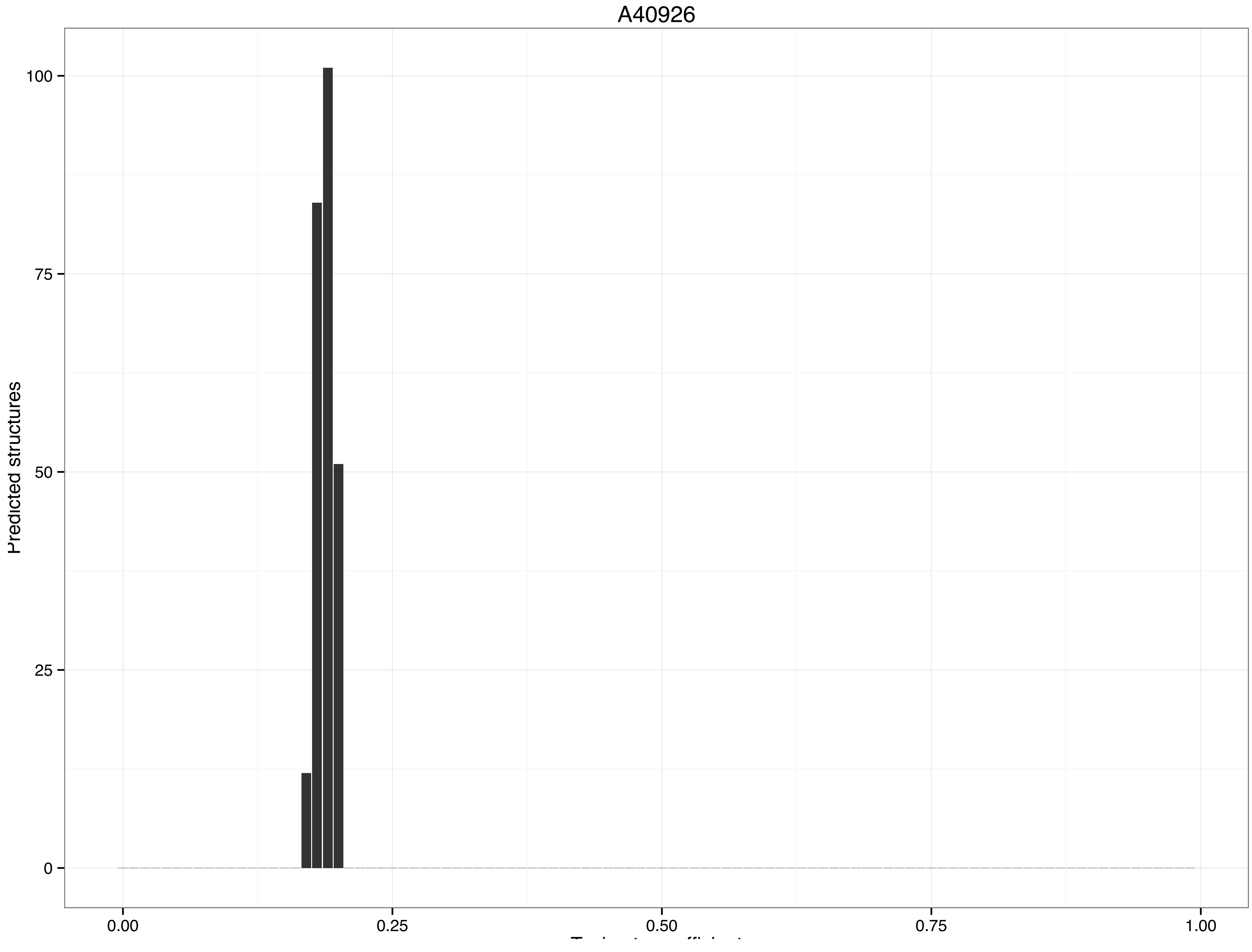
A40926
Predicted structures
100
75
50
25
0
0.00
0.25
0.50
0.75
1.00

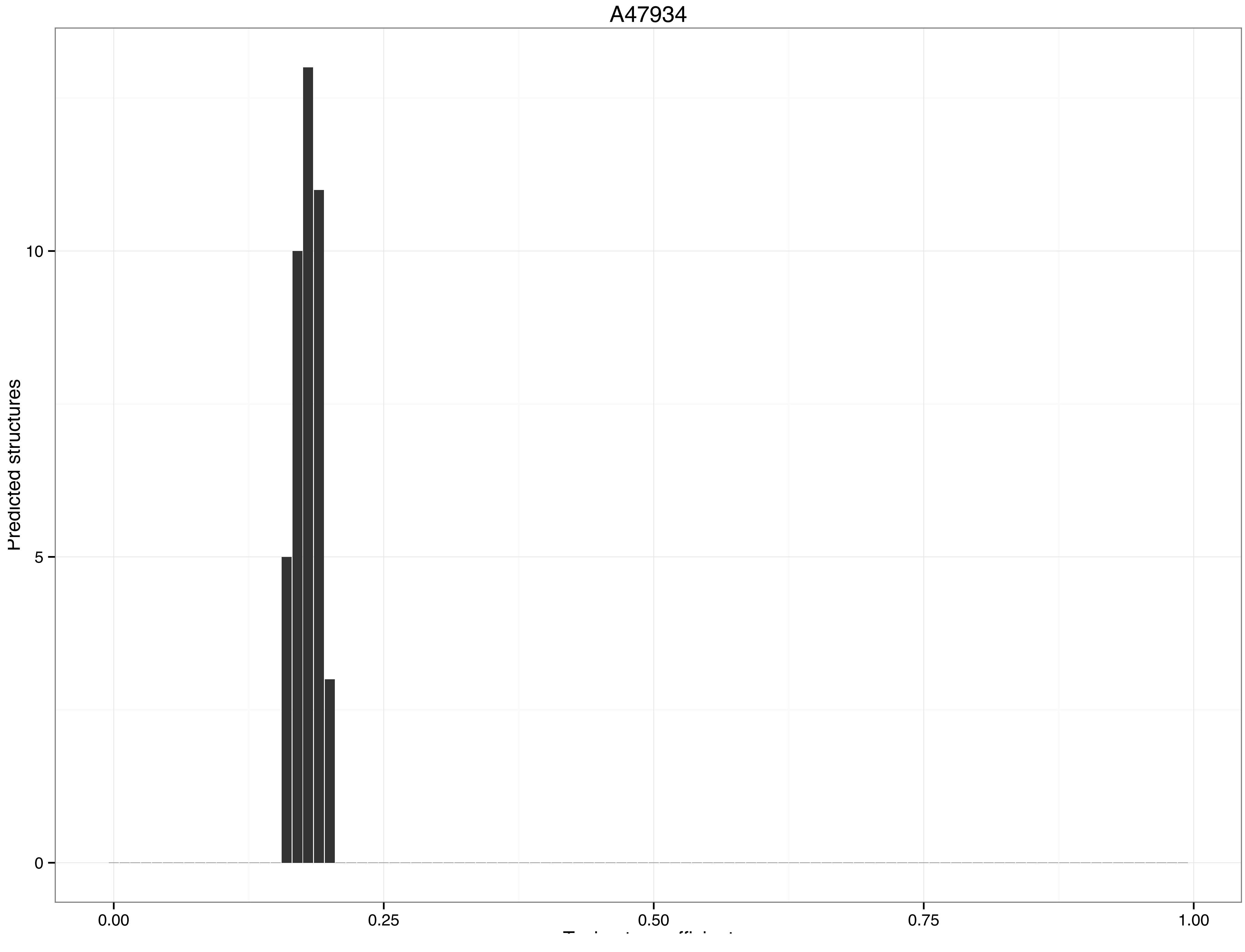
A47934
Predicted structures
10
5
0
0.00
0.25
0.50
0.75
1.00

Amphotericin

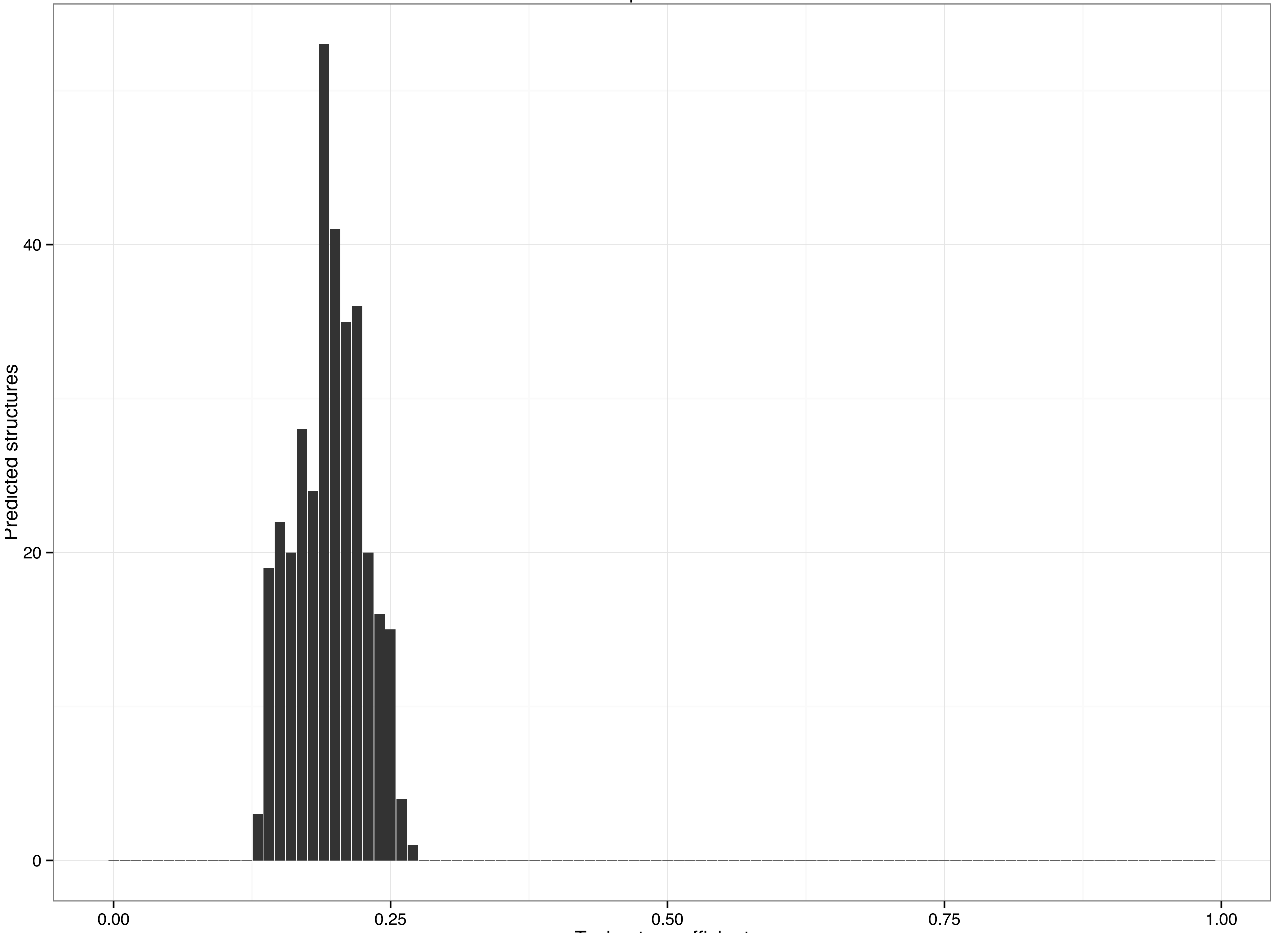

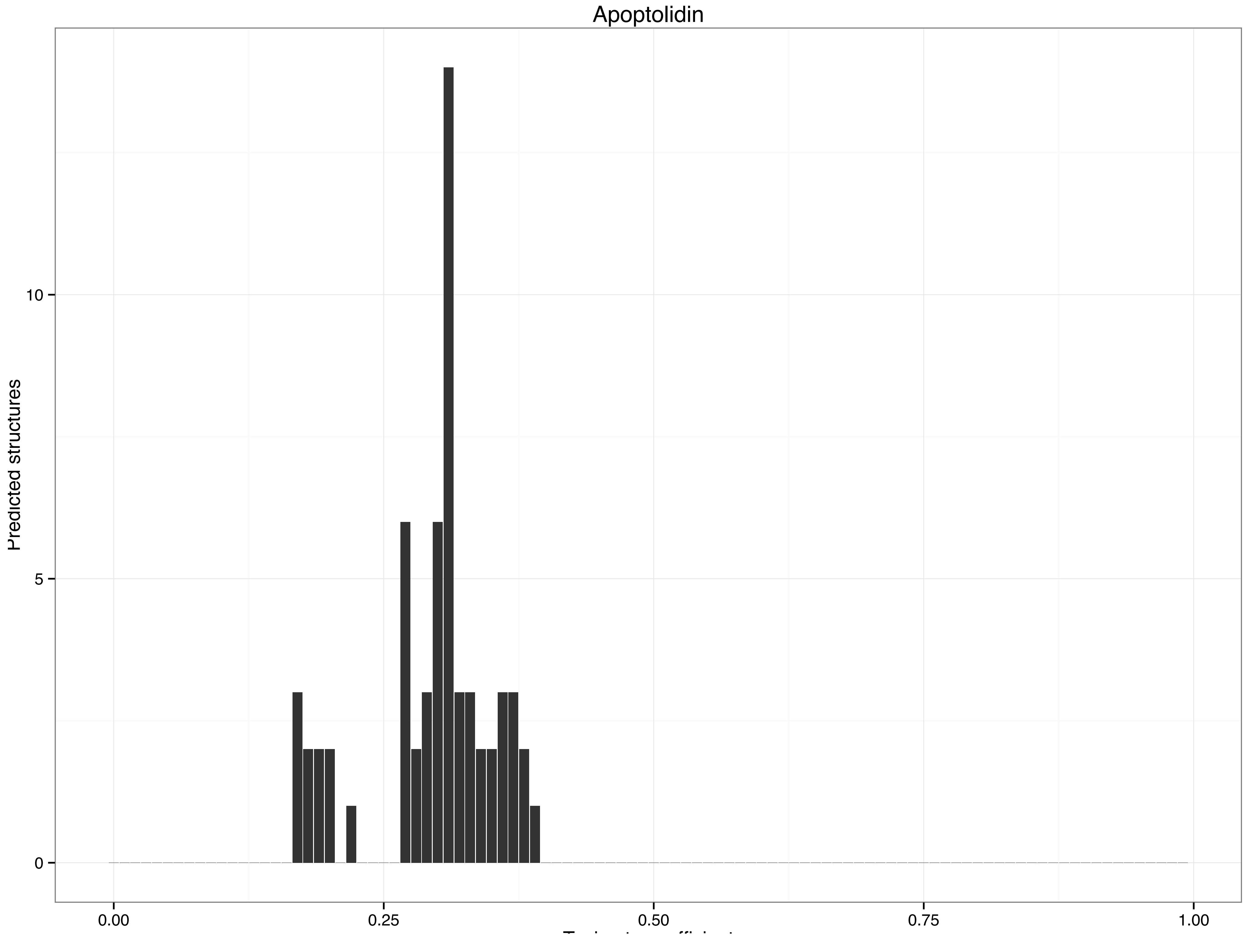

Apoptolidin
Predicted structures
0
5
10
0.00
0.25
0.50
0.75
1.00

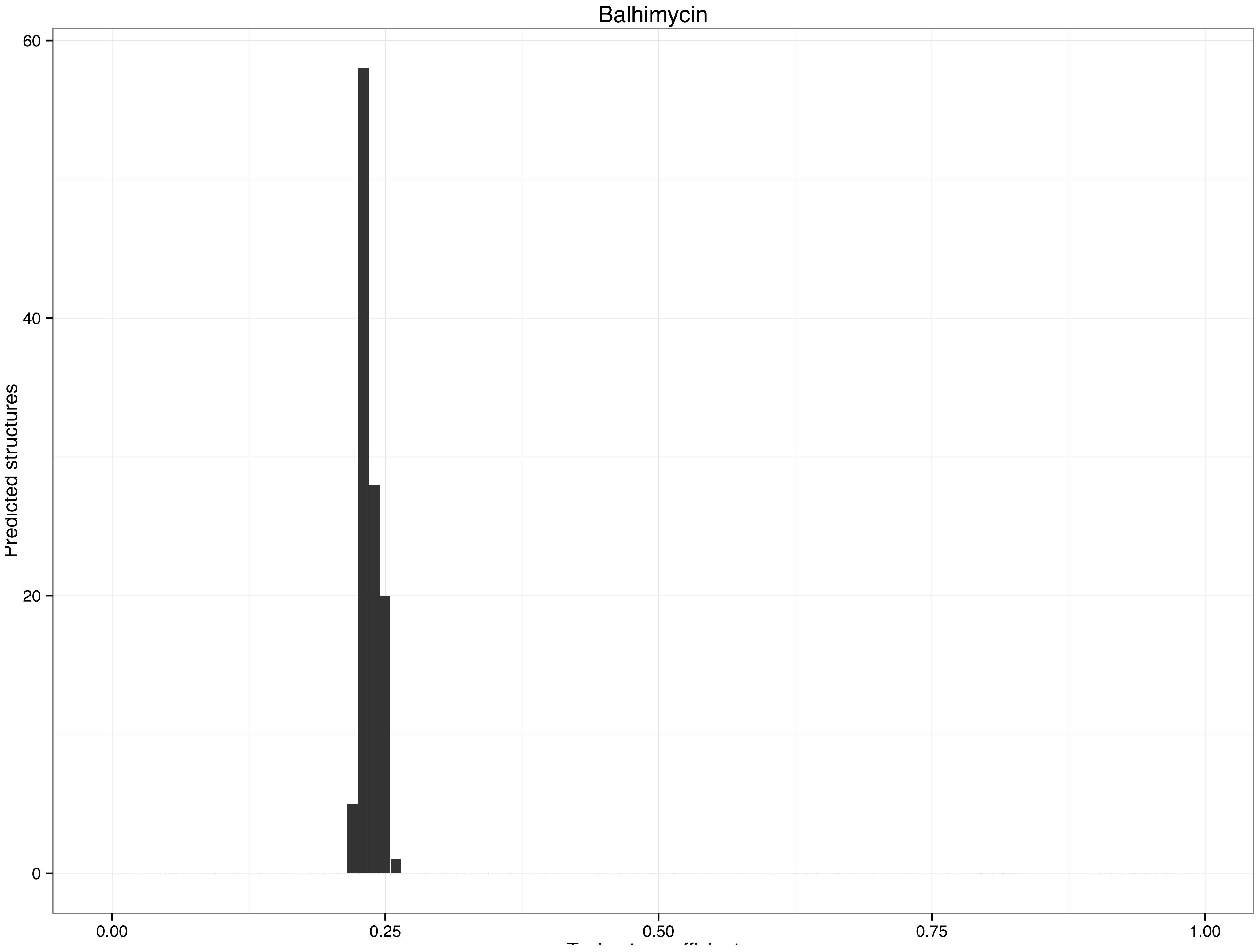
Balhimycin
Predicted structures
0
20
40
60
0.00
0.25
0.50
0.75
1.00

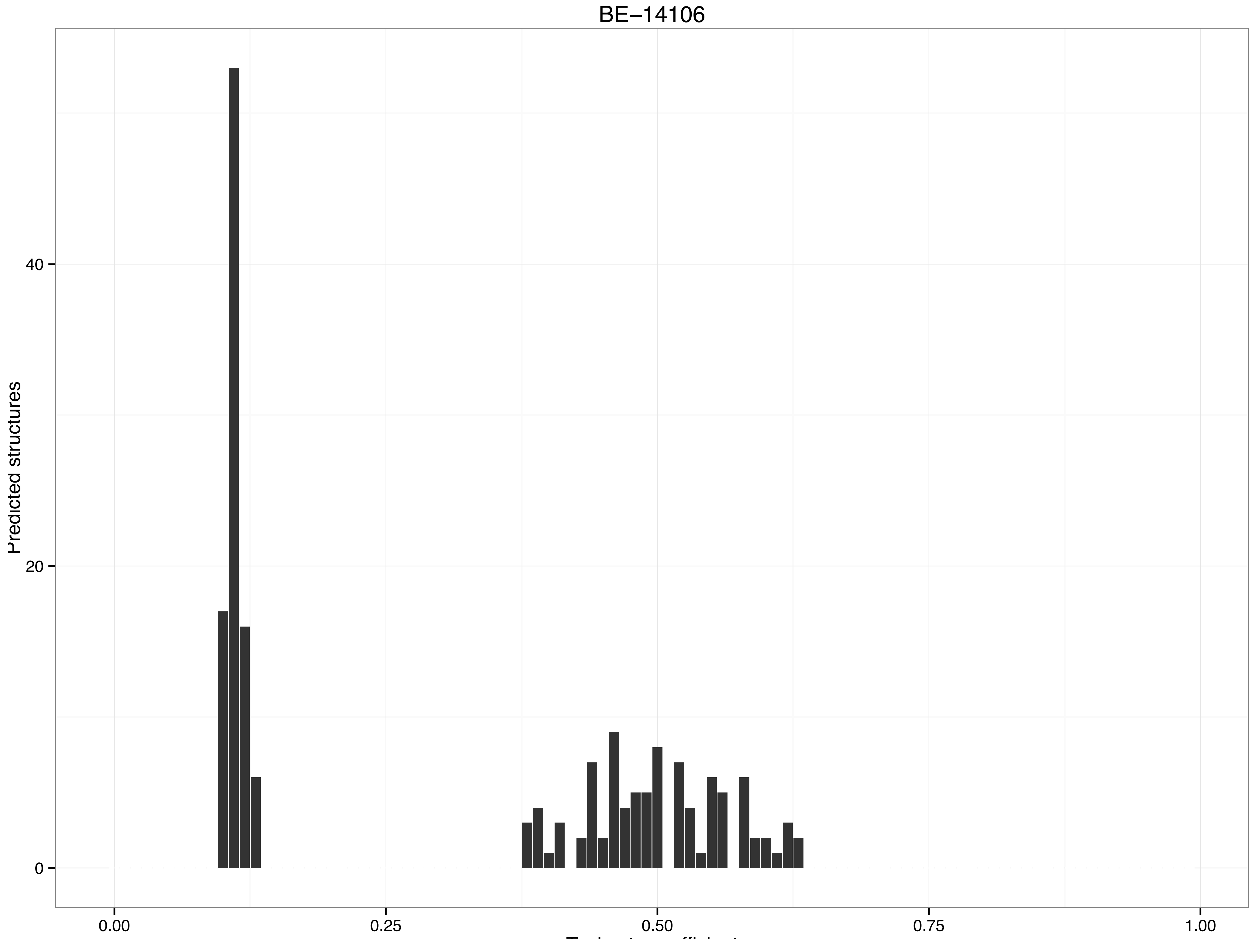
BE−14106
Predicted structures
0
20
40
0.00
0.25
0.50
0.75
1.00

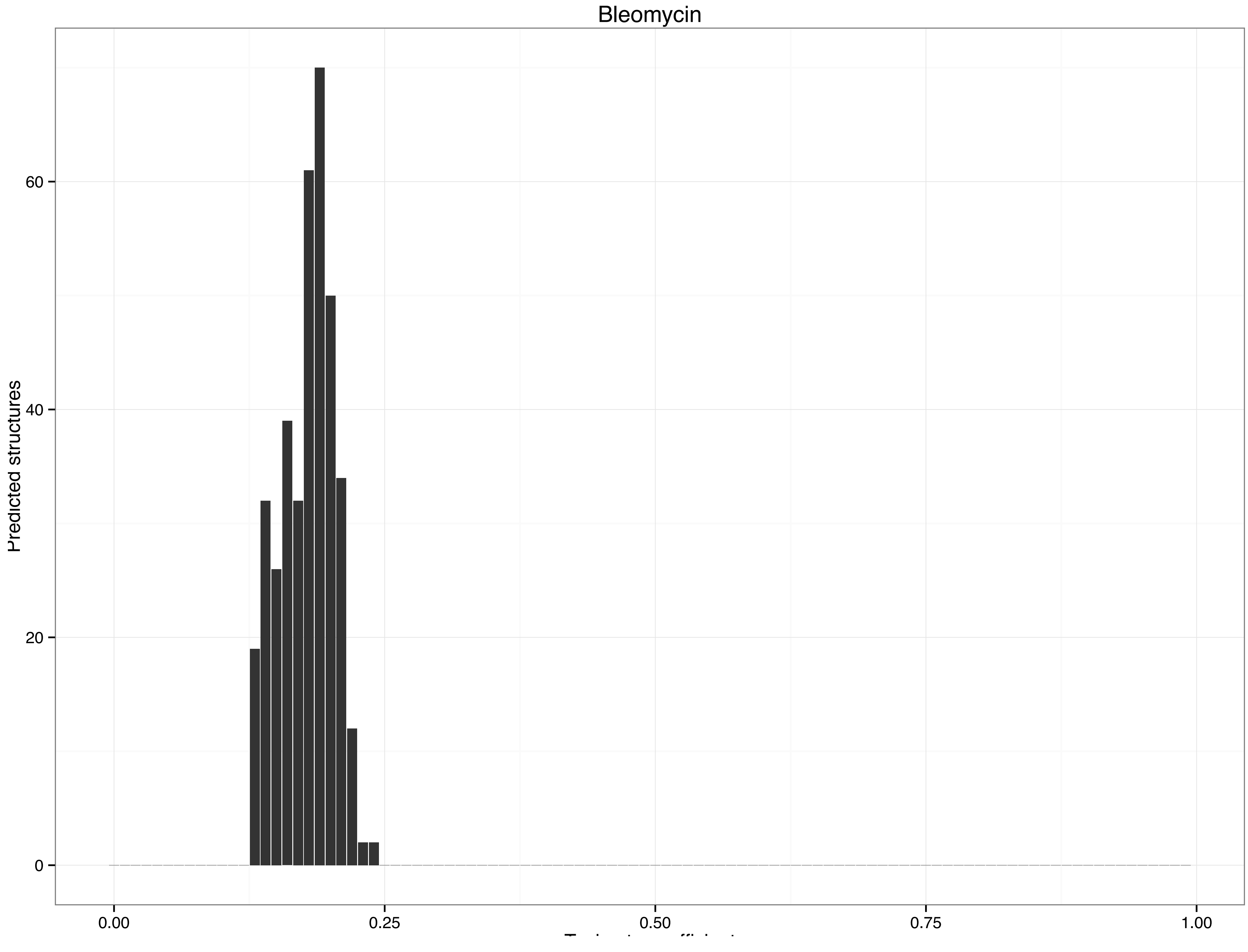
Bleomycin
Predicted structures
0
20
40
60
0.00
0.25
0.50
0.75
1.00

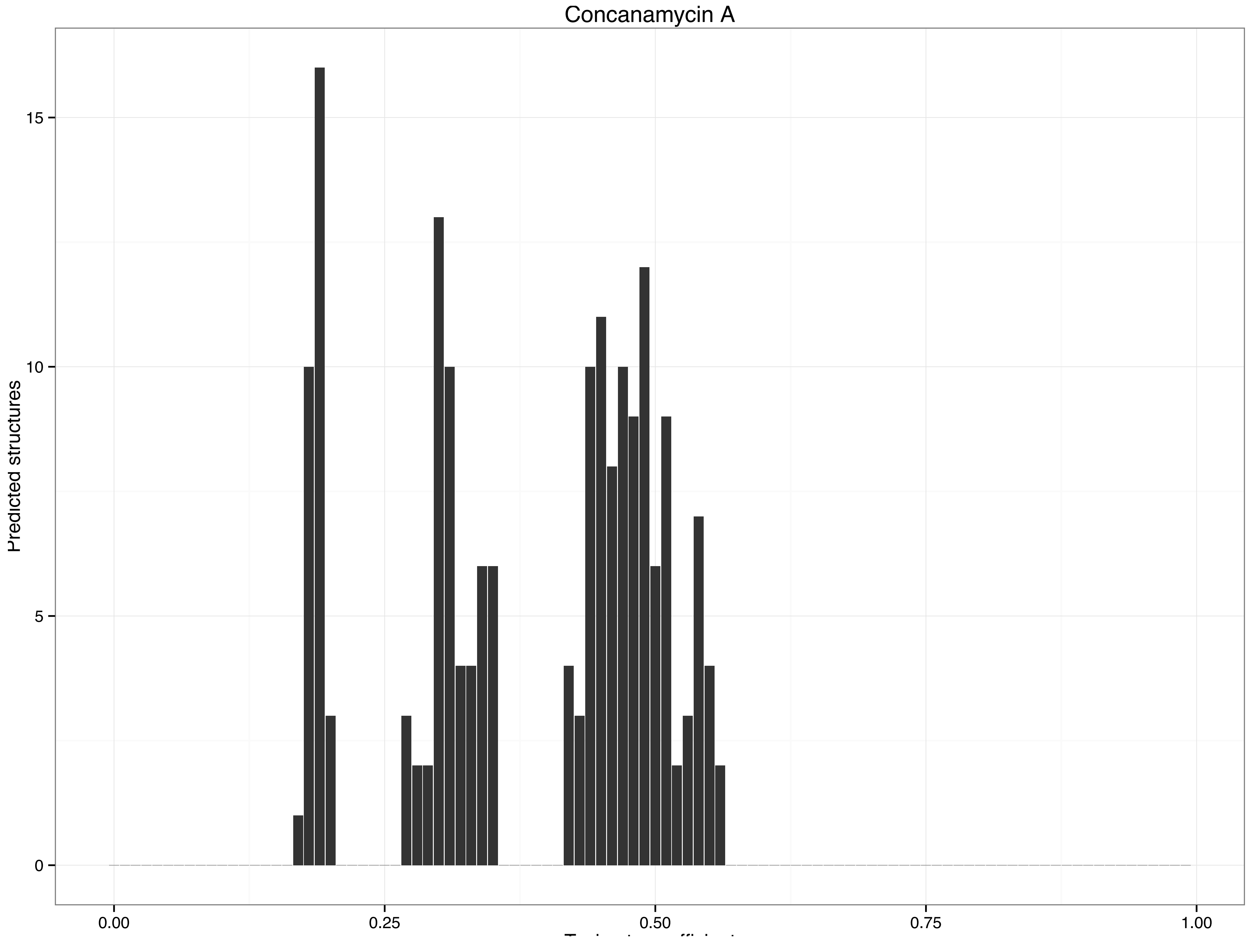

Concanamycin A
Predicted structures
15
10
5
0
0.00
0.25
0.50
0.75
1.00

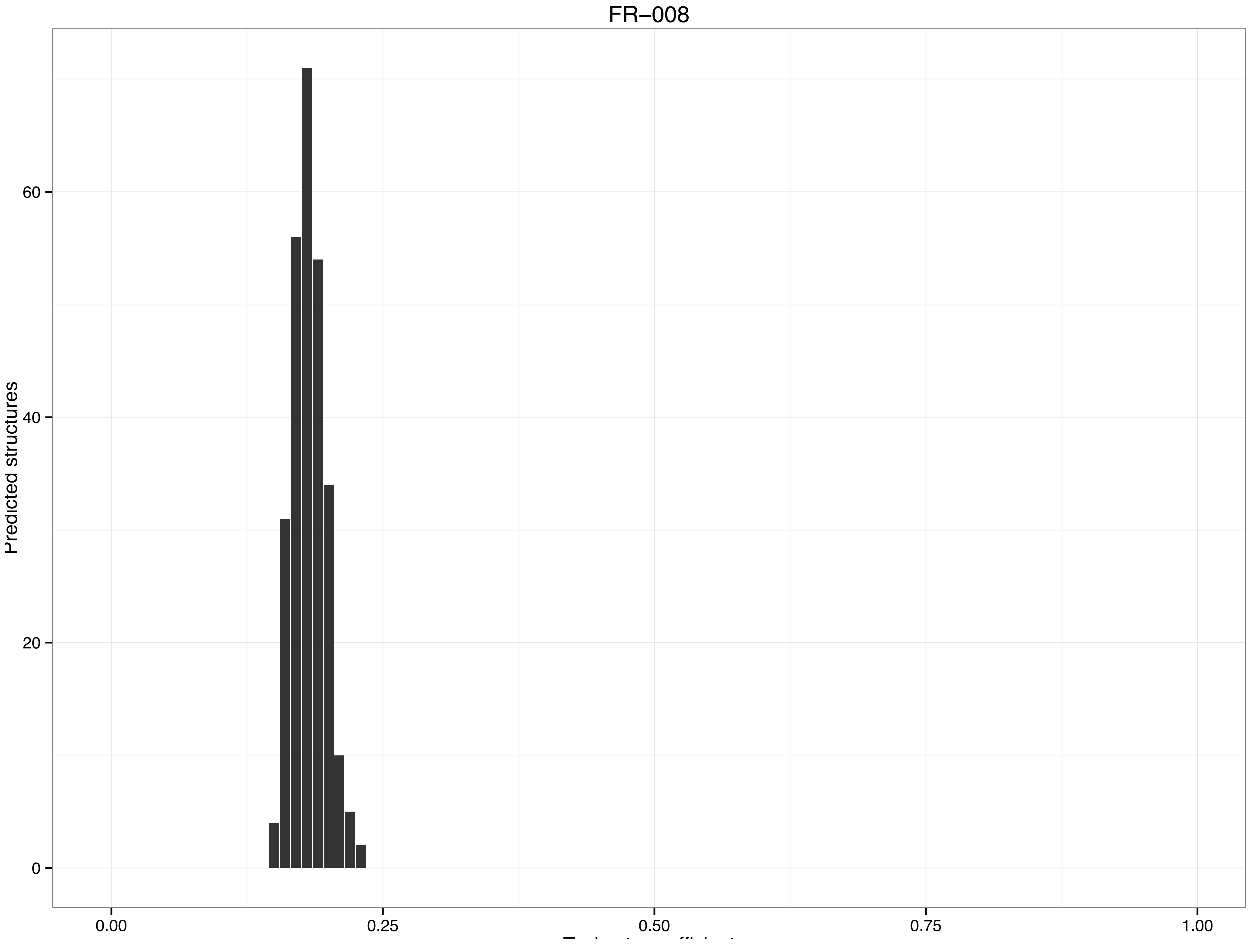
FR−008
Predicted structures
0
20
40
60
0.00
0.25
0.50
0.75
1.00

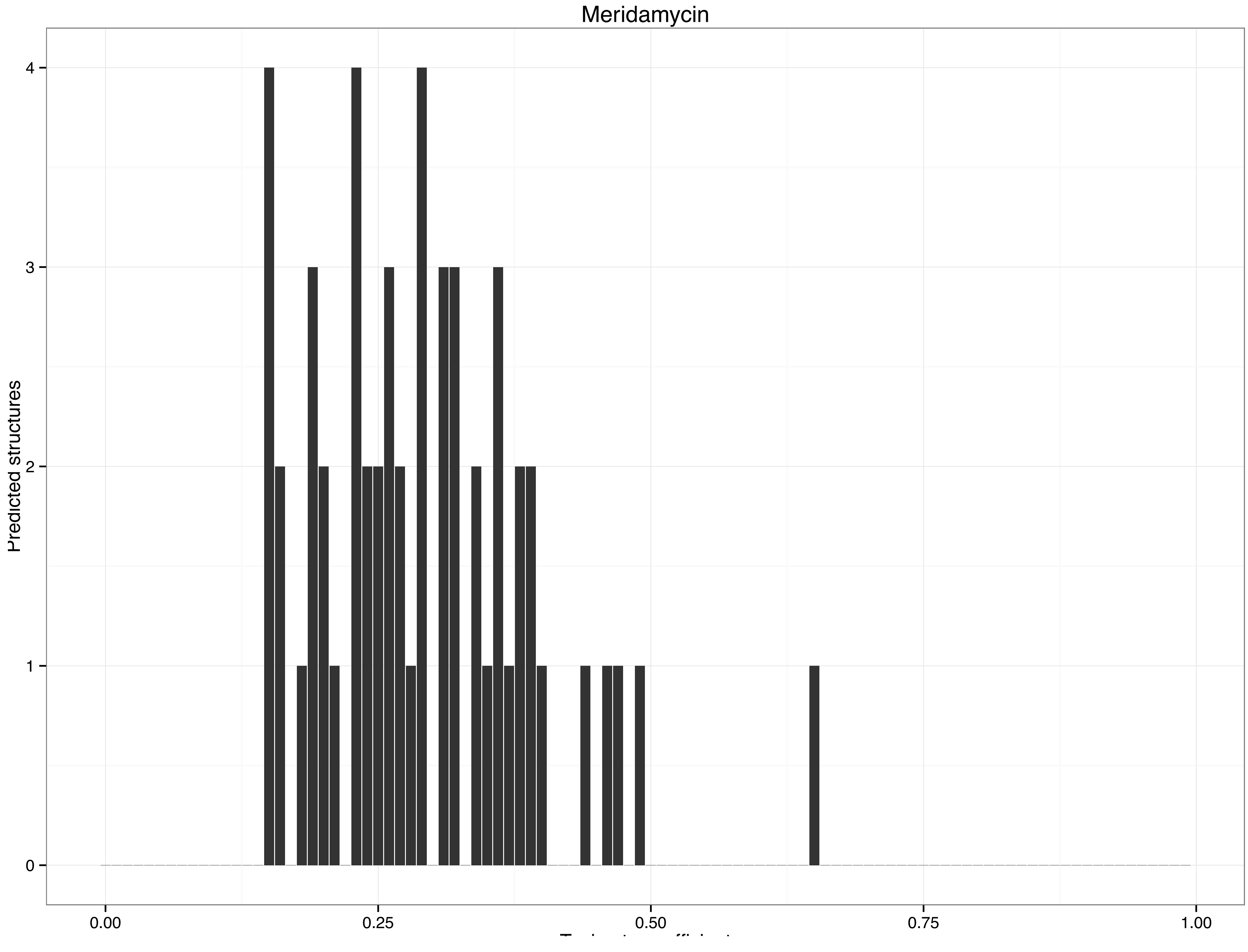
Meridamycin
Predicted structures
0
1
2
3
4
0.00
0.25
0.50
0.75
1.00

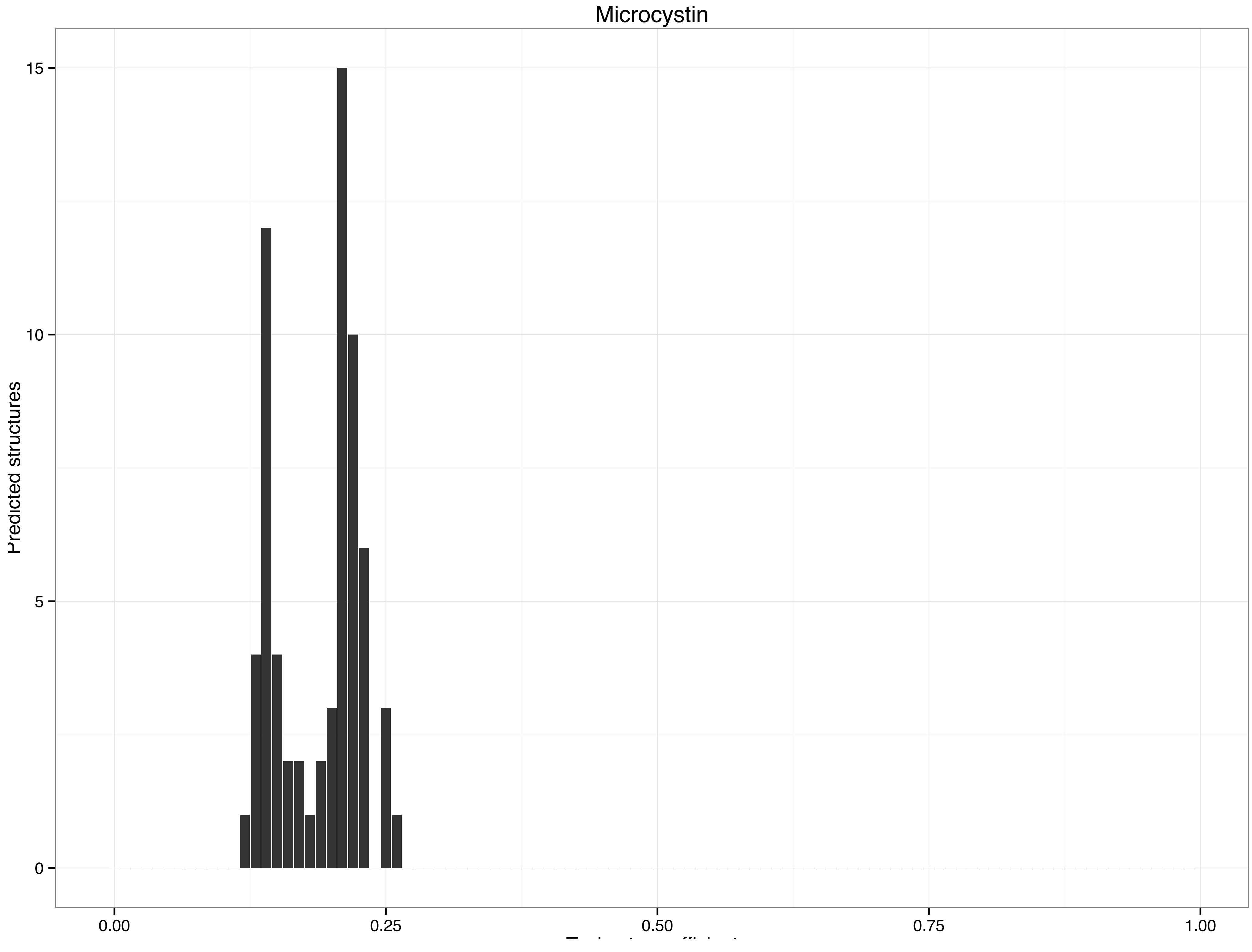
Microcystin
Predicted structures
15
10
5
0
0.00
0.25
0.50
0.75
1.00

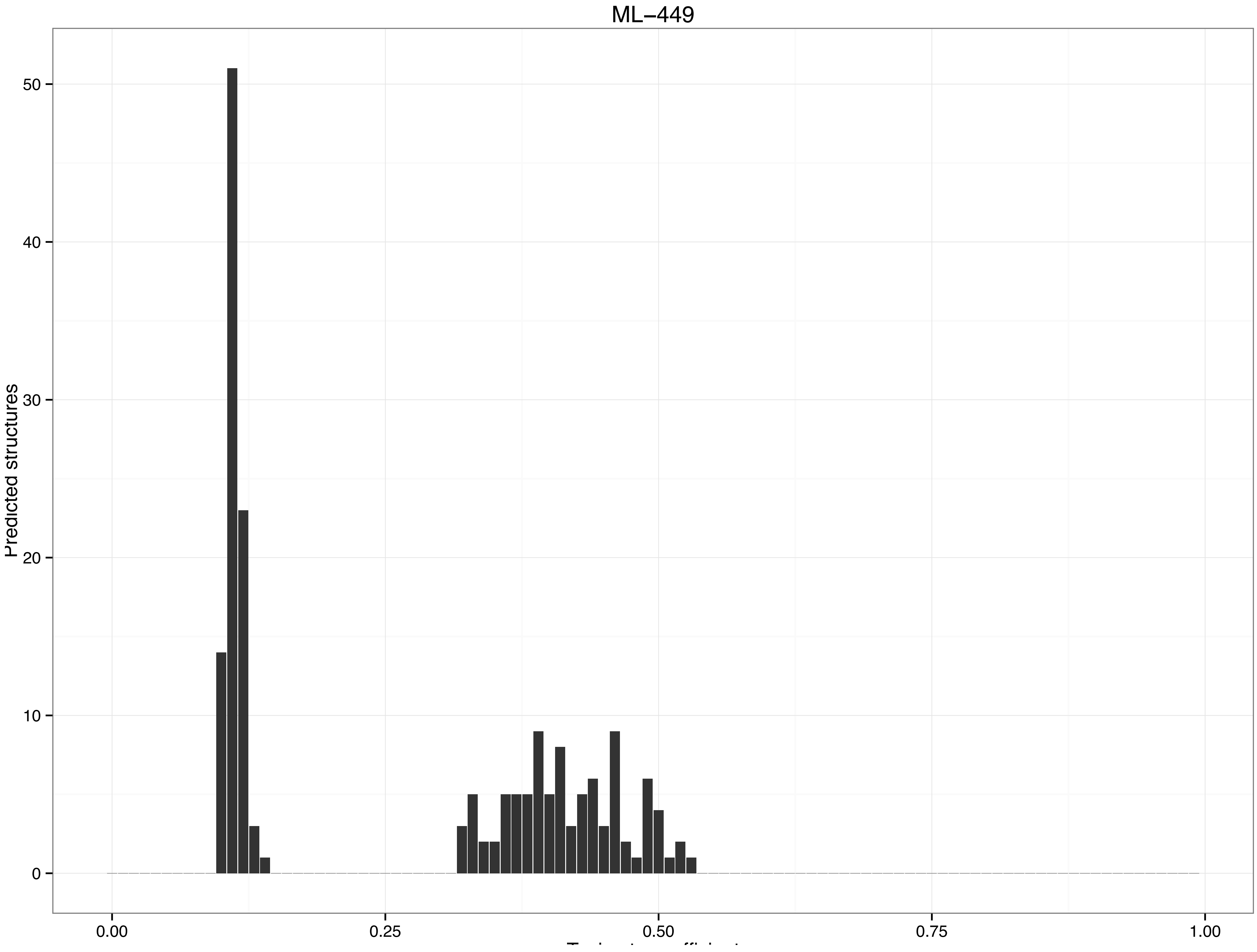

ML–449
Predicted structures
0
10
20
30
40
50
0.00
0.25
0.50
0.75
1.00

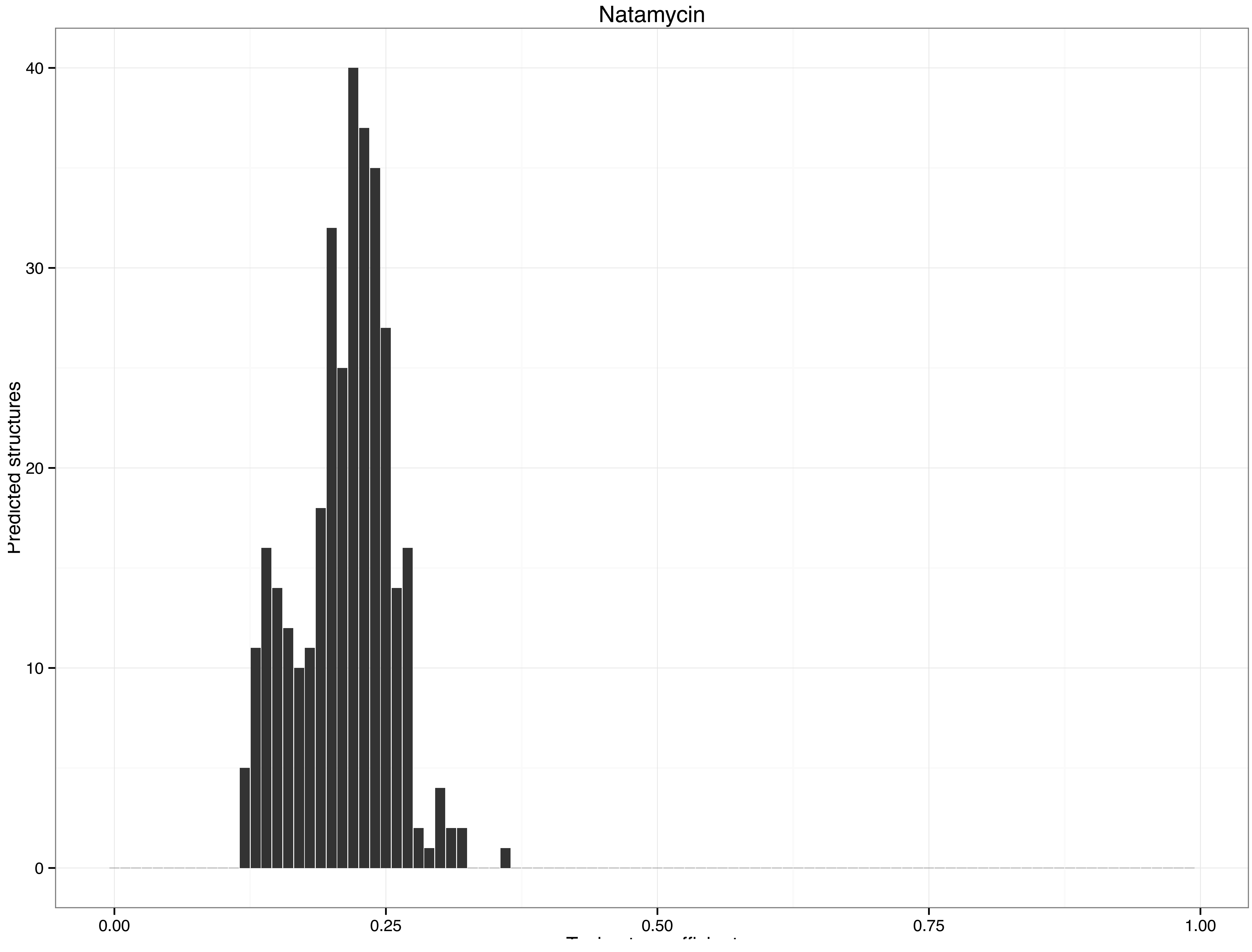
Natamycin
Predicted structures
40
30
20
10
0
0.00
0.25
0.50
0.75
1.00

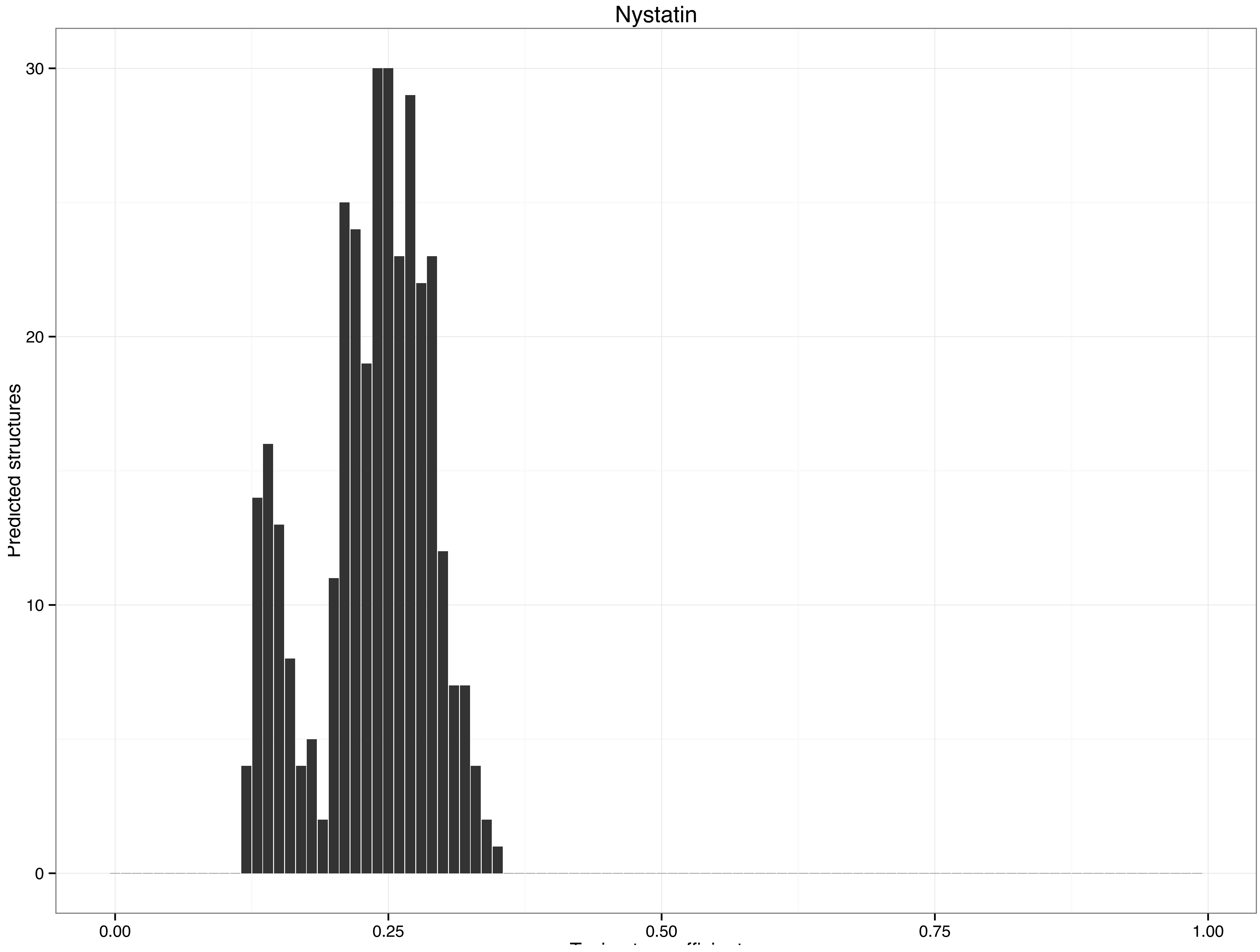
Nystatin
Predicted structures
30
20
10
0
0.00
0.25
0.50
0.75
1.00

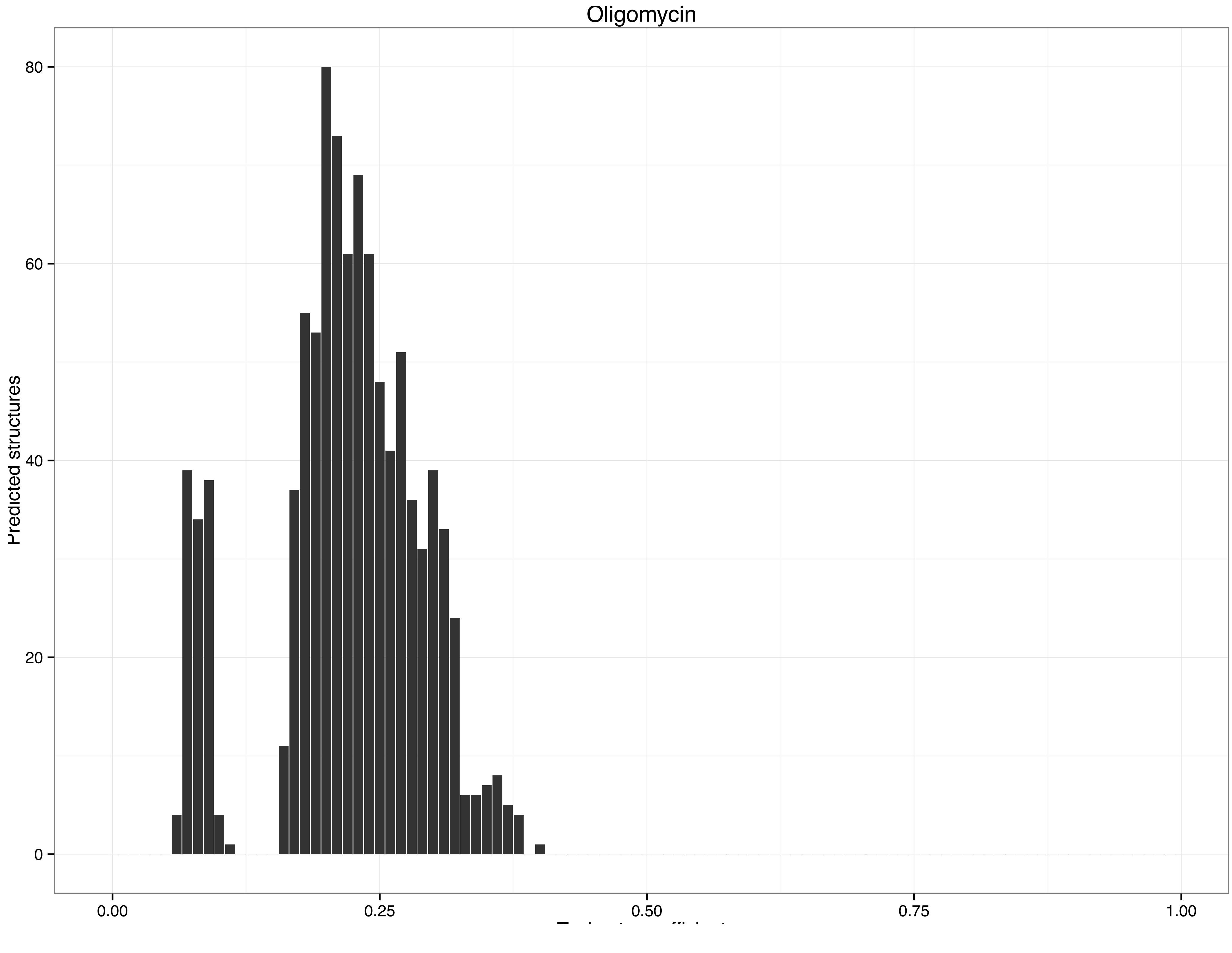

Oligomycin
Predicted structures
80
60
40
20
0
0.00
0.25
0.50
0.75
1.00

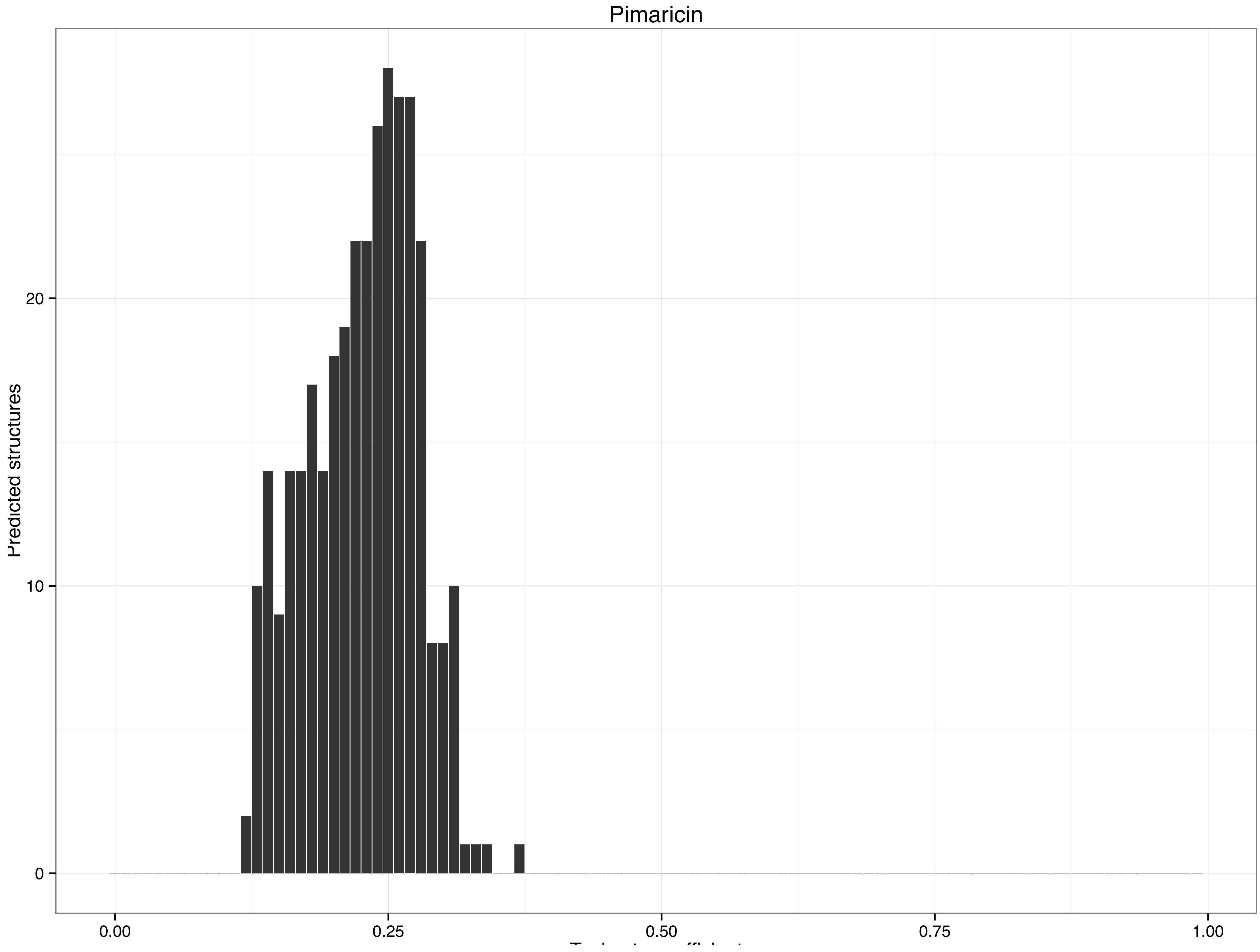
Pimaricin
Predicted structures
0
10
20
0.00
0.25
0.50
0.75
1.00

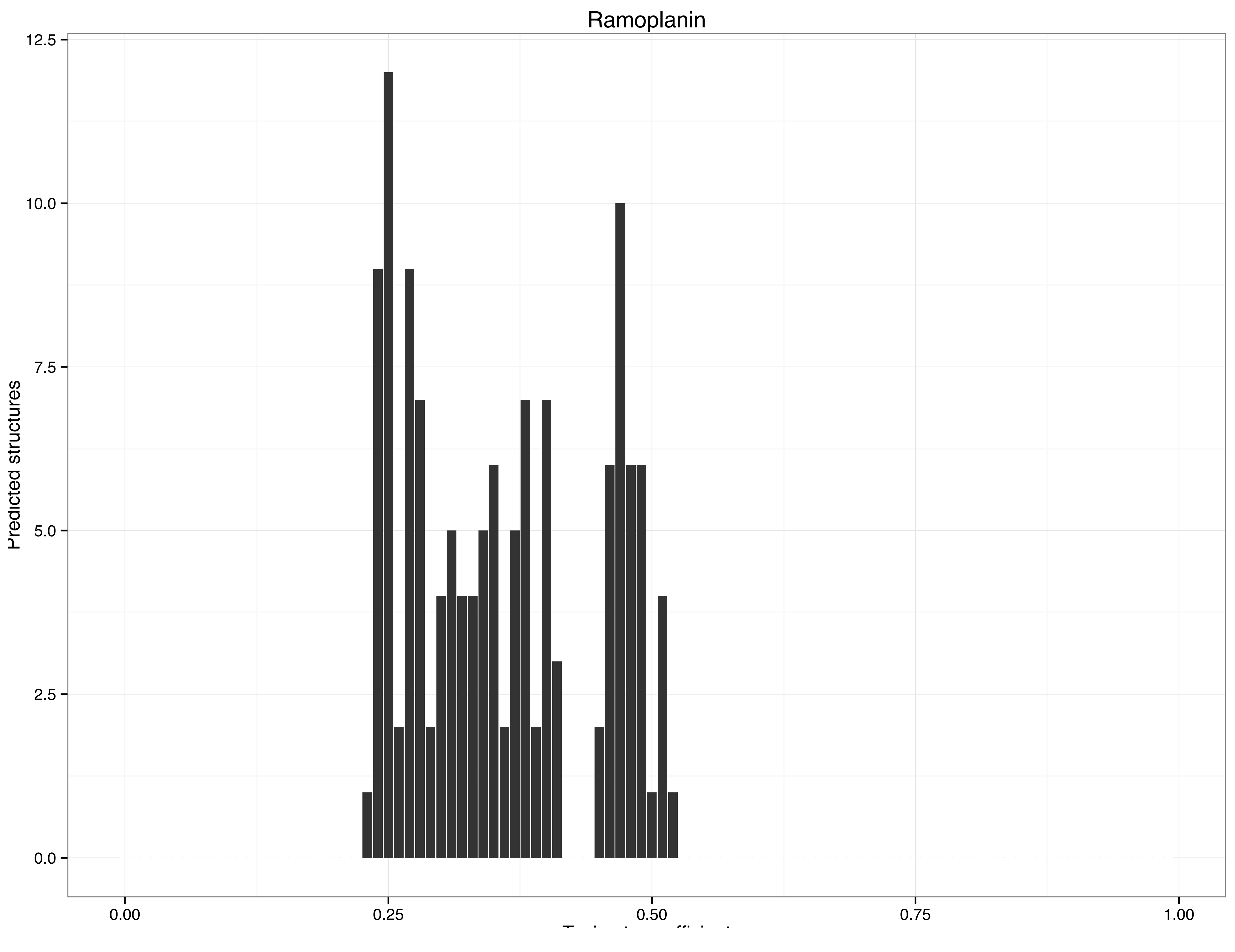
Ramoplanin
Predicted structures
12.5
10.0
7.5
5.0
2.5
0.0
0.00
0.25
0.50
0.75
1.00

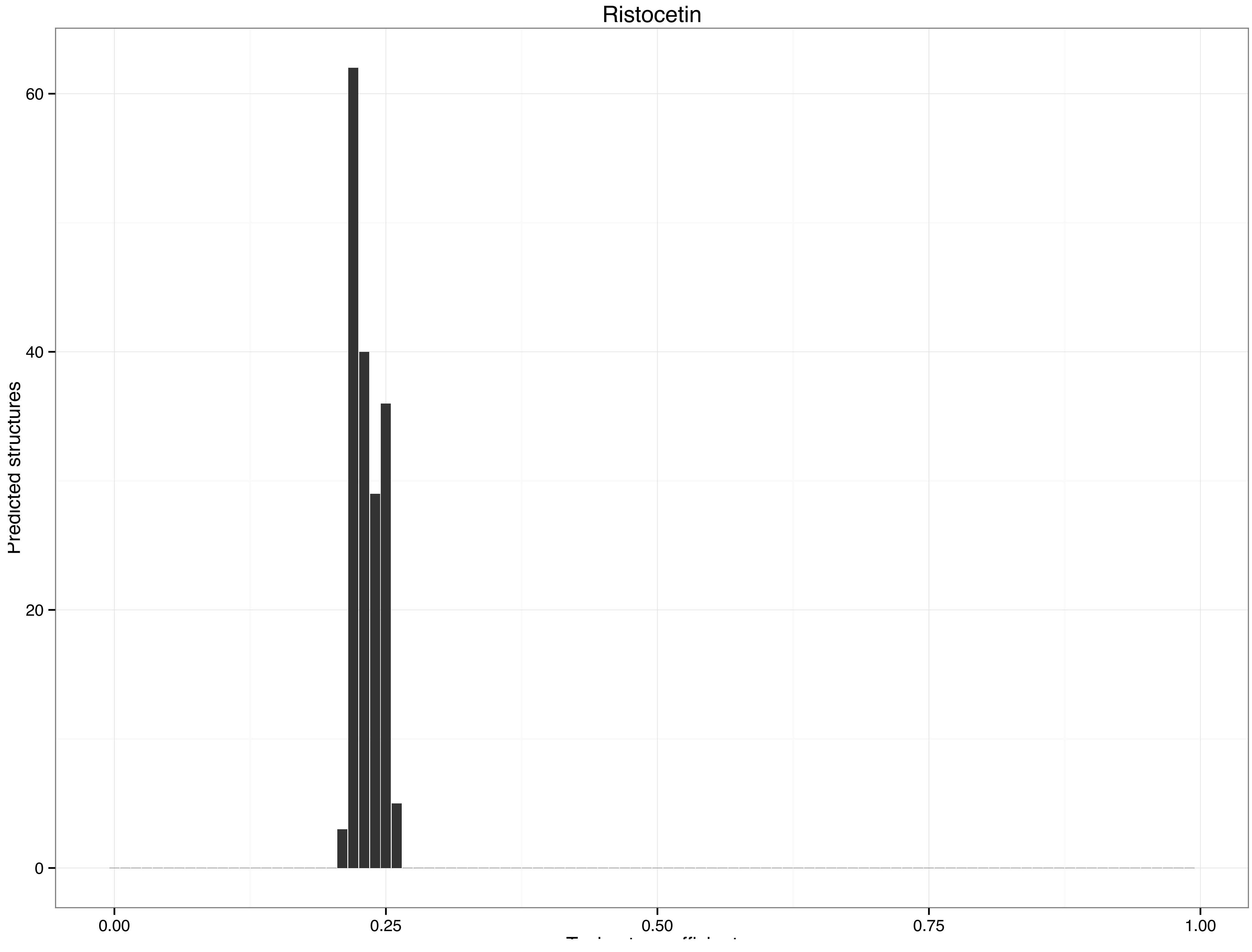
Ristocetin
Predicted structures
60
40
20
0
0.00
0.25
0.50
0.75
1.00

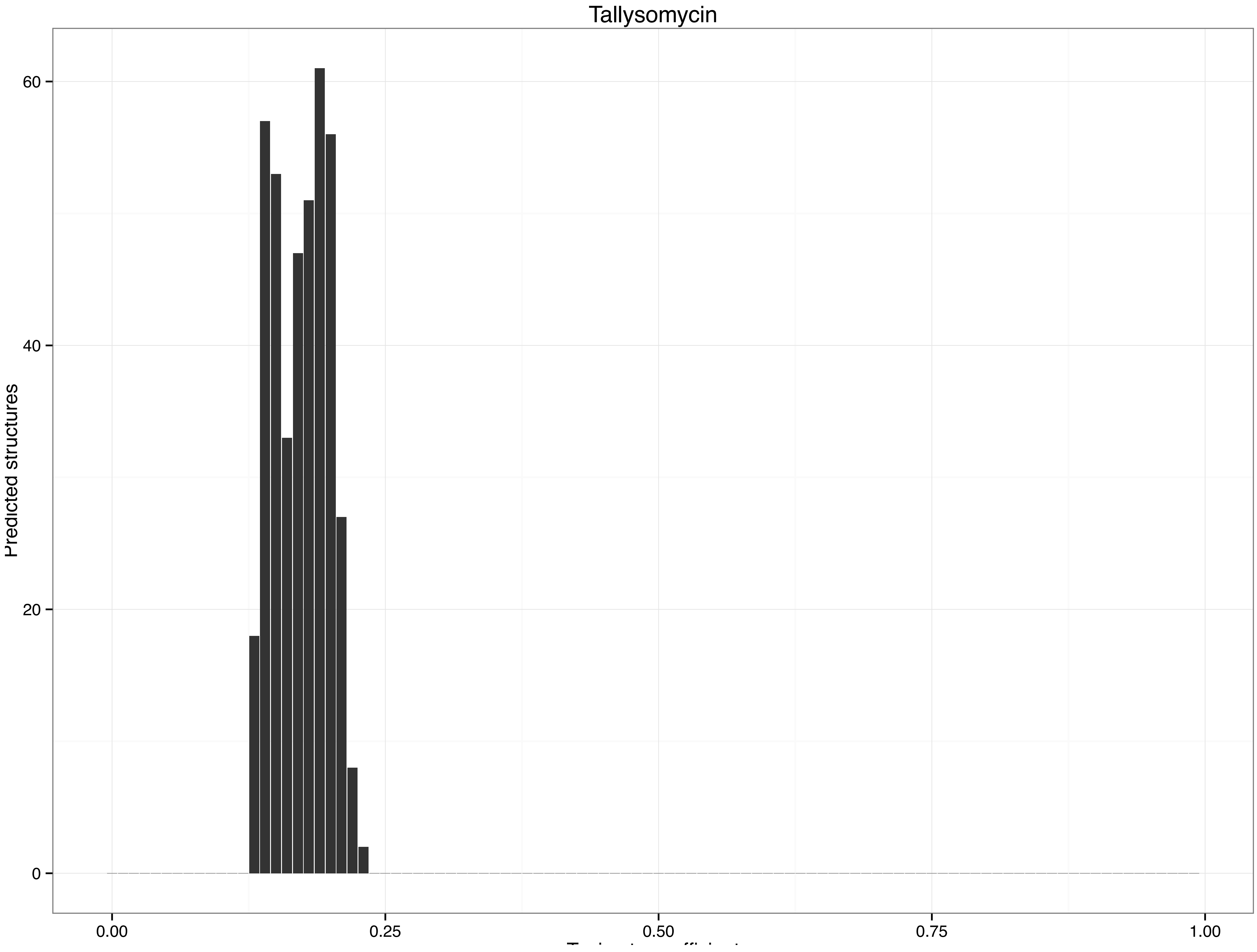

Tallysomycin
Predicted structures
60
40
20
0
0.00
0.25
0.50
0.75
1.00

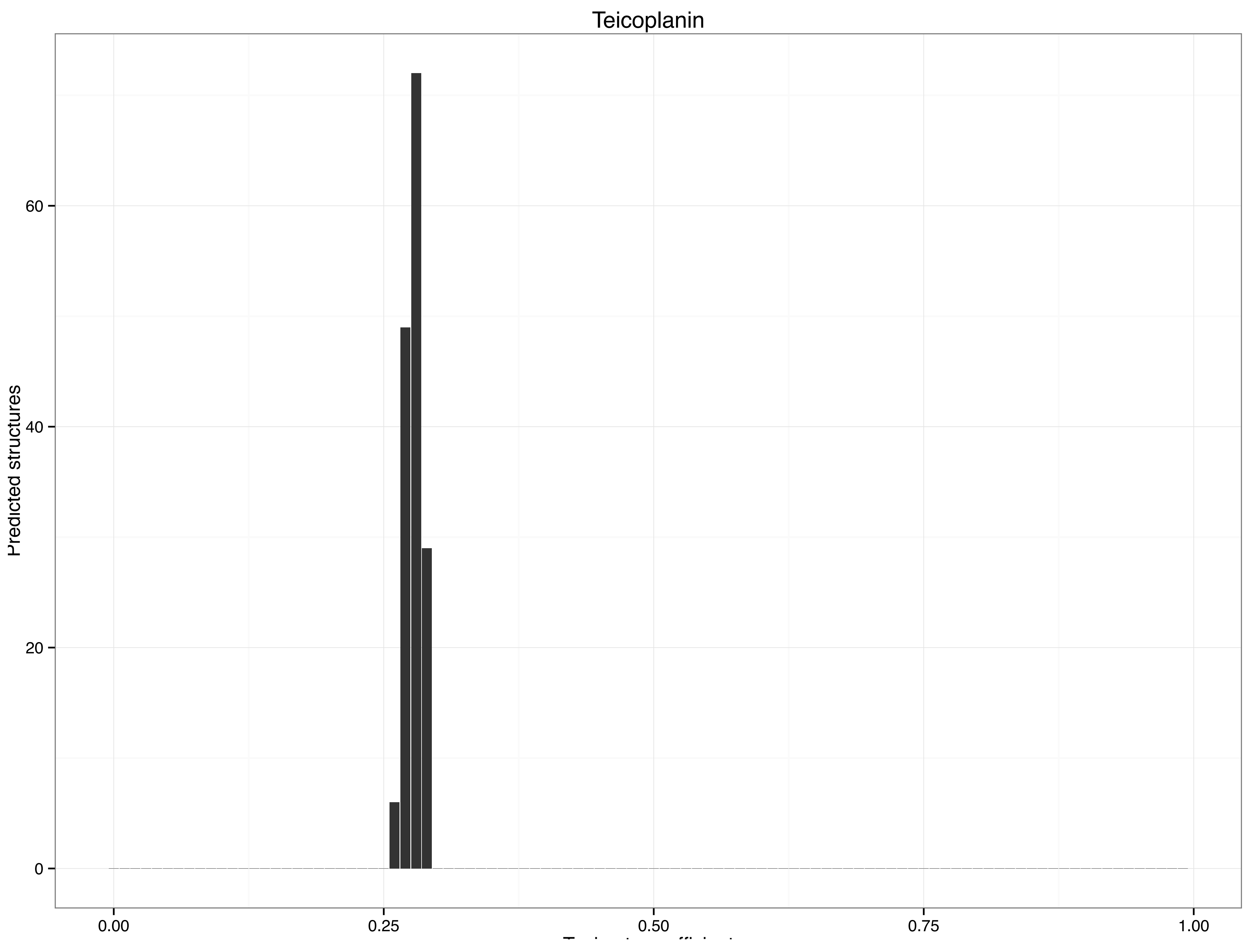
Teicoplanin
Predicted structures
60
40
20
0
0.00
0.25
0.50
0.75
1.00

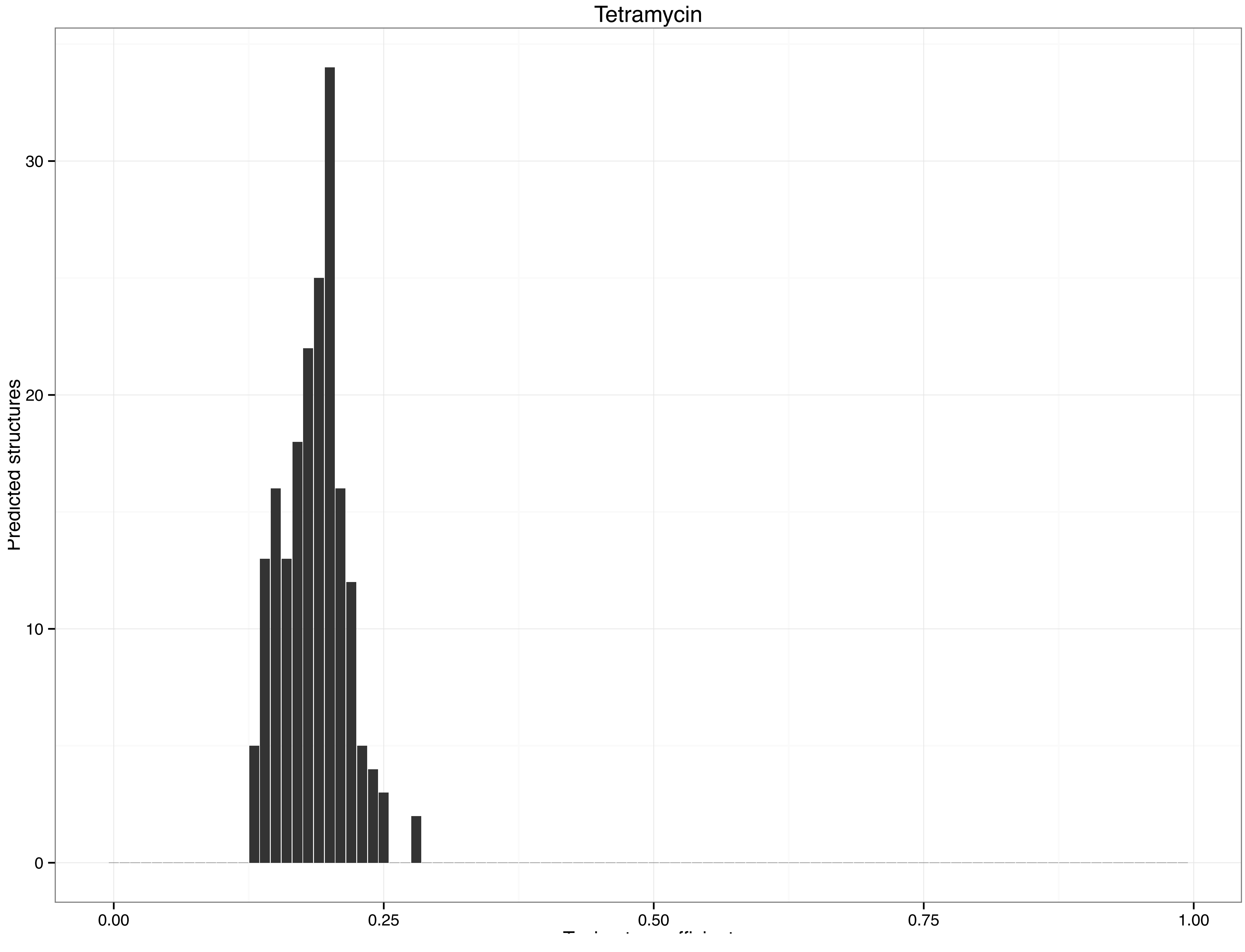
Tetramycin
Predicted structures
0
10
20
30
0.00
0.25
0.50
0.75
1.00

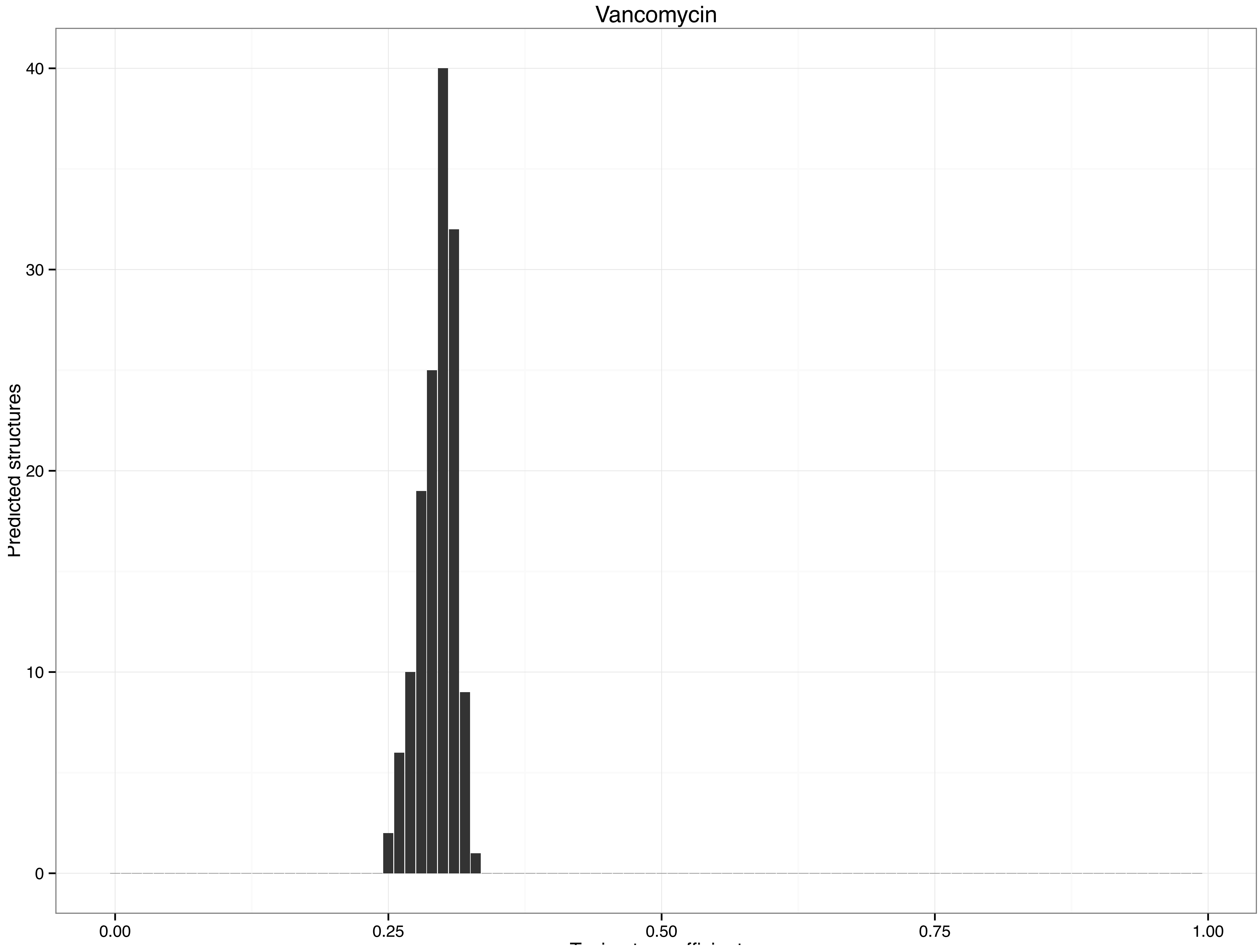
Vancomycin
Predicted structures
40
30
20
10
0
0.00
0.25
0.50
0.75
1.00

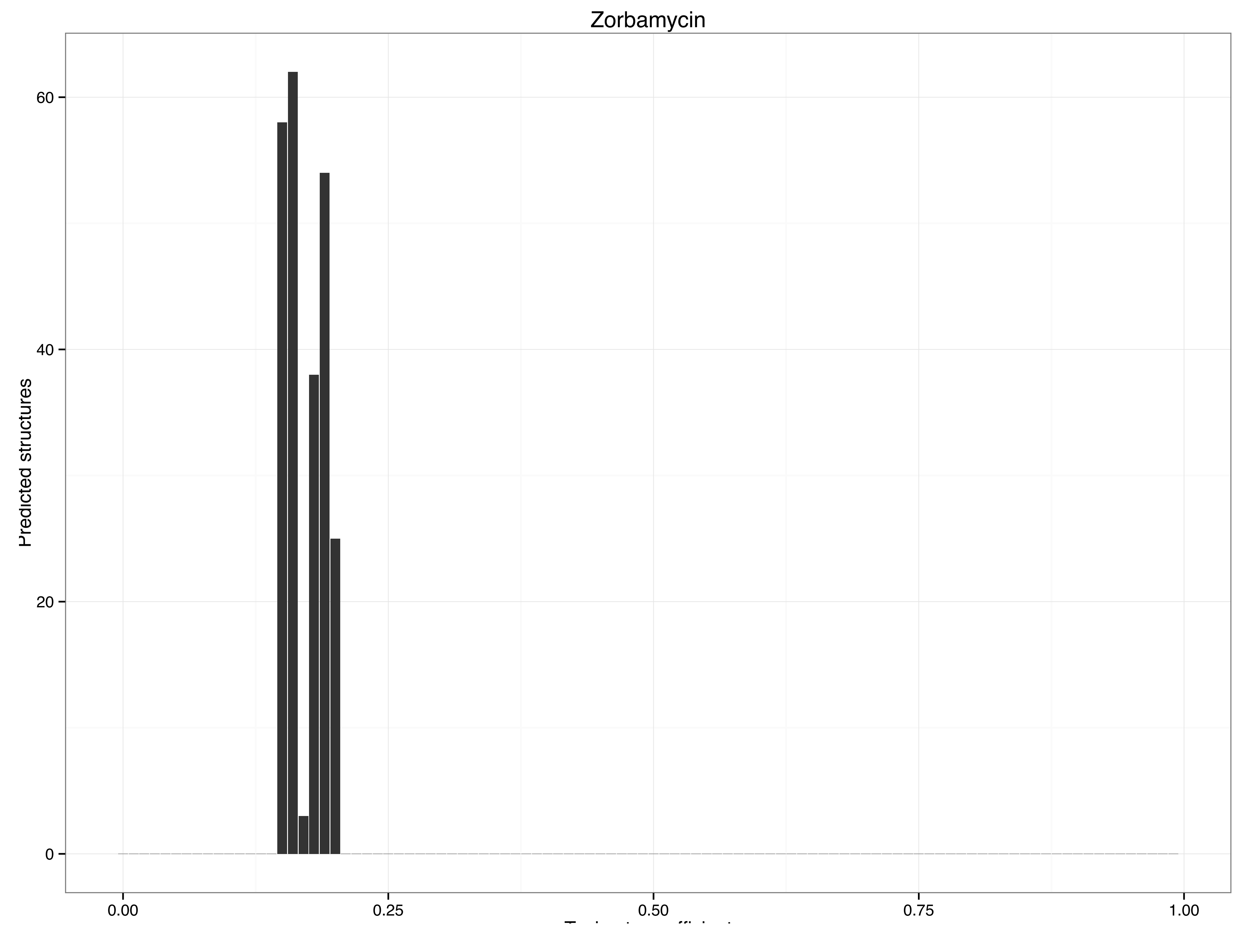

Zorbamycin
Predicted structures
0
20
40
60
0.00
0.25
0.50
0.75
1.00
