# Supplementary figures and images for: Genomes to natural products PRediction Informatics for Secondary Metabolomes (PRISM)
Source: Nucleic Acids Res. 2015 Oct 5;43(20):9645–62. doi: 10.1093/nar/gkv1012 (PMC4787774; doi:10.1093/nar/gkv1012)

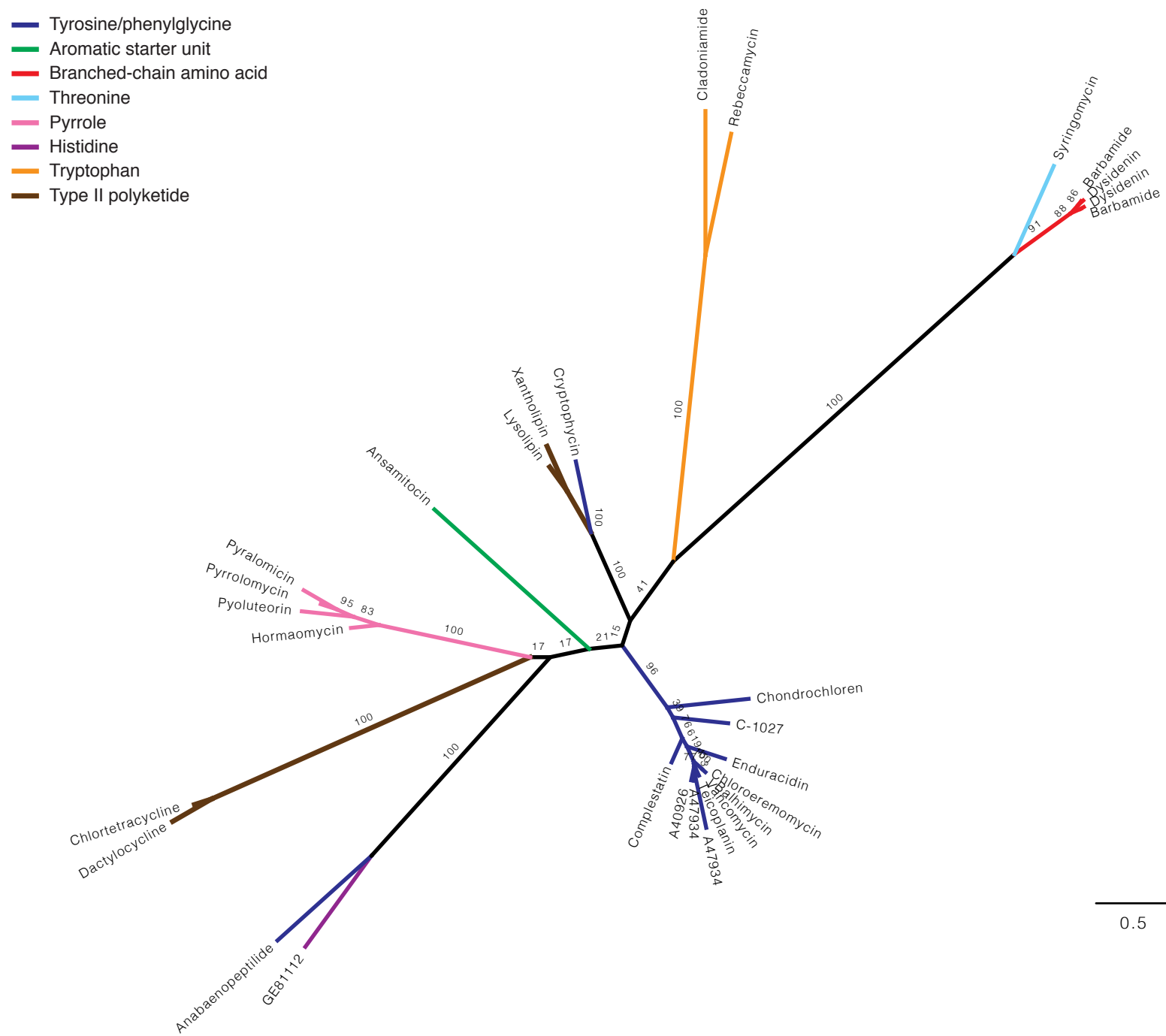

Supplement: SUPPLEMENTARY DATA [file supp_gkv1012_nar-01872-z-2015-File019.pdf]

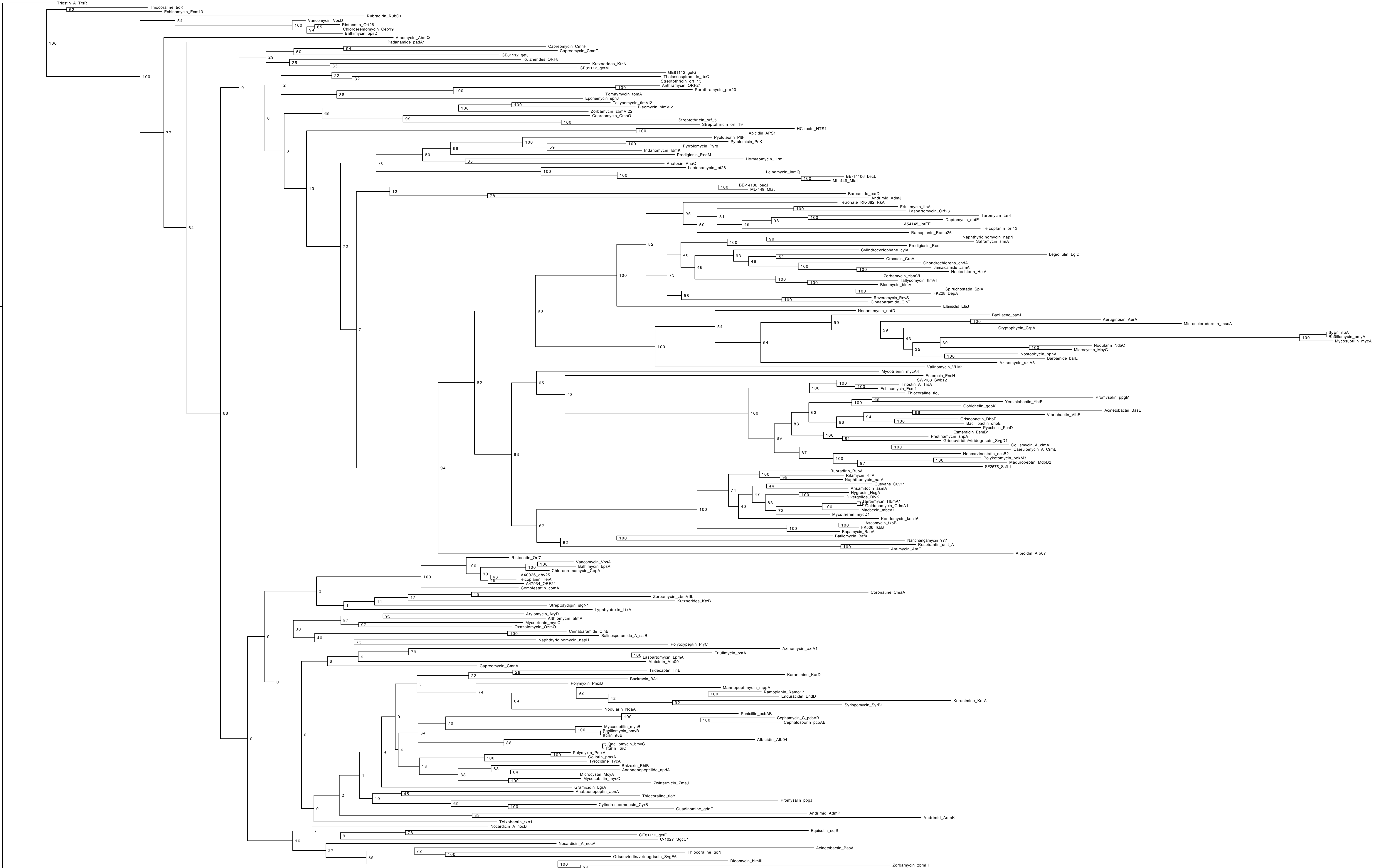

Supplement: SUPPLEMENTARY DATA [file supp_gkv1012_nar-01872-z-2015-File021.pdf]
