# Supplementary material for: Genomes to natural products PRediction Informatics for Secondary Metabolomes (PRISM)
Source: Nucleic Acids Res. 2015 Oct 5;43(20):9645–62. doi: 10.1093/nar/gkv1012 (PMC4787774; doi:10.1093/nar/gkv1012)
Supplement: SUPPLEMENTARY DATA [file supp_gkv1012_nar-01872-z-2015-File020.pdf]

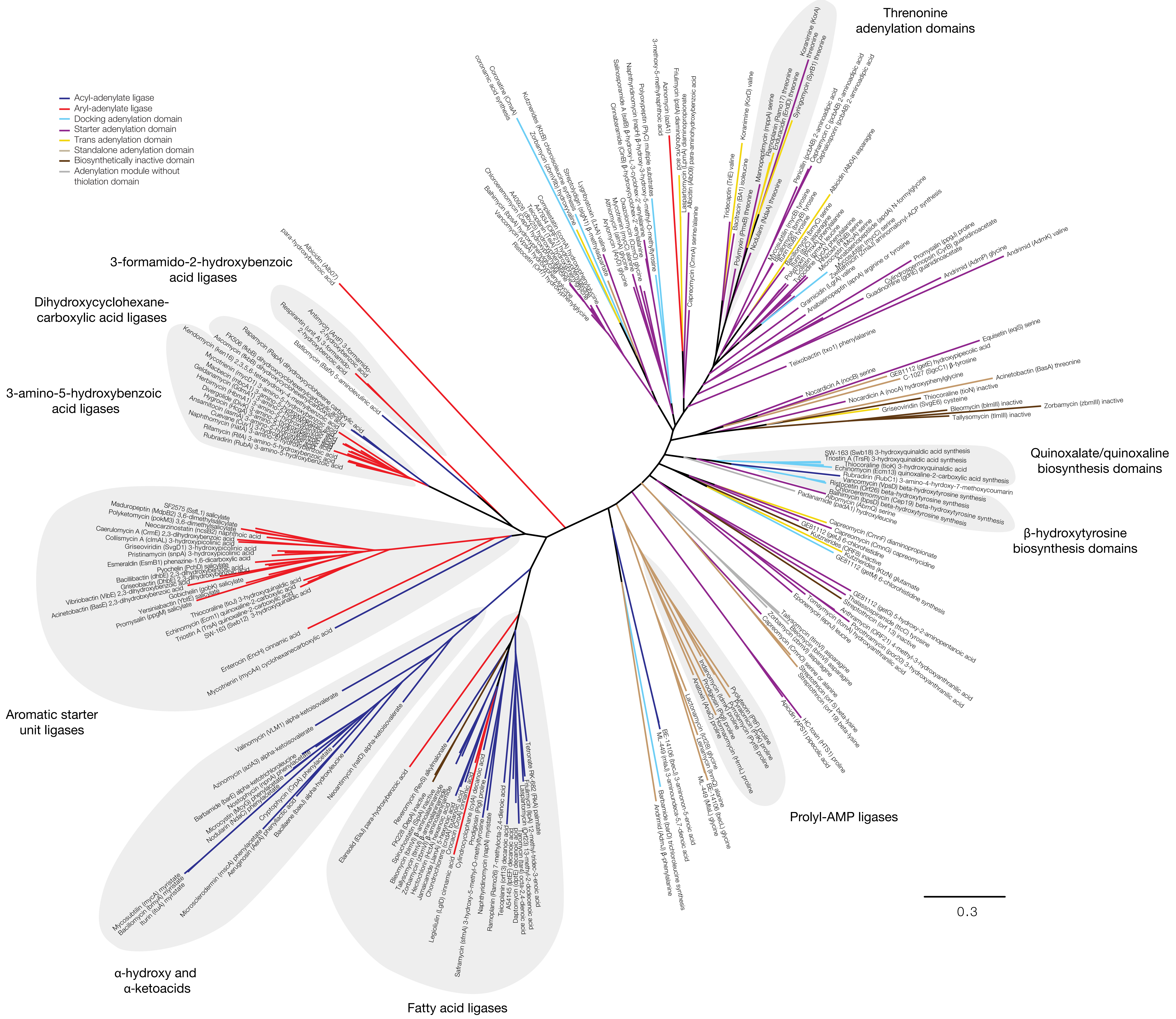
Acyl-adenylate ligase
Aryl-adenylate ligase
Docking adenylation domain
Starter adenylation domain
Trans adenylation domain
Standalone adenylation domain
Biosynthetically inactive domain
Adenylation module without thiolation domain
Threnonine adenylation domains
3-formamido-2-hydroxybenzoic acid ligases
Dihydroxycyclohexane-carboxylic acid ligases
3-amino-5-hydroxybenzoic acid ligases
Quinoxalate/quinoxaline biosynthesis domains
β-hydroxytyrosine biosynthesis domains
Aromatic starter unit ligases
Prolyl-AMP ligases
α-hydroxy and α-ketoacids
Fatty acid ligases
0.3
