# Supplementary material for: Genomes to natural products PRediction Informatics for Secondary Metabolomes (PRISM)
Source: Nucleic Acids Res. 2015 Oct 5;43(20):9645–62. doi: 10.1093/nar/gkv1012 (PMC4787774; doi:10.1093/nar/gkv1012)
Supplement: SUPPLEMENTARY DATA [file supp_gkv1012_nar-01872-z-2015-File022.docx]

|  | PRISM | NP.searcher | antiSMASH 3.0 |
| --- | --- | --- | --- |
| **Biosynthetic gene cluster analysis** |  |  |  |
| Nonribosomal peptide structure prediction | **Yes** | **Yes** | **Yes** |
| Type I polyketide structure prediction | **Yes** | **Yes** | **Yes** |
| Trans-AT type I polyketide structure prediction | **Yes** | No | **Yes** |
| Iterative type I polyketide structure prediction | **No, but enediyne and fungal PKS clusters identified** | No | **No, but PKS clusters identified** |
| Type II polyketide structure prediction | **Yes** | No | No |
| Resistance determinant identifiation | **Yes (264 genes identified)** | No | No |
| Identification of conserved multi-gene operons | **Yes (sugars only)** | No | **Yes** |
| Rule-independent cluster identification | No | No | **Yes** |
| **Chemical structure prediction** |  |  |  |
| Adenylation substrates predicted | 51 | 42 | **54** |
| Acyltransferase substrates predicted | 8 | 4 | **16** |
| Acyl-adenylating enzyme substrates predicted | **26** | 0 | 0 |
| Biosynthetic pathway-based substrates predicted | **6** | 0 | 0 |
| Virtual reactions predicted | **57 (see Supplementary  Dataset 1)** | 4 (glycosyltransferase, dehydratase, enolreductase, ketoreductase) | 3 (dehydratase, enolreductase, ketoreductase) |
| Cyclization patterns predicted | **5 (linear, macrolactam, macrolactone, imine, linear aldehyde)** | 2 (linear, macrolactone) | 1 (linear) |
| Sugars predicted | **67 (63 deoxy, 4 hexose)** | 1 (glucose) | 0 |
| Stereochemistry predicted | No | **Yes** | **Yes** |
| **Dereplication** |  |  |  |
| Bioinformatic (cluster-based) dereplication | **Yes** | No | **Yes** |
| Cheminformatic (structure-based) dereplication | **Yes** | No | No |

Supplementary Table 1. Comparison of features of open-source software for nonribosomal peptide and polyketide chemical structure prediction.
